# Synthesis, Pharmacological Evaluation, and Molecular Modeling of Phthalimide Derivatives as Monoamine Oxidase and Cholinesterase Dual Inhibitors

**DOI:** 10.1021/acsomega.4c10510

**Published:** 2025-03-04

**Authors:** Nabiha Abdullah, Fahad Hussain, Naseem Ullah, Humaira Fatima, Muhammad Afaq Tahir, Umer Rashid, Abbas Hassan

**Affiliations:** † Department of Pharmacy, Quaid-i-Azam University, Islamabad 45320, Pakistan; ‡ Department of Chemistry, Quaid-i-Azam University, Islamabad 45320, Pakistan; § Department of Chemistry, COMSATS University Islamabad, Abbottabad Campus, Abbottabad 22060, Pakistan; ∥ Institute of Pharmaceutical Sciences, University of Veterinary and Animal Sciences, Lahore 54000, Pakistan; ⊥ Department of Chemistry, College of Science, United Arab Emirates University, Al Ain, Abu Dhabi 15551, United Arab Emirates

## Abstract

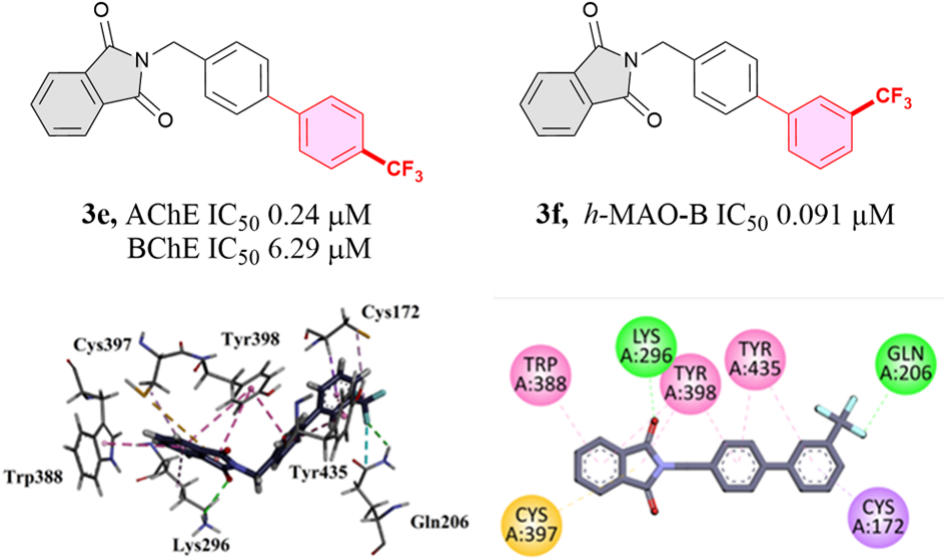

Alzheimer’s disease (AD) is a neurodegenerative disorder characterized by dementia and cognitive decline, associated with synaptic loss and degeneration of cholinergic neurons. New multitarget inhibitors for monoamine oxidase (MAO) and cholinesterase (ChE) enzymes are emerging as a potential treatment strategy for AD. Herein, we synthesized a series of *N*-benzyl-substituted biaryl phthalimide derivatives (**3a–3m**) encompassing potentially therapeutically active arenes/heteroarenes to serve as multitarget compounds for treating AD. To improve their binding affinity as well as inhibitory activity against ChE and MAO target proteins, comparable molecular structures were synthesized bearing electron-donating, electron-withdrawing, heterocyclic, and fluorinated moieties for a comprehensive SAR. In vitro evaluation of synthesized compounds against cholinesterases (AChE/BChE) and monoamine oxidases (MAO-A/MAO-B) revealed that compound **3e** had good potency against AChE (IC_50_ = 0.24 μM) and BChE (IC_50_ = 6.29 μM), while compound **3f** had the highest inhibition of MAO-B (IC_50_ = 0.09 μM). Selected compounds (**3e,f**) showed no cytotoxicity against the neuroblastoma cell line (SH-SY5Y) and normal human embryonic HEK-293 cells. Moreover, they showed high blood–brain barrier penetration (PAMPA assay) and reversible MAO-B inhibitory activity (ex vivo). In molecular docking studies, compounds **3e** and **3f** displayed the highest binding affinity with ChEs and MAO-B, respectively. In silico ADMET studies and MD simulation studies were also carried out for the most potent derivatives (**3e** and **3f**), suggesting their strong potential as anti-Alzheimer agents.

## Introduction

Alzheimer’s disease (AD) is an irreversible, incurable, degenerative neurological disorder associated with the loss of memory, functional and cognitive insufficiency, and behavioral transitions.^1^ As of 2021, AD is the seventh among the top 10 leading causes of mortality. More than 55 million people suffer from dementia worldwide, according to the WHO, and AD accounts for 60–70% of dementia. By 2050, this number is expected to become 139 million.^2^ The characteristic features of AD include amyloid β (Aβ) plaques and neurofibrillary tangles.^3^ These progressive changes can accompany hyperphosphorylation of tau protein, loss of cholinergic neurons, oxidative stress, metabolic and neuroinflammatory pathologies,^4^ and other comorbidities such as cardiovascular diseases and sleep disturbances.^4^ Among these, impairment of the generalized cholinergic function of the CNS is a significant contributing factor of AD, which in turn is associated with the decreased synthesis of acetylcholine, a neurotransmitter regulated by cholinesterase (ChEs). ChEs include acetylcholinesterase (AChE) and butyrylcholinesterase (BChE), synthesized by neurons residing in the brainstem and basal forebrain with axonal projections throughout the brain.^5^ These ChEs and Aβ plaque depositions are also interrelated, as reflected in AD patients.^6^ The inhibition of AChE and BChE serves as a potential target for treatment and delaying the progression of a group of neurological diseases, including AD.^7^

FDA-approved treatment strategies for mild to moderate AD include acetylcholinesterase inhibitors (donepezil, rivastigmine, and galantamine). For moderate to severe AD, antagonists of the *N*-methyl-*D*-aspartate receptor, memantine, and a combination therapy of donepezil and memantine are presently used. All of these agents target neurotransmitters, including acetylcholine and glutamate. The balancing of these neurotransmitters is required for the proper functioning of the CNS.

The mammalian monoamine oxidases (MAOs) are FAD-containing enzymes comprising two isoforms, MAO-A and MAO-B, responsible for the catalytic oxidative deamination of endogenous and exogenous amines in central and peripheral nervous systems.^8^ These isoforms have a slightly different conformation of cavity-shaping loop 210-216, which is ultimately responsible for their selectivity and sensitivity.^9^ MAO-A catalyzes serotonin^10^ and norepinephrine;^11^ its antagonists are treatment strategies for depression. MAO-B is responsible for the deamination of phenylethylamine and benzylamine;^12^ its inhibitors, in particular, are reported to be responsible for increased dopamine levels and provide neuroprotective effects. MAO-B is present in basal ganglia, and its levels increase with age. The increased MAO-B levels result in increased neurodegeneration in the elderly. In addition, aberrant astrocytic γ-aminobutyric acid (GABA) released by MAO-B causes memory impairments in animal models.^13^ These considerations make MAO-B a potential therapeutic target for AD. The irreversible inhibitors of MAO-B (selegiline and rasagiline) are being prescribed for reducing the progression of AD and restoration of memory function. However, long-term treatment with selegiline led to insignificant improvements in cognitive deficits and was suggested to eventually destroy the enzyme conformation. Conversely, the reversible inhibitors are of prime importance in terms of tyramine metabolism^14^ and quick recovery of enzyme function after treatment withdrawal. This makes reversible MAO-B inhibitors an attractive target for the treatment of AD, and thus, there is a pressing need for novel approaches.

Until 2021, only five drugs were FDA-approved for the treatment of AD after their discovery in 1906. Due to the complex pathophysiology and etiology of the disease, the “one-compound-one target” therapeutic rationale has largely been met with failure. These considerations have led us to the design of multitarget-directed ligands that modulate the disease using different target proteins, including AChE, BChE, and MAO-B. Researchers have shown increasing interest in the design of multitarget ligands that can simultaneously act on multiple targets of the disease pathway.

**Figure 1:**
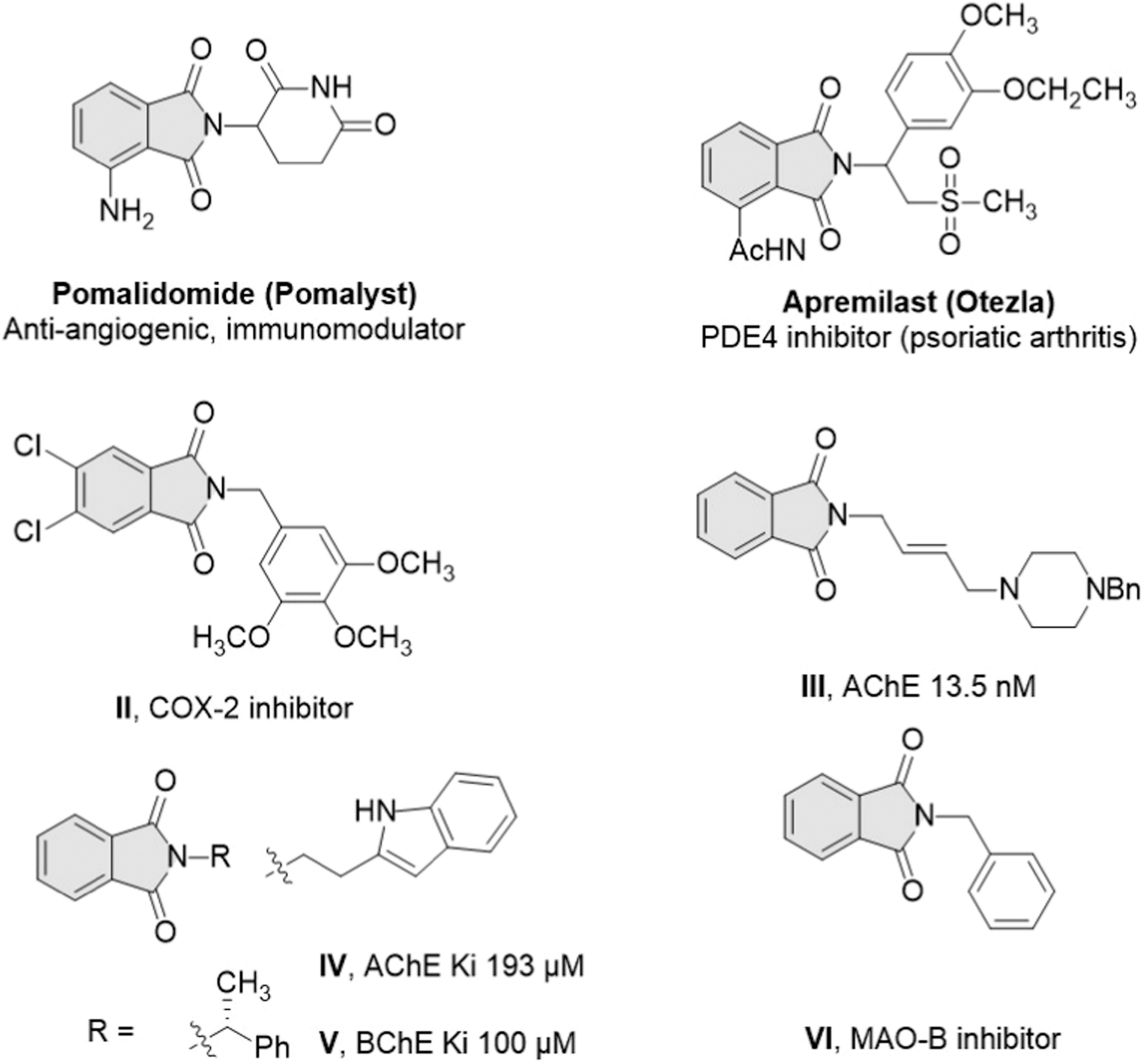
Selected examples of clinically approved and therapeutically active phthalimide derivatives.

Nitrogen and oxygen heterocycles (e.g., phthalimides chromones,^15^ indoles, and coumarins) have been explored for potential use in the treatment of AD.^16^ Among these, phthalimides make up a class of nitrogen-containing bicyclic hydrophobic compounds that can easily cross biological membranes and have high bioavailability. Due to their diverse pharmacological properties, their derivatives serve as valuable building blocks in medicinal chemistry. Researchers have synthesized nitrogen-substituted phthalimides to create more targeted and safer drugs such as antioxidant, antibacterial,^17^ antifungal and antimycobacterial agents,^18^ inhibitors of cyclo-oxygenase-2,^19^ cholinesterase,^20^ and monoamine oxidase-B enzymes.^21^ (Figure 1). One clinical example is apremilast (Otezla), a phosphodiesterase 4 inhibitor approved by the FDA in 2021 as the first and only oral agent for the treatment of all severities of plaque psoriasis. It acts by modulating inflammatory mediators and was approved for active psoriatic arthritis in adult patients in 2014 (Figure 1).^22^

Pomalidomide, sold under trade names Pomalyst and Imnovid, was approved by the FDA in the US and EU in 2013 as an antiangiogenic and immunomodulatory agent. It is an *N*-substituted phthalimide derivative and a promising candidate for the treatment of multiple myeloma and AIDS-related Kaposi sarcoma.^23^ Phloroglucinol trimethyl ether phthalimide derivative (compound **I**) has potent anti-inflammatory activity via selective COX-2 inhibition (IC_50_ = 0.18 μM) compared to celecoxib, as reported by Amer Alanazi et al.^19^

Numerous exciting examples of phthalimide-based AChE inhibitors are reported in the literature. A piperazine phthalimide derivative, compound **II**, was developed as an inhibitor of AChE with an IC_50_ value of 13.5 nM. Similarly, the phthalimide derivatives **III** and **IV** served as inhibitors of AChE and BChE with enzyme inhibition dissociation constants (K*i* value) of 193 and 100 μM, respectively (Figure 1).^24^ The simple *N*-benzylphthalimide **V** was found to show weak MAO-B inhibition with an IC_50_ value of 10.5 μM compared to that of the standard.

Dual inhibitory activity against both ChE and MAO enzymes is a potential treatment strategy for AD; however, the difference in the binding pockets of both target proteins makes this a challenging task.^25^ The weak inhibitory activity and binding affinity of substituted phthalimide for ChEs and MAO-B enzymes can potentially be improved with SAR studies, and therefore, we have devised a plan to synthesize *N*-benzyl-substituted phthalimides bearing electron-donating, electron-withdrawing, heterocyclic, and fluorinated moieties. The increase in the hydrophobic character by the addition of lipophilic substituents and the increase in the chain length may ultimately be responsible for improved binding interactions with the target proteins. The compounds were evaluated for their in vitro AChE, BChE, MAO-A, and MAO-B inhibition potential, neurotoxicity, MAO reversibility, and BBB permeability. Further, advanced computational methods were employed, including molecular docking, ADME profiling, and molecular dynamics simulations, which elucidated the binding affinities and pharmacokinetic properties of these compounds. In-vitro enzymatic assays and in-silico analysis revealed significant potential of newly designed derivatives as viable candidates for further development in the treatment of Alzheimer’s disease.

## Results and Discussion

### Chemistry

**Scheme 1:**
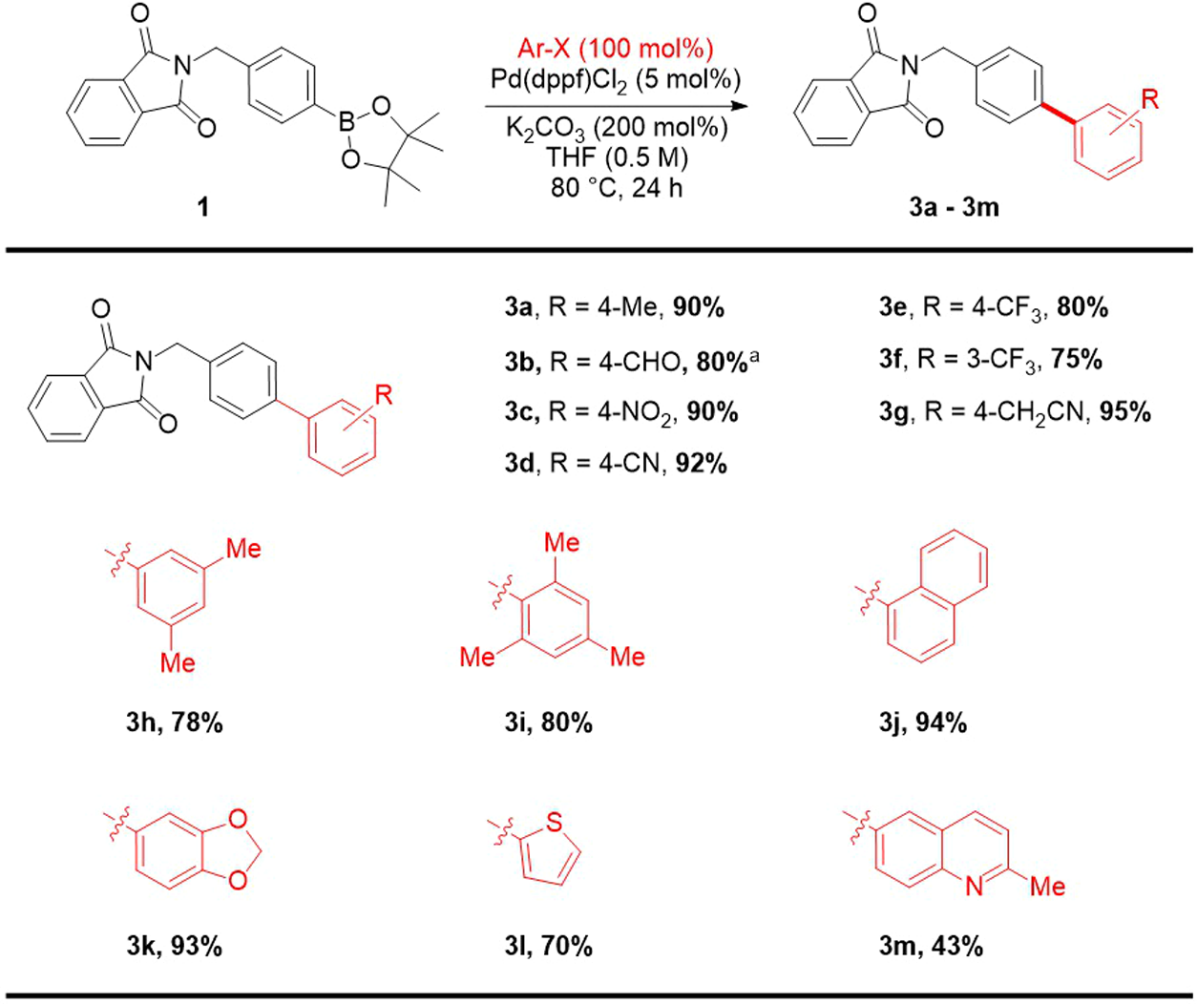
Synthesis of Phthalimido-Substituted Biphenyl Derivatives Reaction time was 4 h.

The synthesis commences from the readily available 4-(*N*-phthalimidomethyl)benzeneboronic acid pinacol ester **1**, which was subjected to Suzuki coupling with aryl halides **(2a-2m)** resulting in derivatives (3a–3m) (Scheme 1). Various reaction conditions were employed for the coupling reaction using Pd(0) and Pd(II) catalysts, including Pd(PPh_3_)_4_, Pd(dppf)Cl_2_, and Pd(OAc)_2_. The Pd(dppf)Cl_2_ was found to be the best in combination with potassium carbonate as a base in dry THF and turned out to be the optimal reaction conditions for diverse substrates. A broad range of aryl iodides and bromides were selected for the corresponding transformation of biaryl derivatives providing the advantages of mild conditions, reduced reaction time, and low catalyst loadings.^26^

The substrates comprised monosubstituted **(2a–2g)**, disubstituted **(2h)**, trisubstituted **(2i)**, and heterocyclic **(2j–2m)** aryl moieties to study the effect of different substitutions on the biological activity (Scheme 1). Reaction with aryl iodides resulted in the formation of compounds **3d** and **3l**, while the remaining compounds (**3a–3c, 3e–3k, and 3m)** were obtained by employing aryl bromides. Using optimized conditions, the coupled products were obtained in good to excellent isolated yields (70–95%) except compound **3m**, which was obtained in only 43% yield.

The substitutions on the aryl moiety also contribute to the reaction progress. Aryl halides bearing electron-withdrawing groups are more reactive toward the rate-determining oxidative addition step compared to those having electron-donating groups. Detailed spectroscopic techniques, including FTIR, ^1^H, and ^13^C NMR, and mass-spectrometric analysis evidenced^27^ structure elucidation and purities of newly synthesized derivatives.

## Biological Study

### Cholinesterases Inhibition Assay

The synthesized compounds were evaluated for their inhibition potential against cholinesterases (*Electrophorus electricus* AChE and equine serum lyophilized BChE). Donepezil was used as the standard drug. Table 1 shows the results of enzyme inhibition in micromolar concentration (μM). The synthesized compounds (**3a–3m**) exhibited superior activity against *ee*AChE as compared to eqBChE. When compared to the positive control donepezil (IC_50_ = 0.054 μM), compound 3e exhibited the highest *ee*AChE inhibition (IC_50_ = 0.24 μM), followed by compounds **3j** (IC_50_ = 0.61 μM) and **3h** (IC_50_ = 0.73 μM). The linearity in the structure with hydrophobic moieties (phthalimide and substituted benzene) might play a crucial role in the inhibition results. Compound 3k was found to be the least potent, with IC_50_ = 21.13 μM and a selectivity index of 3.8.

**Table 1:** *In* Vitro Enzyme Inhibition Results of *ee*AChE, *eq*BChE, *h*MAO-A, and *h*MAO-B

	IC_50_ (μM) ± SEM^a^			IC_50_ (μM) ± SEM^a^		
compounds no.	*ee*AChE	*eq*BChE	SI^b^	*h*MAO-A	*h*MAO-B	SI^c^
3a	3.16 ± 0.09	49.11 ± 1.36	15	19.33 ± 1.28	1.04 ± 0.03	18
3b	na	na		na	na	
3c	0.94 ± 0.12	49.08 ± 1.39	52	19.37 ± 1.21	0.24 ± 0.01	80
3d	9.21 ± 0.14	33.54 ± 1.09	4	28.8 ± 1.44	0.64 ± 0.03	45
3e	0.24 ± 0.01	6.29 ± 0.17	26	16.51 ± 0.66	0.11 ± 0.01	150
3f	4.48 ± 0.11	17.03 ± 1.04	4	11.25 ± 0.20	0.091 ± 0.006	125
3g	13.43 ± 0.08	99.1 ± 1.21	7	5.44 ± 0.16	0.78 ± 0.04	7
3h	0.73 ± 0.04	29.58 ± 1.16	40	75.11 ± 1.25	0.64 ± 0.02	117
3i	0.88 ± 0.05	49.21 ± 1.11	56	50.91 ± 1.42	0.71 ± 0.04	71
3j	0.61 ± 0.03	18.91 ± 1.08	31	6.98 ± 0.20	0.13 ± 0.01	53
3k	21.13 ± 0.47	80.70 ± 1.54	4	40.37 ± 1.31	0.66 ± 0.03	61
3l	0.91 ± 0.02	55.25 ± 1.31	61	8.29 ± 0.33	0.14 ± 0.01	59
3m	0.89 ± 0.10	31.08 ± 0.79	35	23.49 ± 1.12	0.15 ± 0.01	156
donepezil	0.054 ± 0.001	5.4 ± 0.07	108			
safinamide				6.75 ± 0.44	0.025 ± 0.003	270

a ± SEM represents mean values; *n* = 3.

b SI = IC_50_ of BChE/IC_50_ of AChE; ppt = na = no activity found in the tested concentration.

c = SI (IC_50_ hMAO-A/IC_50_ hMAO-B).

On the other hand, the compounds exhibited moderate to low inhibition (in the submicromolar range) toward eqBChE, which might be due to the difference in the acyl pocket of both enzymes, which in turn is responsible for the difference in selectivity and speed of hydrolyzing their substrates; BChE can accommodate bigger substrates compared to AChE. Compound **3e**, which also showed the highest *ee*AChE inhibition, emerged as the most potent inhibitor of *eq*BChE (IC_50_ = 6.29 μM). This compound has a CF_3_ moiety at the para position of the phenyl ring, which is a lipophilic, electron-deficient moiety. Thus, the overall cholinesterase inhibition potential (against both *ee*AChE and eqBChE) of compound **3e** was the highest among all of the synthesized compounds. Therefore, compound **3e** was selected for further biological evaluation.

### Human MAO Isoform Inhibition Assay

The human MAO isoform assay was also carried out using a fluorescence-based assay protocol according to the reported literature. Safinamide was used as the standard drug for the assay. Compounds 3a–3m, except **3b**, displayed good inhibitory potential toward MAO-B. Among these, compound **3f** with an IC_50_ value of 0.091 μM exhibited 124-fold more potency toward MAO-B than MAO-A. The promising results can be linked to similarities in structure with safinamide hydrophobic and hydrophilic regions.

Regarding MAO-A inhibition, compounds **(3a–3m)** exhibited moderate to good inhibition activity against MAO-A. Compound **3g** containing a nitrile moiety on biphenyl series demonstrated the most potent activity with an IC_50_ value of 5.44 μM compared to safinamide (IC_50_ = 6.75 μM). Compounds **3e**, **3f**, and **3j** emerged as multipotent inhibitors with good to moderate inhibition potential against all of the tested targets. Based on these results, the two most potent inhibitors (**3e**, **3f**) against ChEs and MAO-B, respectively, were selected for further biological and *in-silico* analysis.

### SH-SY5Y Neuroblastoma Cell Toxicity

**Figure 2:**
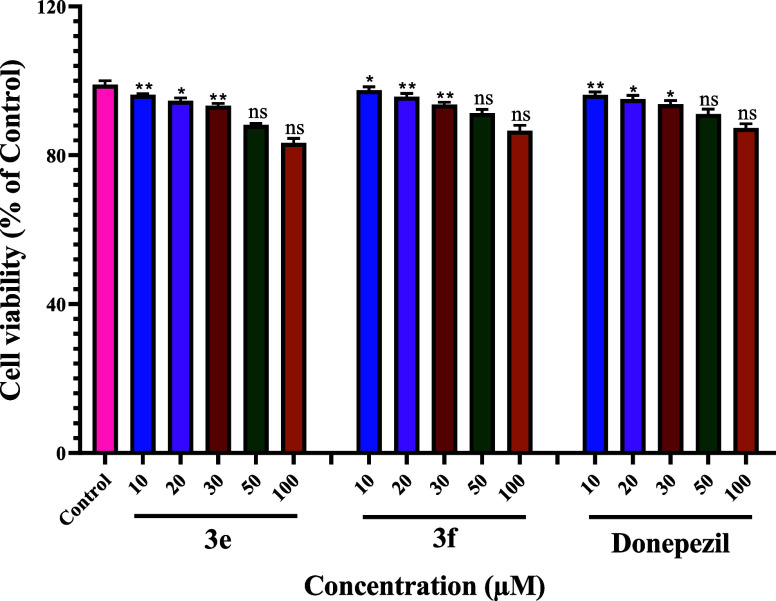
Cytoprotective and cytotoxic effects of compounds on SH-SY5Y cells. Cytotoxic activity after 48 h incubation with compounds **3e**, **3f**, and donepezil at different concentrations on SH-SY5Y cells. *P < 0.05, **P < 0.001, and ***P < 0.001; ns= nonsignificant.

There is a critical need to find drugs that are safe and have central nervous system (CNS) activity. To determine the biological safety of the synthesized compounds, we performed a cytotoxicity study using a human SH-SY5Y neuroblastoma cell line. Cytotoxic effects of selected compounds **3e**, **3f**, and donepezil were evaluated at different concentrations (10, 20, 30, 50, and 100 μM) in SH-SY5Y neuroblastoma cells. No cytotoxicity was observed at the tested concentrations of 10, 20, 30, and 50 μM. However, the selected compounds displayed negligible neurotoxicity at higher concentrations (100 μM) (Figure 2).

### Cell Viability Assay on Normal Human Embryonic HEK-293 Cells

**Figure 3:**
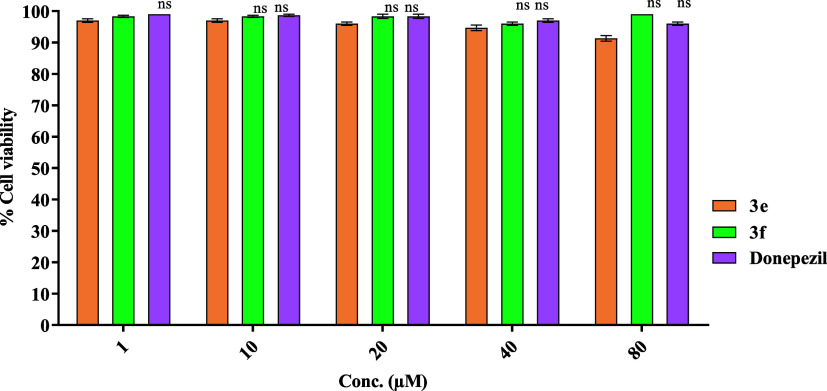
Cell Viability Assay results of compounds **3e** and **3f** on normal human embryonic HEK-293 cells at 1, 10, 20, 40, and 100 μM concentrations as obtained from MTT assays. Two-way ANOVA and the Bonferroni test were followed. Data were represented as mean ± S.E.M.; all of the values were not significant (ns) compared to that of the control group.

The cytotoxic potential of compounds **3e**, **3f**, and donepezil was evaluated on human embryonic cells (HEK-293) by an MTT assay. It was observed that normal cells were not affected by their exposure to the tested compounds, indicating their noncytotoxic profile for normal cells (noncancerous cells) (Figure 3).

### PAMPA BBB Assay

A fundamental requirement for the effectiveness of anti-AD drugs is their capability to traverse the BBB (blood–brain barrier). To assess the BBB permeability of the selected synthesized compounds (**3e**, **3f**), we conducted parallel artificial membrane permeation assays (PAMPA) following a previously reported procedure. We used diazepam and atenolol as BBB permeability standards. The tested compounds showed high permeability values **(3e**, **3f)**, which were comparable to those of the standard drugs donepezil and safinamide. Table 2 summarizes the BBB PAMPA results.

**Table 2:** PAMPA BBB Permeability Assay Results

compounds label	permeability (PAMPA-BBB)^a^P_e(tested)_ (10^–6^ cm/s)	prediction of CNS penetration
3e	18.73 ± 1.12	CNS+^b^
3f	18.26 ± 1.27	CNS+
donepezil	16.71 ± 1.01	CNS+
safinamide	7.56 ± 0.02	CNS+
validation of the model by two commercial drugs		
diazepam	15.30 ± 0.07	CNS+
atenolol	0.75 ± 0.02	CNS–c

a ± SEM represents mean values; *n* = 3 (three independent readings).

b “CNS+” (prediction of high BBB permeation); P_e_ (10^–6^ cm/s) > 4.39.

c “CNS–” (prediction of low BBB permeation); P_e_ (10^–6^ cm/s) < 1.

### MAO-B Inhibition Reversibility Result

**Figure 4:**
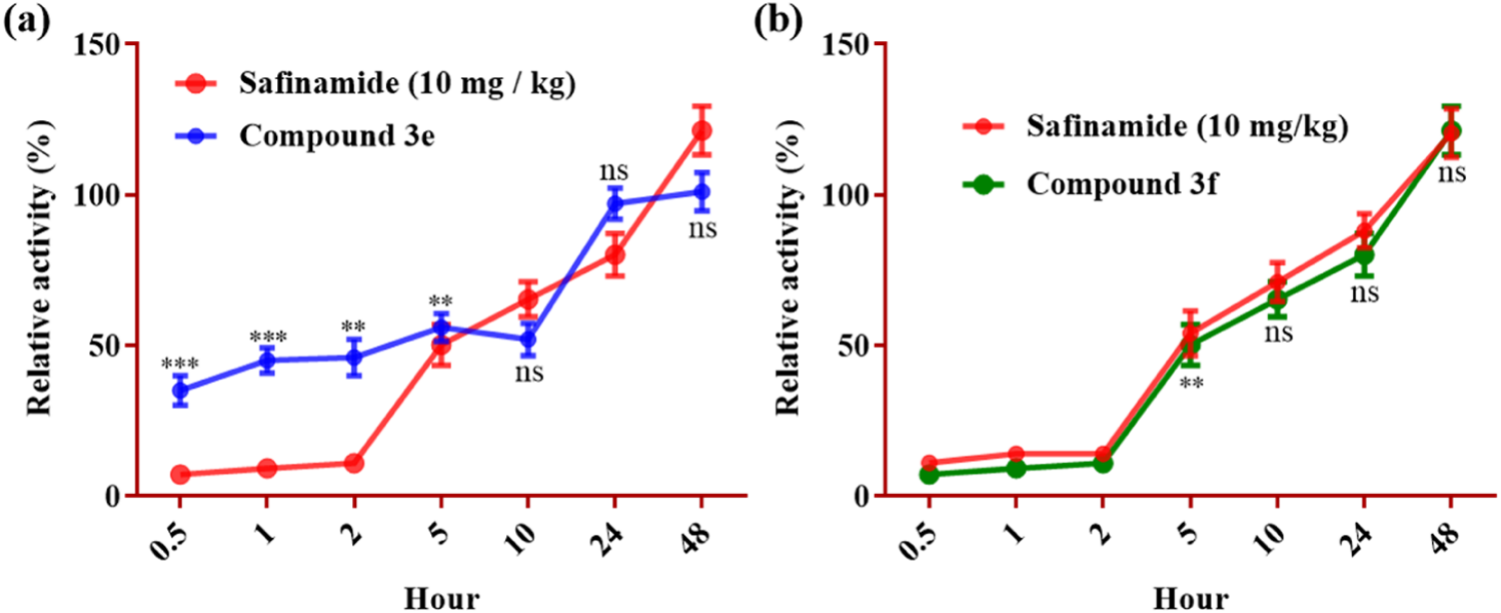
Time course of mice brain MAO-B inhibition *ex vivo* by a single dose of Compounds **3e** (a) and **3f** (b). Values are represented as mean ± SEM, analyzed by two-way ANOVA. *P < 0.05, **P < 0.001, and ***P < 0.001; ns= nonsignificant.

MAO-B inhibition reversibility of the most potent selected compounds (**3e**, **3f**) was studied by using an *ex vivo* MAO-B inhibition time course study. For **3e**, 50% and almost 95% activity of MAO-B was recovered after 5 and 24 h, respectively. Compound **3f** exhibited results similar to safinamide, showing almost 80% MAO-B activity recovered after 24 h (Figure 4).

## Molecular Docking

To determine the docking scores derived from different binding interactions, molecular docking studies were performed on control drugs (donepezil and safinamide) and the synthesized compounds **(3a–3m)**. The AChE docking scores for synthesized compounds ranged from −6.551 to −9.885 kcal/mol (the highest binding affinity shown by compound **3e**), as compared with −13.443 kcal/mol for donepezil. Compound **3e** also demonstrated a high docking score of −8.333 kcal/mol against BChE, which was close to donepezil (−9.199 kcal/mol). Docking scores for MAO-B ranged from −7.19 to −11.876 kcal/mol. The highest score shown by compound **3f** was very close to the safinamide docking score (−11.616 kcal/mol), which further supports *in vitro* studies. For detailed analysis, the docking scores for all compounds are provided in the Supporting Information.

**Figure 5:**
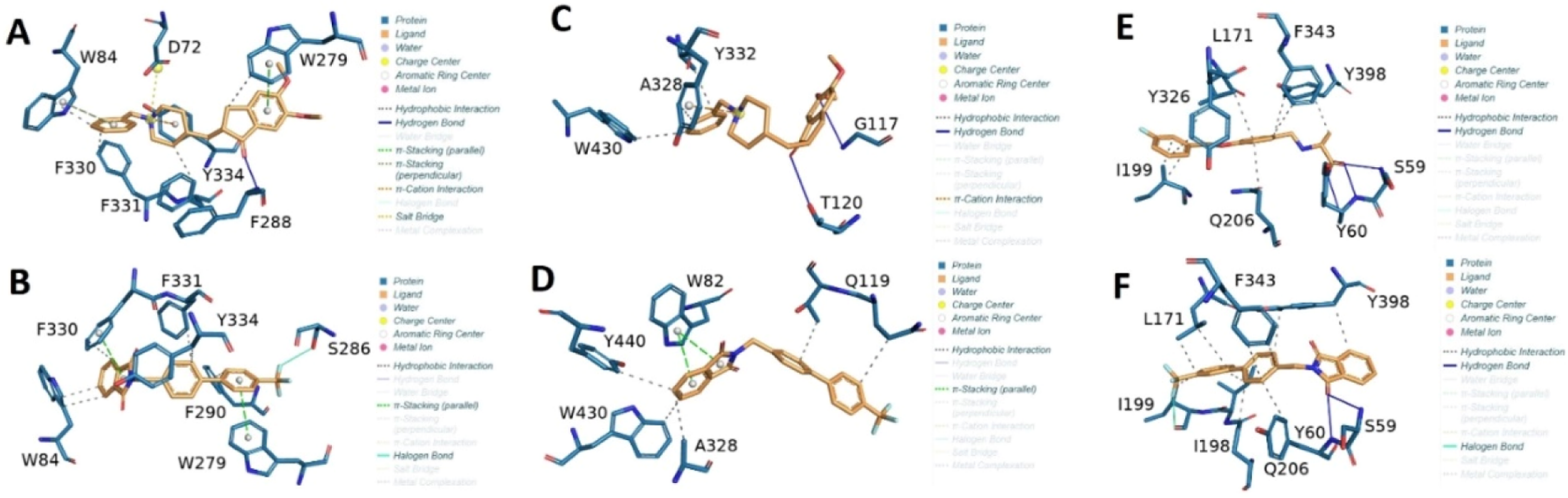
Interaction diagram of protein–ligand interaction. (A) AChE–donepezil (B) AChE–3e (C) BChE–donepezil (D) BChE–**3e** (E) MAO-B-Safinamide and (F) MAO-B-**3f**.

In optimal docking conformations for each compound, all ligands, alongside benchmark inhibitors, are located within the identical binding pocket of AChE, BChE, and MAO-B. The AChE–ligand complexes exhibit shared interacting residues, namely, D72, W84, W279, F330, F331, W279, I287, S286, F288, F290, and Y334 as compared to D72, W84, F330, F331, Y334, W279, and F288 for the AChE–donepezil complex (Figure 5A). Donepezil forms a critical hydrogen bond through the oxygen extending from its structure to the amino group of residues F288 situated in the Mid Gorge region of AChE besides hydrophobic interactions with aromatic residues such as W84, F330, W279, and Y334. The AChE–3e complex forms hydrophobic interactions with aromatic residues W84, F330, F331, W279, S286, F290, and Y334 (which cover 50% of the total surface area of the gorge region; Figure 5B). Furthermore, the parallel alignment of the W84 benzene ring to 3e benzene structure, along with the proximity of the W279 benzene ring to the benzene ring of 3e, facilitates strong π–π interactions, enhancing the binding specificity and stability within the AChE active site.

In BChE–ligand complexes, the residues surrounding the standard donepezil and test compounds are W82, G117, Q119, T120, A328, Y332, W430, and Y440. BChE–donepezil interaction involves residues G117, T120, A328, Y332, and W430; donepezil forms two hydrogen bonds with residues G117 and T120 (Figure 5C). In the case of BChE–**3e**, binding interactions involve residues W82, Q119, A328, W430, and Y440 (Figure 5D). Hydrophobic interactions with residues Q119, A328, W430, and Y440 form a snug pocket around the ligand, facilitating van der Waals interactions. The parallel alignment of the W82 benzene ring to the **3e** benzene structure facilitates strong π–π interactions, enhancing the binding specificity and stability within BChE’s active site.

The MAO-B-ligand complexes exhibit shared interacting residues, namely, S59, Y60, L171, I198, I199, Q206, Y326, F343, and Y398. In contrast, MAO-B-safinamide complex shows interactions with unique set of residues, including S59, Y60, L171, I199, Q206, Y326, F343, and Y398, as depicted in Figure 5E. Safinamide forms three hydrogen bonds through the oxygen extending from its structure to the amino group of residues S59 and Y60 situated in the active site region of MAO-B. Additionally, it shows hydrophobic interactions with aromatic residues, such as L171, I199, Q206, Y326, F343, and Y398. In contrast, the MAO-B-**3f** complex interacts with a distinct set of residues, including S59, Y60, L171, I198, I199, Q206, Y326, F343, and Y398 as illustrated in Figure 5F. Compound **3f** makes two crucial hydrogen bonds with S59 and Y60, the same as that of safinamide. Moreover, hydrophobic interactions with aromatic residues (L171, I198, I199, Q206, Y326, F343, and Y398), which cover 30% of the total surface area of the active site region, are observed. The I199 aliphatic chain makes a halogen bond with the **3f** halogen part, facilitating strong π–π interactions and enhancing the binding specificity and stability within the MAO-B active site.

### Structural Interaction Fingerprinting Analysis

Binding cavities of AChE–donepezil and AChE–**3e** share the same residues (D72, W84, F330, F331, Y334, W279, and F288) (Figure S27A,B) respectively. Residues D72, W84, G117, G118, Y121, S122, G123, Y130, Q199, W279, L282, S286, I287, F288, R289, F290, F330, F331, Y334, and G335 were investigated in fingerprinting analysis of AChE (Figure S27A). S286 and F288 showed H-bond acceptor interaction with all ligands, which can be seen in green (H-bond donor) (Figure S27A). Almost all ligands are involved in conserved interaction with W84, G117, G118, Y121, W279, L282, S286, I287, F288, R289, F290, F330, F331, and Y334, which are crucial residues in the binding cavity of AChE because they are essential for residual interaction. Like **3e**, most compounds have hydrogen bonding interaction with residues S286 and F288, shown by green color (donor H-bond), yellow color (hydrophobic interaction), and gray color (H-bond acceptor) in Figure S27A. All residues present in the binding cavity of AChE showed interaction in the fingerprinting analysis. Although residues G123, S122, L282, and G335 are not present in the binding cavities of AChE, they are involved in the side chain and backbone interaction with ligands **3e**, **3h**, **3j**, **3a**, and **3b**, as shown in sky blue color for side chain and orange color for backbone interaction in the table of fingerprinting.

Binding cavities of BChE–**3e** and BChE–donepezil share the same residues (W82, G117, Q119, T120, A328, Y332, W430, and Y440) as shown in Figure S27B. Residues N68, I69, D70, S72, G78, W82, G116, G117, Q119, T120, Q197, S198, W231, T284, P285, L286, S287, V288, N289, A328, F329, Y332, W430, M437, H438, and Y440 are present in fingerprinting analysis of BChE, as shown in Figure S27B. Ligands **3b**, **3g**, and **3j** have H-bond acceptor interactions with residue D70 (can be seen in pink color) (H-Acceptor) in the fingerprint analysis. Almost all ligands are involved in conserved interaction with D70, S72, G78, W82, G116, G117, P285, L286, S287, V288, N289, A328, F329, Y332, W430, M437, H438, and Y440, which are crucial residues in the binding cavity of BChE because they are essential for residual interaction. Like **3e**, most compounds have hydrophobic, backbone, and side chain interaction with residues D70, S72, G78, W82, G116, G117, P285, L286, S287, V288, N289, A328, F329, Y332, W430, M437, H438, and Y440; these are shown by green (donor H-bond), yellow (hydrophobic interaction), and sky blue color (side chain interaction) (Figure S27B). As mentioned earlier, all residues present in the binding cavity of BChE showed interaction in fingerprinting analysis. However, residues N68, I69, G78, M437, P285, and W231 are not present in the binding cavity of BChE but are involved in making hydrophobic, side chain, and backbone interactions with ligands **3m**, **3g**, **3k**, and **3d**.

Residues present in the binding cavity of MAO-B-safinamide and that of MAO-B-**3f** are the same (S59, Y60, L171, I198, I199, Q206, Y326, F343, and Y398). Residues R42, Y43, G57, G58, Y60, F168, L171, C172, I198, I199, G205, Q206, C296, I316, Y326, F343, C397, Y398, G434, Y435, and M436 are present in fingerprinting analysis of MAO-B (Figure S27C). Residue G206 forms hydrogen bond acceptor interaction with all ligands (shown in gray color) (H-Acceptor; in the fingerprint analysis (Figure S27C)). Almost all ligands are involved in conserved interaction with G58, Y60, F168, L171, C172, I198, I199, G205, Q206, C296, I316, Y326, F343, C397, 398, G434, Y435, and M436, which are important residues in the binding cavity of MAO-B because of their involvement in residual interaction. Further, most compounds have hydrophobic, backbone, and side-chain interactions with the same residues involved in conserved interaction shown by gray color (H-Acceptor), yellow color (hydrophobic interaction), and sky blue color (side-chain interaction). We found that all residues present in the binding cavity of MAO-B were involved in interaction with ligands in fingerprinting analysis. Residues R42, Y43, I316, F168, C296, and M436 are not present in the binding cavity of MAO-B but are involved in making hydrophobic, side chain, and backbone interactions with ligands **3l**, **3e**, **3g**, and **3d**, as shown in yellow for hydrophobic interactions and sky blue for side chain and backbone interactions and in orange in the table of fingerprinting.

### ADME Analysis

**Figure 6:**
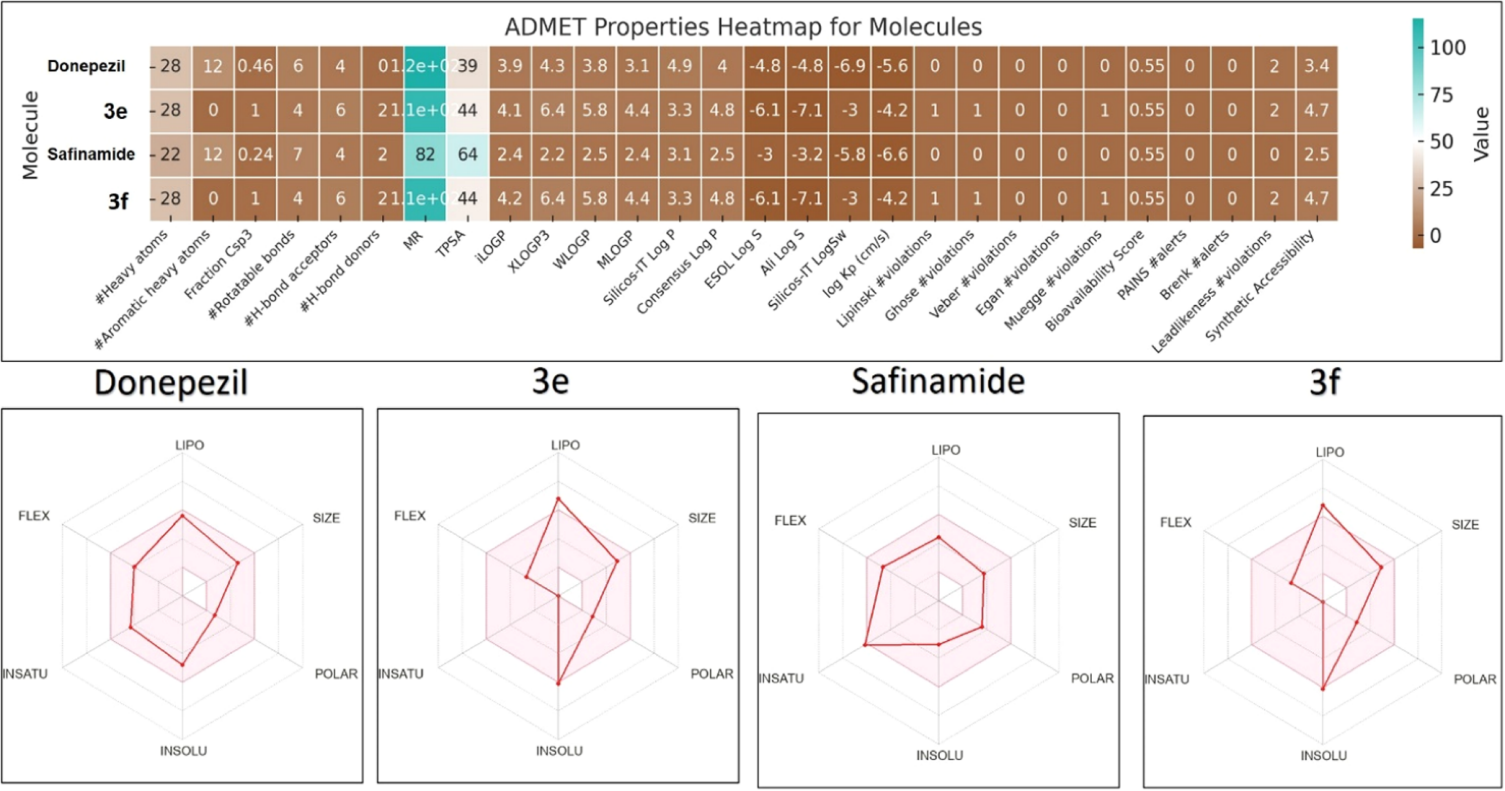
Heatmap and radar diagrams of SwissADME analyses of four selected compounds.

The ADMET analysis of compounds **3e**, **3f**, donepezil, and safinamide (Figure 6) showed that all four compounds have a moderate bioavailability score of 0.55, indicating a similar absorption potential. The synthesized compounds (**3e**, **3f**) exhibit higher lipophilicity, with XLOGP3 values of 4.1 and 4.2, respectively, compared with donepezil (3.9) and safinamide (2.4). This higher lipophilicity might be responsible for their BBB permeability in the PAMPA assay. However, these compounds show reduced solubility (ESOL Log S of −6.1 for both **3e** and **3f**), which could affect their absorption, distribution, balancing between CNS penetration, and adequate systemic bioavailability. On the other hand, donepezil and safinamide possessed moderate solubility (Log S of −4.8 and −3.2, respectively). None of the compounds violate Lipinski’s, Ghose’s, Veber’s, or Egan’s rules, affirming their drug-like properties and potential as therapeutic agents. Furthermore, the absence of PAINS and Brenk alerts suggests a low risk of off-target effects and toxicity. The synthetic accessibility scores ranging from 2.5 (safinamide) to 4.7 (**3e** and **3f**) indicated that these compounds are moderately easy to synthesize for further chemical optimization and large-scale production. Overall, these properties suggest that compounds **3e** and **3f** have favorable ADMET profiles with a balance between effective CNS penetration and manageable pharmacokinetic properties.

### DFT and MESP Studies

**Figure 7:**
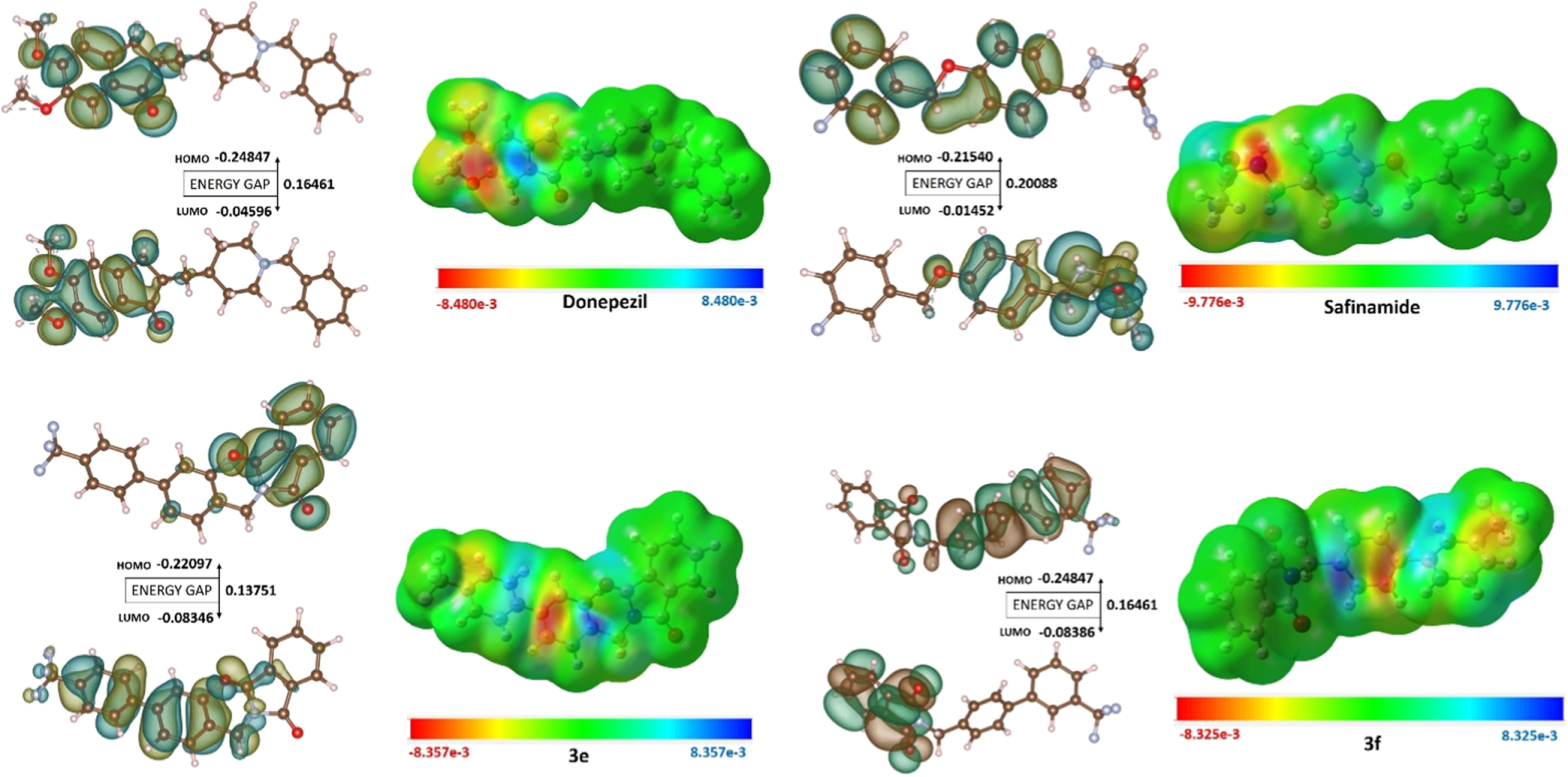
ESP structures (in solvent phases) formed by mapping of total density over electrostatic potential and optimized structures of donepezil, **3e**, safinamide, and **3f**. Calculated HOMO and LUMO of potent derivatives at the B3LYP/SVP level of DFT calculations for all selected ligands.

We utilized Molecular Electrostatic Potential (MESP) mappings for comparative analysis of the electronic properties of newly synthesized inhibitors (**3e** and **3f**) of acetylcholinesterase (AChE) butyrylcholinesterase (BChE) and monoamine oxide B (MAO-B) and the standard inhibitors of these enzymes (donepezil and safinamide). As illustrated in Figure 7, these mappings delineate the unique electronic characteristics that facilitate specific biochemical interactions with AChE, BChE, and MAO-B. The studies were executed in aqueous settings to evaluate the CNS-related neuroprotective functions of these compounds under physiological conditions, providing a pertinent context for analyzing these prospective therapeutics.

The MESP mappings underscore areas of pronounced electronegative potential, identified by a deep red hue across all four compounds. These crucial zones indicate preferred sites for the electrophilic attack, essential for robust molecular binding and inhibition of respective proteins, i.e., AChE, BChE, and MAO-B by top-scoring inhibitors **3e** and **3f** and the standard inhibitors. Detailed Mulliken population analysis reveals that the oxygen atom at position 27 in donepezil possesses an average Mulliken charge of −0.587918, marked as the most intensely red region on the MESP mapping.

An oxygen atom number 2 of the attached methoxy moiety linked at carbon 3 in the same benzene ring with an average Mulliken charge of −0.580041 enhances the electronegative properties of the corresponding ring, making it suitable for binding to the nearby residues. The other side of the benzene ring is illustrated as a blue area, indicating a highly electropositive region. Most of the area in the MESP images shown as the green region makes the rest of the molecule suitable for making hydrophobic interactions with protein. A similar pattern of the benzene ring is observed in the **3e** structure, and the central benzene ring shows a red area at the C2 and C3 positions, along with the other side being blue. The MESP of safinamide also presents highly negatively charged areas near oxygen at position 4 and nitrogen at position 6 with a Mulliken charge of −0.552844 and −0.585618, respectively, enhancing its hydrogen bonding potential with MAO-B. Notably, **3f** exhibits more neutral regions (indicated in green), implying a potential for hydrophobic or van der Waals (vdW) interactions, while other regions on **3f** also feature red areas at positions 1 and 2 and the area around three fluorides attached to carbon at position 25, supporting interactions between protein and ligand.

This pivotal analysis is essential for determining the reactivity of the compounds. The HOMO–LUMO energy gap, indicative of a molecule’s kinetic stability, elucidates the energy differential between the highest occupied and lowest unoccupied molecular orbitals, enabling enhanced energy transfer within the molecule. The molecular surface plots of the HOMO and LUMO for donepezil, **3e**, safinamide, and **3f** are presented in Figure 7. The electron-acceptor potential of an inhibitor is indicated by its LUMO value, while its HOMO value represents its electron-donating ability. Table 3 has a detailed summary of the computed quantum chemical descriptors for these compounds under aqueous conditions. DFT calculations have clarified crucial molecular characteristics of these inhibitors, unveiling their distinct electronic structures and reactivities.

**Table 3:** DFT Indices of the Selected Ligands along with Calculated Values of Electrophilicity Index and Electrochemical Potential

parameters for DFT analysis											
ligand	dipole moment (Debye)	HOMO (a.u.)	LUMO (a.u.)	energy gap (Δ*E*_Gap_)	ionization potential (eV)	electron affinity (eV)	electronegativity χ (eV)	electrochemical potential μ (eV)	hardness η (eV)	softness S (eV)	electrophilicity ω (eV)
donepezil	8.0095	–0.24847	–0.04596	0.16461	0.24847	0.04596	0.147215	–0.147215	0.082305	12.149930	0.131658
safinamide	3.1560	–0.21540	–0.01452	0.20088	0.21540	0.01452	0.114960	–0.114960	0.100440	9.956193	0.065790
**3e**	6.3425	–0.22097	–0.08346	0.13751	0.22097	0.08346	0.152215	–0.152215	0.068755	14.544397	0.168493
**3f**	5.4745	–0.24847	–0.08386	0.16461	0.24847	0.08386	0.166165	–0.166165	0.082305	12.149930	0.167735

Donepezil stands out with a notable dipole moment of 8.0095 D, positioning itself as a ligand with significant electron-donating capacity when compared to those of the other compounds studied. Safinamide, on the other hand, displays the lowest dipole moment of 3.1560 D, indicating a comparatively reduced interaction potential. Ligand **3e**, with a dipole moment of 6.3425 D, and **3f**, with 5.4745 D, offer intermediate electron-donating capabilities, indicating that they lie between donepezil and safinamide in terms of interaction potential with target proteins. The HOMO–LUMO energy gap analysis reveals that safinamide possesses the highest energy gap (0.20088 eV), indicating lower reactivity compared to donepezil, which has a slightly lower energy gap (0.16461 eV), and ligands **3e** (0.13751 eV) and **3f** (0.16461 eV), which display a moderate energy gap, suggestive of increased reactivity and potential biological efficacy.

Using Koopman’s theorem, global reactivity parameters for donepezil, safinamide, **3e**, and **3f** have been calculated, revealing distinct variations in their electronic properties. Donepezil is characterized by moderate electronegativity (χ = 0.147215 eV) and chemical potential (μ = −0.147215 eV), along with a relatively low hardness (η = 0.082305 eV). This lower hardness implies a softer electron cloud, as corroborated by its higher global softness (σ = 12.149930 eV∧-1) and moderate electrophilicity index (ω = 0.131658 eV). Safinamide exhibits the lowest electronegativity (χ = 0.114960 eV) and chemical potential (μ = −0.114960 eV), coupled with the highest hardness (η = 0.100440 eV) among the ligands. This indicates a stronger resistance to electronic reconfiguration, which is further supported by its lower global softness (σ = 9.956193 eV∧-1) and the lowest electrophilicity index (ω = 0.065790 eV), suggesting a reduced propensity to attract electrons. Ligand **3e** demonstrates a higher electronegativity (χ = 0.152215 eV) and chemical potential (μ = −0.152215 eV) than both donepezil and safinamide, with a hardness (η = 0.068755 eV) that reflects a moderate resistance to electronic deformation. Its global softness (σ = 14.544397 eV∧-1) indicates a considerable capacity for electron mobility, which is accompanied by a higher electrophilicity index (ω = 0.168493 eV), suggesting strong reactivity under electrophilic conditions. Ligand **3f**, with an electronegativity of 0.166165 eV and a chemical potential of −0.166165 eV, shows electronic properties similar to **3e** but slightly less hardness (η = 0.082305 eV) and comparable softness (σ = 12.149930 eV∧-1). Its electrophilicity index (ω = 0.167735 eV) is almost on par with that of **3e**, indicating similar reactivity levels.

These distinctive electronic parameters shed light on the varied reactivity profiles of these ligands, providing a critical understanding of their potential utility in different chemical and biological environments and guiding the future development of potent inhibitors with optimized therapeutic properties.

### MD Simulation

#### Structural Flexibility and Stability Analysis through Dynamic Simulations

Molecular dynamics (MD) simulations offer a complete computational perspective of molecular interactions at the atomic level, including protein dynamics, drug-target binding, and the complicated behavior of molecular components within ligand–protein complexes. MD simulations and free energy calculations were used to evaluate the interaction modes and binding processes of inhibitors with varying affinity for AChE and MAO-B.

**Figure 8:**
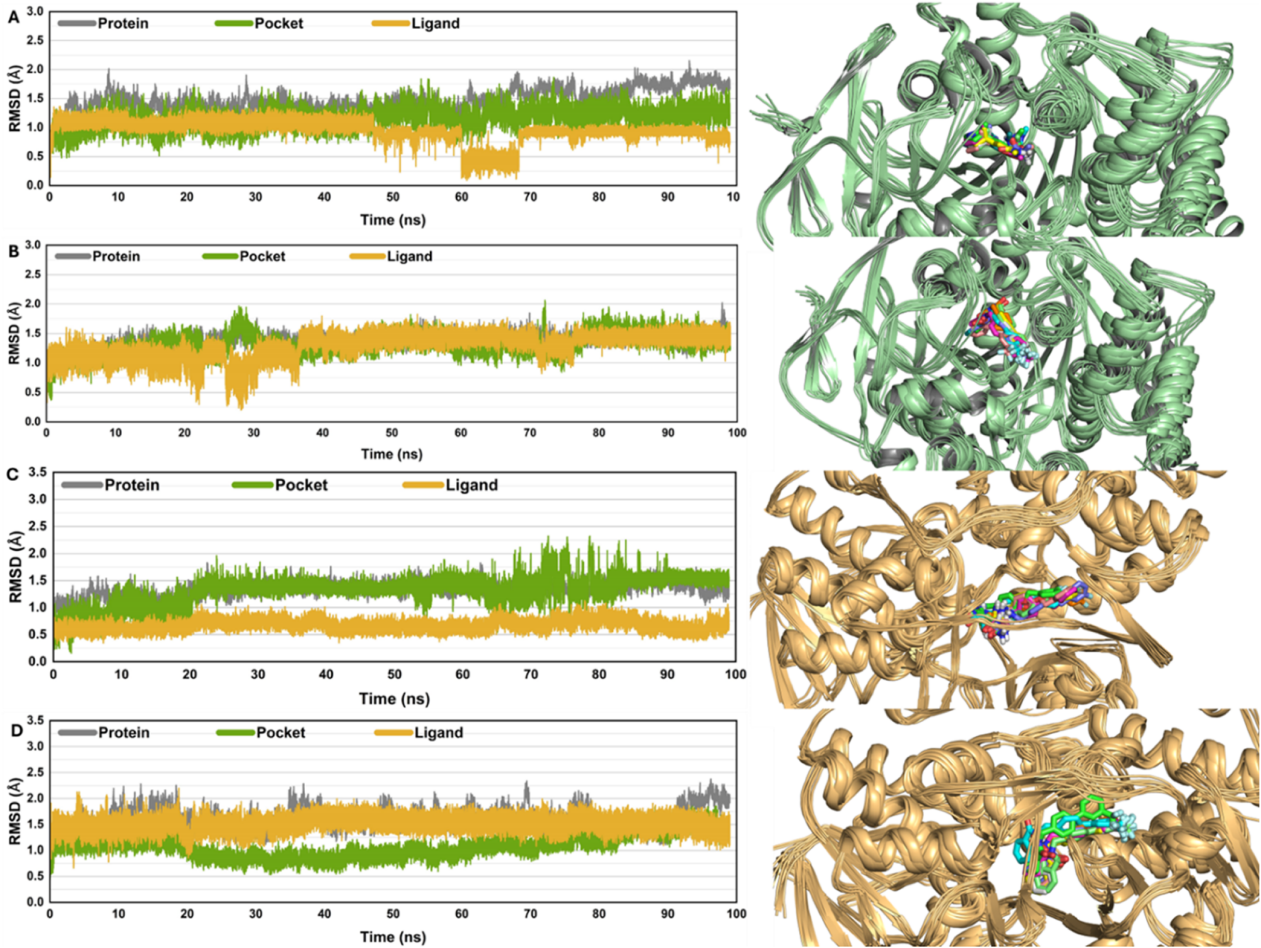
Comparative RMSD Analysis of various compounds over 100 ns of molecular dynamics simulations; (A) RMSD values of AChE–donepezil along with an MD-simulation snapshot showing superimposed fluctuations throughout 100 ns, (B) the AChE–3e complex along with a snapshot picture, (C) MAO-B-safinamide and its RMSD graph with a snapshot picture, and (D) the MAO-B-**3f** complex with its simulation graph and pyMOL figure.

To evaluate the dynamic stability of systems and confirm the sampling approach, their root-mean-square deviation (RMSD) with AChE was compared to the standard inhibitor donepezil across 100 ns of molecular dynamics (MD) simulations. The RMSD data showed that the systems under study, including AChE–donepezil, achieved equilibrium within the first five ns. After 30 ns of simulations, the AChE–donepezil systems exhibited constant variations, indicating an equilibrium condition. Figure 8A shows that the typical RMSD values for the protein’s Cα atoms were around ∼1.5 Å–2.0 Å, 0.5 Å–1.5 Å for the backbone atoms in the binding pocket, and ∼0.0 Å–1.0 Å for the heavy atoms of the ligands in the AChE–donepezil complex. Figure 8B illustrates the RMSD values for the most stable complex, AChE–3e, which exhibited the highest binding affinity toward AChE. Throughout the simulation, this complex remained in equilibrium, with RMSD values for the protein, binding pocket, and ligand consistently between ∼0.5 and 1.5 Å, indicating that the ligand maintained its binding interaction with AChE. For a more in-depth analysis, the stability of these conformations was further validated by superimposing the coordinates of representative MD-simulated snapshots onto their initial conformations, as shown on the right side of Figure 8A,B. This structural assessment confirmed that all complexes and ligands remained stable throughout the simulation, maintaining their original structure and essential interactions with neighboring atoms recorded at 10 ns intervals. These findings reinforce the reliability of the MD simulations.

Similarly, to examine the dynamic stability of the MAO-B complex with the standard drug safinamide, RMSD was calculated. Ligand RMSD values fluctuated by ∼0.5 Å throughout the simulations of 100 ns; the values for the binding pocket varied between ∼1.0 Å and 1.75 Å, whereas these were ∼1.0–1.5 Å for the protein (Figure 8C). RMSD values for the most stable complex of MAO-B-**3f** averaged around ∼1.5, ∼1.0, and ∼1.0 Å–1.5 Å for the Cα atoms of the protein, the backbone atoms of the binding pocket, and the heavy atoms of the ligands, respectively (Figure 8D). All of these fluctuations throughout 100 ns are presented along with their respective PyMOL images on the right panel of Figure 8C,D. This shows that the formed complexes remain within the binding cavity of the protein MAO-B.

The root mean square fluctuation (RMSF) values of key amino acid residues in the AChE–donepezil and AChE–3e complexes were analyzed to assess their flexibility. RMSF values indicate the degree of flexibility, with higher values representing increased flexibility and lower values suggesting reduced movement. The simulations of both AChE–donepezil and AChE–3e showed similar fluctuation patterns, suggesting that both compounds interact with the protein’s structural dynamics in comparable ways. Notable peaks in flexibility were observed around the same residues for both ligands, although the amplitudes varied.

**Figure 9:**
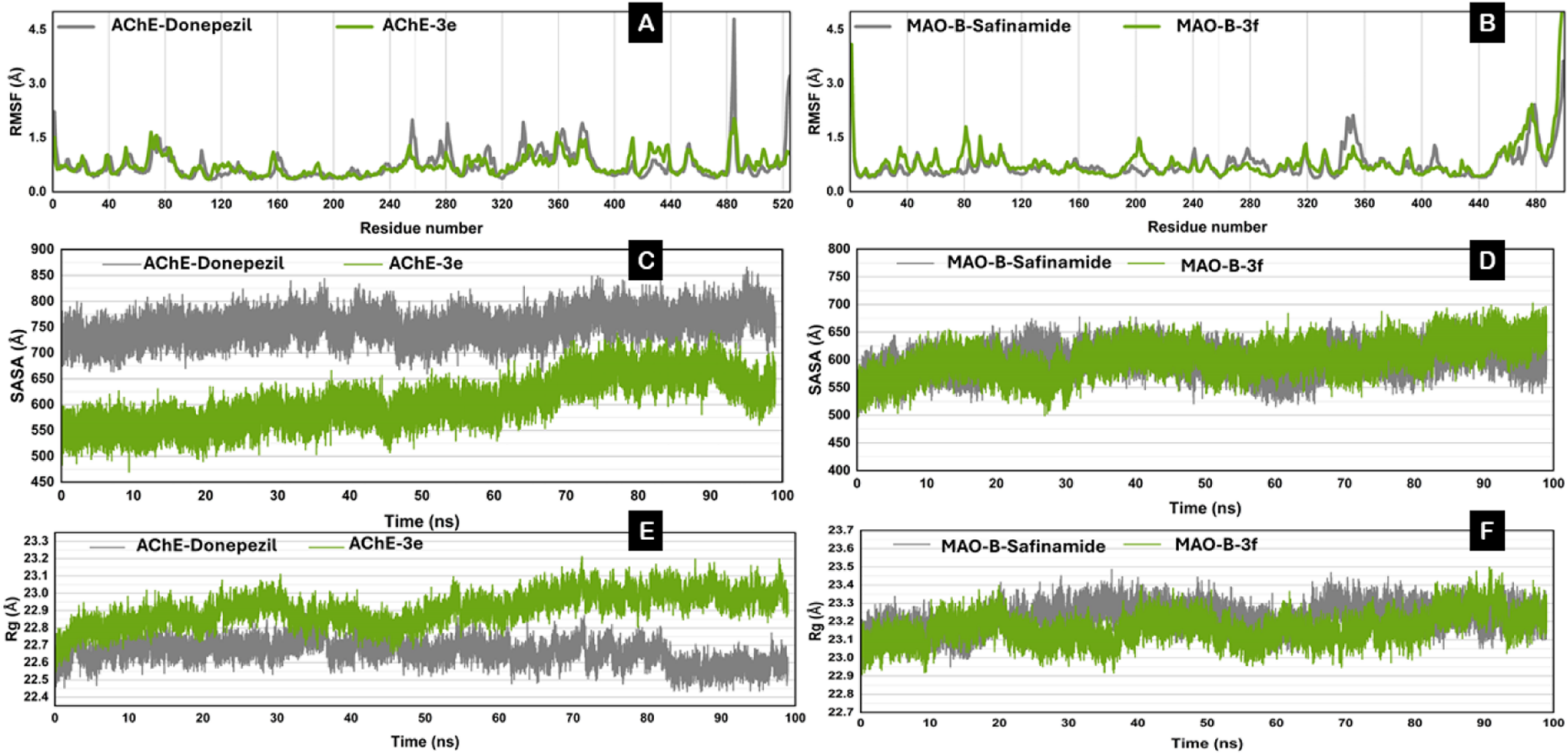
Comparative Analysis of RMSF, SASA, and Rg for AChE and MAO-B protein complexes with different ligands during molecular dynamics simulations. (A), (C), and (E) explain RMSF, SASA, and Rg graphs of AChE–donepezil and AChE–B, respectively, while (B), (D), and (F) explain RMASF, SASA, and Rg graphs of MAO-B-safinamide and MAO-B-**3f**, respectively.

Specifically, residues around 480 and 520 exhibited significant fluctuations in both complexes, with the AChE–donepezil complex showing higher fluctuations, 4.5 and 3.0 Å, compared to AChE–3e’s 1.5 Å in this region (Figure 9A).

This indicates that AChE–donepezil may induce greater flexibility in certain protein areas than that of AChE–**3e**. Between these peaks, the RMSF values were relatively low for both complexes, suggesting that these protein regions remained stable and rigid during the simulations. The differences in RMSF could reflect variations in binding affinities or stabilities within the enzyme–inhibitor complexes. The higher fluctuations in the AChE–donepezil complex may suggest a loose binding, allowing more movement or influencing a greater portion of the enzyme. Such changes in residue flexibility can impact the enzyme’s functionality, potentially affecting its catalytic efficiency. This analysis demonstrated that dynamic behavior and RMSF distributions across the protein structures in donepezil and 3e-ligand systems follow a similar pattern.

Similarly, the RMSF values of the MAO-B complex with the standard drug safinamide and its best merge complex **3f** are presented in Figure 9B. Both complexes have similar levels of flexibility, as shown by the similar fluctuation patterns across the residues, indicating that both compounds may interact in comparable ways with the protein’s structural dynamics. Although there were minor changes in the amplitudes of these peaks at certain identical residues, such as a larger amplitude at residue 200 in the 3f complex and a slightly higher amplitude at residue 360 in the safinamide complex, both compounds showed approximately similar flexibility.

The solvent-accessible surface area (SASA) was monitored over time to gain insight into the interactions between AChE and the inhibitors (Figure 9C). The AChE–donepezil complex showed an SASA value ranging from ∼700 to 800 Å, while the AChE–3e complex had an SASA value between ∼500 and 600 Å. This suggests that the AChE–donepezil complex has greater protein exposure to the solvent, indicating a potentially larger or more variable surface exposure. This could imply different conformational states or a more dynamic interaction with the solvent. Conversely, the AChE–3e complex’s smaller SASA points to a more compact protein shape or reduced solvent exposure, which may reflect tighter binding or less structural flexibility.

Similarly, SASA for MAO-B complexes with its ligands is shown in Figure 9D; interestingly, both MAO-B-safinamide and MAO-B-**3f** have values around ∼500 to 650 Å, showing similar and equal protein exposure to the solvent, indicating a potentially equal or more variable surface exposure.

To assess the effect of ligand binding on the protein’s structural compactness, the radius of gyration (Rg) over time was studied (Figure 9E). During the 100 ns simulation, the average Rg values for AChE complexed with donepezil and AChE–3e ranged from ∼22.5 to 22.7 Å and 22.6 to 23.0 Å, respectively. Lower Rg values signify a more compact and regular structure. The Rg values for both complexes remained nearly constant throughout the simulation, indicating that both ligands similarly affect the protein’s compactness and overall structure without significant differences. MAO-B-safinamide and MAO-B-**3f** have almost similar SASA values around ∼22.9 Å–23.4 Å, showing both complexes remained within similar fluctuations throughout the simulation (Figure 9F)

Figures 8A–C and9A–F, which outline RMSD, RMSF, radius of gyration, and SASA, shed light on the dynamic and structural characteristics of AChE during its interaction with donepezil and **3e**, as well as MAO-B’s interactions with safinamide and **3f** during molecular dynamics simulations. These analyses are crucial for understanding the protein’s function, stability, and drug-binding properties. Collectively, these metrics provide a comprehensive view of the protein’s behavior in solution, particularly its response to ligand interactions. By comparing the dynamic and structural impacts of all four ligands, we can pinpoint key structural elements that are essential for effectively inhibiting AChE and MAO-B. This insight is vital for designing potent inhibitors with significant therapeutic potential.

### Binding Free Energy Analysis

**Figure 10:**
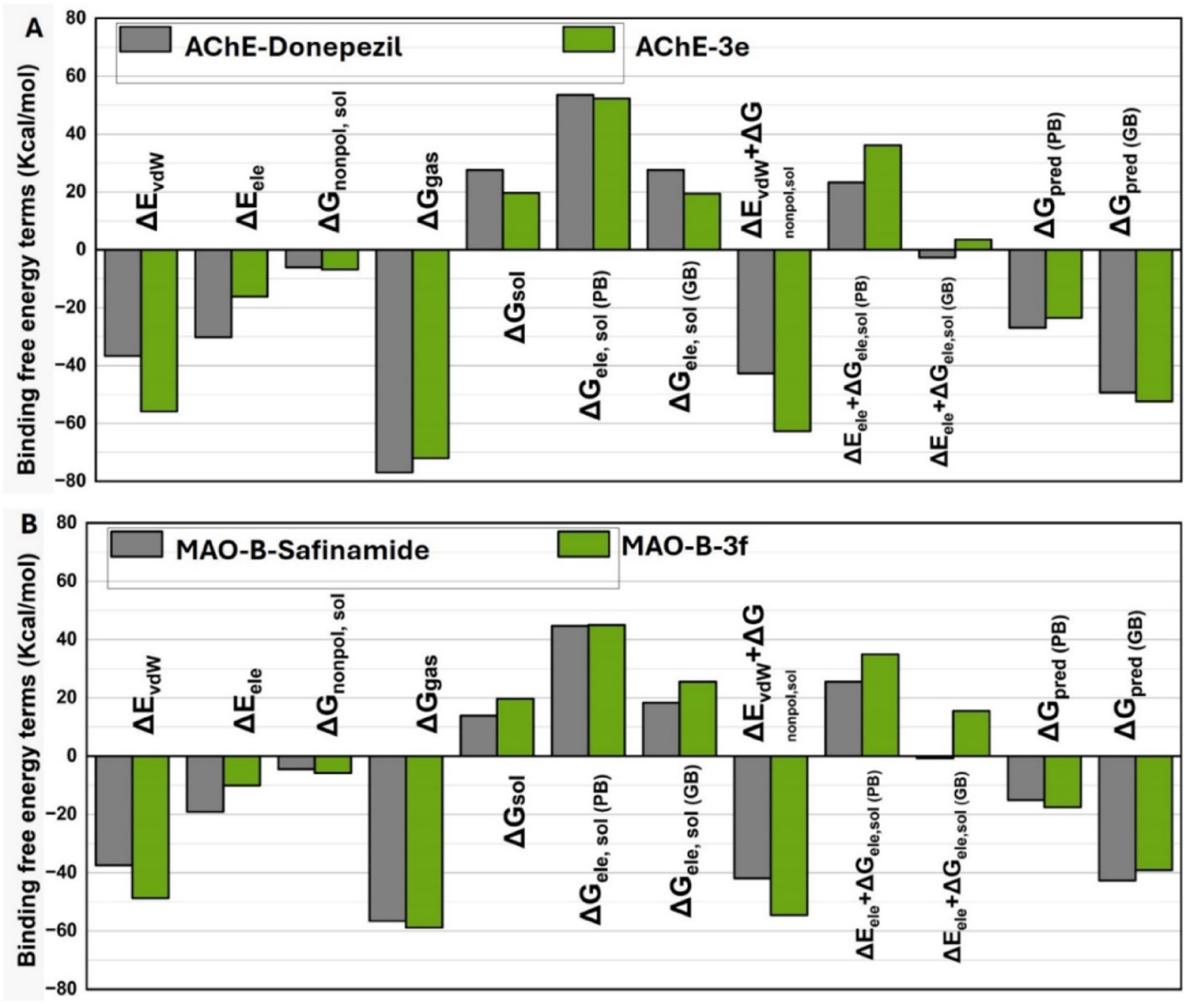
Binding free energy components of AChE and MAO-B complexes with different ligands: (A) represents the binding energy graph of AChE–donepezil and AChE–3e while (B) explains the binding energy graph of MAO-B-safinamide and MAO-B-**3f**.

Based on the system stability confirmed by RMSD fluctuations, 10,000 snapshots were randomly selected from the 1–100 ns interval of the MD simulation for binding free energy calculations. The binding affinities of the selected compounds toward AChE and MAO-B were determined using MM/PBSA and MM/GBSA methods.^28^Figure 10 illustrates the binding free energy components for the AChE–donepezil and AChE–3e complexes. The predicted binding affinities (Δ*G*pred (G.B.)) for AChE–donepezil and AChE–3e were −42.6988 and −32.6559 kcal/mol, respectively, which comply with the results of *in vitro* AChE inhibitory activity. Similarly, the predicted binding affinities (Δ*G*pred (P.B.)) also indicated that donepezil has a slightly lower affinity for AChE compared with **3e** (Figure 10A). The relatively small difference in MMGB/PBSA binding affinities suggests that AChE–donepezil might have a comparable inhibitory potential for AChE. These results support the consistency of the Δ*G*pred (GB/PB) values obtained from MM/GB/PB/SA methods with experimental data. Additionally, the MMGB/PBSA method’s ability to break down the total binding free energy into individual components offers a detailed understanding of the ligand–receptor binding dynamics. As shown in Figure 10A, the AChE–donepezil complex exhibits stronger van der Waals interactions compared to AChE–3e with Δ*E*vdw values of −48.7428 and −37.4592 kcal/mol, respectively. This indicates that AChE–donepezil forms tighter or more efficient packing with the protein.

Electrostatic energy (Δ*E*ele), which represents the electrostatic interactions between the charged groups of the protein and the ligand, values show that the standard inhibitor donepezil has a weaker electrostatic interaction with AChE (−19.1437 kcal/mol) than **3e** (−10.655 kcal/mol), suggesting less effective charge complementarity with the protein. The total gas-phase free energy (Δ*E*vdw + Δ*E*ele), which measures the free energy change during binding in the gas phase, is lower for AChE–donepezil (−41.9209 kcal/mol) than for AChE–3e (−54.5945 kcal/mol), indicating better binding affinity without solvent effects in the gas phase. Solvation-free energy is divided into polar (Δ*G*sol polar) and nonpolar (Δ*G*sol_nonpolar) contributions. Although both ligands show positive solvation energies, AChE–donepezil exhibits a less positive value (25.5362 kcal/mol) compared to AChE–3e (34.9214 kcal/mol), suggesting that it is less destabilized by solvation and may better adapt to a solvated environment. These results highlight that while electrostatic interactions play a role in the binding process, van der Waals and hydrophobic interactions are more critical in determining the inhibitory potential of AChE inhibitors. This analysis provides insight into the binding mechanisms and offers strategic guidance for designing more potent AChE inhibitors by enhancing van der Waals and hydrophobic interactions.

Similarly, Figure 10B shows the binding free energy components for the MAO-B-safinamide and MAO-B-**3f** complexes. The predicted binding affinities (Δ*G*pred (G.B.)) for MAO-B-safinamide and MAO-B-**3f**, at −49.354 and −52.3792 kcal/mol, respectively, closely align with the results of *in vitro* assays. Similarly, the predicted binding affinities (Δ*G*pred (P.B.)) also indicate that **3f** has a slightly higher affinity for MAO-B than safinamide (Figure 10B). However, the relatively small difference in the MMGB/PBSA binding affinities suggests that safinamide may have a comparable inhibitory potential for MAO-B as well. As shown in Figure 10B, MAO-B-safinamide exhibits stronger van der Waals interactions compared to MAO-B-**3f**, with Δ*E*vdw values of −55.8646 kcal/mol and −36.696 kcal/mol, respectively. This suggests that MAO-B-safinamide forms tighter or more effective packing with the protein. Electrostatic energy (Δ*E*ele), which reflects the interactions between the charged groups of the protein and ligand, shows that MAO-B-safinamide has a weaker electrostatic interaction (−30.2332 kcal/mol) compared to MAO-B-**3f** (−16.1988 kcal/mol), indicating less effective charge complementarity. The total gas-phase free energy (Δ*E*vdw + Δ*E*ele), representing the binding process’s free energy change in the gas phase, is lower for MAO-B-safinamide (−42.7622 kcal/mol) than for MAO-B-**3f** (∼-62.7117 kcal/mol), suggesting better binding affinity in the absence of solvent effects. When solvation-free energy is considered, which includes polar (Δ*G*sol_polar) and nonpolar (Δ*G*sol_nonpolar) contributions, both ligands show positive values. However, MAO-B-safinamide has less positive solvation energy (23.3185 kcal/mol) compared to MAO-B-**3f** (36.149 kcal/mol), indicating that MAO-B-safinamide may adapt better to a solvated environment.

## Conclusions

Phthalimide-based drugs have shown significant biological potential. We synthesized phthalimido-based biaryl derivatives through Suzuki coupling with a broad substitution pattern. All of the synthesized compounds were subjected to biological evaluation against ChEs and MAO isoforms. Compound **3e** displayed good enzyme inhibition activity against AChE (IC_50_ = 0.24 μM), BChE (IC_50_ = 6.29 μM), and MAO-B (IC_50_ = 0.11 μM). Compound **3f** exhibited the highest inhibition potency against MAO-B (IC_50_ = 0.091 μM), which is 124-fold greater than that of MAO-A. The selected compounds displayed negligible neurotoxicity at higher concentrations against the neuroblastoma cell line (SH-SY5Y). Also, the compounds were found to be nontoxic to normal human embryonic HEK-293 cells. Docking studies carried out on the 3D crystallographic structures of AChE, BChE, and MAO-B showed significant hydrophobic and hydrophilic interactions with amino acid residues. Moreover, ADME analysis and molecular dynamics simulations demonstrated promising binding affinities, favorable pharmacokinetic profiles, and dynamic stability of the compounds. Contrary to the traditional approach of developing a single-target compound with high potency, current researchers are focusing on identifying compounds with balanced biological actions against multiple targets. Compounds **3e**, **3f**, and **3j** emerged as multipotent inhibitors with good to moderate inhibition potential against all of the tested targets.

## Experimental Section

### Synthesis

The detailed stepwise procedure for the synthesis of all compounds and their analytical data are given in the ESI.

### Biological Evaluation

#### Cholinesterases Inhibition Assay

The cholinesterase *in vitro* bioassay for the synthesized compounds was performed using already reported protocols.^29,30^ Ellman’s assay method was used to study the inhibition of *Electrophorus electricus* AChE and Equine Serum lyophilized BChE. Stock solutions of synthesized compounds were prepared in potassium phosphate buffer (pH 8.0). A sufficient amount of 5,5-dithio-bis-2-nitrobenzoic acid (DTNB, Ellman’s reagent), a stock solution of synthesized compounds, and 0.03 U/ml of the enzyme (AChE or BChE) were added to a 96-well plate and incubated at 30 °C for 10 min. Subsequently, 1 mM ATCI or BTCI was added, and the reaction mixture was incubated for 15 min. Triplicates of reading were taken at 412 nm. Sample concentration was plotted against inhibitory activity to obtain IC_50_ values.

#### MAO Isoform Inhibition Assays

Inhibitory activity of the synthesized compounds against MAO isoforms, specifically human MAO-A and MAO-B, was determined by using benzylamine and tyramine substrates in the dark (protected from light) in a 96-well plate by an already reported fluorometric method.^31,32^

#### SH-SY5Y Neuroblastoma Cell Toxicity

The MTT assay was employed for assessing the effect of selected potent synthesized compounds on neuroblastoma cells (SH-SY5Y) purchased from ATCC CRL-2266.^29^ SH-SY5Y cells were cultured in 96-well plates (1 × 10^5^ cells/well) in a medium containing fetal bovine serum (FBS), penicillin (100 U/ml), and streptomycin (100 U/ml) at 37 °C and 5% CO_2_. After 1 day, the selected compounds (10, 20, 30, 50, and 100 μM) were added to SH-SY5Y cells and then incubated for 24 h. Subsequently, synthesized compounds were added to cells, followed by the addition of 20 μL of MTT reagent (10 mg/mL) at the specified time. Plates were then incubated at 37 °C and 5% CO_2_, and purple color precipitate formation was observed by a microscope. Lastly, DMSO (1 mL) was added to solubilize precipitates, and the plates were read at 570 nm to determine the cell viability. Results are expressed as percentage growth of cells, in which their viability is compared to control cells in the presence or absence of synthesized compounds and a standard. The following formula was utilized for the measurement of the % cell viability.
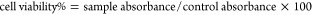


#### Cell Viability Assay on Normal Human Embryonic HEK-293 Cells

The MTT assay was employed for assessing the effect of selected potent synthesized compounds on the normal human embryonic HEK-293 cells by using the reported method.^33^

#### PAMPA BBB Assay

A model of PAMPA BBB *in vitro* assay was employed to determine the BBB penetration of the synthesized compounds.^30^ Briefly, synthesized compounds were first dissolved in DMSO (10 mM) that was further diluted by the addition of phosphate buffer (100 M) having a pH of 7.4. PBL dissolved in dodecane was used for coating the membranes of the donor plates. Then, the test compound (0.2 mL) was added to the plate, followed by PBS (0.2 mL; pH = 7.4) to the acceptor plate, which was further placed under the donor plate in order to form a “sandwich”. The plate was observed at room temperature for 20 h. A UV plate reader was employed for the measurements of the drug concentration in donor, acceptor, and reference wells. The permeability (Pe) of the compounds was measured by using the following formula.
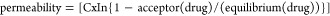


#### *Ex vivo* MAO Reversibility Assay

The *in vivo* study was approved by the Ethics Committee (No. EC/2093/2024). After the experiments, the animals were euthanized following Animal Euthanasia (AVMA Guidelines) guidelines to ensure humane treatment. Five groups of ICR mice were administered test samples by intraperitoneal injection, and the intact brains were excised and were then frozen for almost 1 h (−20 °C). Brain tissue (700 to 1000 μg) was suspended in sucrose (0.3 M) using a homogenizer (Ultra-Turrax T8; at 60 Hz for almost 70 s) and then subjected to centrifugation for 10 min at 1500 rpm (4 °C). 200 μL of supernatant was collected and centrifuged for 20 min at 15,000 rpm and 4 °C. Subsequently, the formed pellet was resuspended in KHPO_4_ solution (pH 7.2, 600 μL) and then subjected to 20 min centrifugation at 15,000 rpm and 4 °C. This process was repeated three times. Enzyme activity was determined using an Assay Kit (monoamine oxidase). The percentage inhibition of MAO-B was determined relative to the vehicle group (set at 100%), with adjustments made based on protein quantification. To examine the time course of MAO-B inhibition in the mouse brain, ICR mice were intraperitoneally (ip) administered the selected test samples and safinamide. The mice were then sacrificed at intervals ranging from 0.5 to 48 h.^34^

### In-Silico Evaluation

#### Docking Methodology

Molecular docking was applied to correlate the *in vitro* studies using the crystal structure of AChE in complex with donepezil (PDB: 4EY7) and MAO-B with safinamide (2 V5Z).^33,35^ The protein structure was prepared by removing water molecules and reconstructing any missing segments using the Prime tool. Water molecules within the active site but distant from the ligand were also removed, and the structure was subsequently optimized. Ligand structures were generated using LigPrep and were refined using the OPLS4 force field.^36^ A grid was established around the cocrystallized ligand using the Glide grid module. This approach enables precise ligand placement and interaction assessment, which are fundamental to structure-based drug design. Previous studies have also employed grid-based methodologies to investigate molecular interactions and optimize ligand binding, following a well-established methodology. Such approaches have provided valuable insights into favorable interaction regions within the active sites of AChE and MAO-B, aiding in the rational design of dual-target inhibitors.^37^ The Glide tool, part of Schrodinger Suites 2020_3 × 64, was employed to dock ligands into the binding site of 4EY7 and 2 V5Z, utilizing the Glide XP mode to evaluate docking accuracy. Results were analyzed using Schrodinger’s Maestro interface.^38^

#### Structural Interaction Fingerprinting (SIFt) Analysis

SIFt analysis was carried out to pinpoint the key residues involved in ligand binding. It categorizes interactions based on hydrophobic, H-bond donor, and H-bond acceptor properties. Interaction fingerprints for the protein–ligand complexes were generated using the SIFt module within Schrodinger_Suites_2020_3 × 64.^39^ The resulting fingerprints were displayed in Excel sheets, and the interaction type was color-coded, with ″1″ and ″0″ indicating the presence or absence of interactions, respectively.

#### ADME Analysis

The ADMET properties of compounds **3e**, **3f**, donepezil, and safinamide were assessed using the SwissADME web tool (http://www.swissadme.ch/). Each compound’s SMILES notation was entered into the SwissADME platform, and data was visualized in a heatmap and radar plot format.

#### DFT Studies/MESP/HOMO/LUMO Analysis

Density functional theory (DFT) calculations were systematically performed using the Gaussian 09 software package (Revision E.01). The B3LYP functional, in combination with the SVP basis set, was consistently applied. Optimized geometric configurations, frontier molecular orbital (FMO) energies, global and local reactivity indices, and molecular electrostatic potentials (MEPs) were evaluated. The checkpoint files generated during the simulations were analyzed by using Gauss View 6.

#### Molecular Dynamics Simulations

Molecular dynamics simulations using AMBER20 with the ff99SB force field were performed to further refine and stabilize the docking complexes of potent AChE (3e) and MAO-B (3f) inhibitors and the standards donepezil and safinamide.^40^ Binding affinities were determined by employing the MM/PB(GB)SA approach.^41^ All MD simulations, along with the molecular mechanics-based free energy estimations (MM/PB(GB)SA), were executed exclusively within the AMBER16 software environment,^42^ according to the previously established protocols and parameters.^43^

## Supporting Information Available

Analytical data for the synthesized derivatives, including ^1^H and ^13^C NMR, GC-MS, and FTIR spectra (PDF)
