# Supplementary material for: Synthesis, Pharmacological Evaluation, and Molecular Modeling of Phthalimide Derivatives as Monoamine Oxidase and Cholinesterase Dual Inhibitors
Source: ACS Omega. 2025 Mar 4;10(10):10385–400. doi: 10.1021/acsomega.4c10510 (PMC11923636; doi:10.1021/acsomega.4c10510)
Supplement: Supplementary file 1 — ao4c10510_si_001.pdf [file ao4c10510_si_001.pdf]

Supporting Information

Synthesis, Pharmacological Evaluation and Molecular Modelling of Phthalimide Derivatives as Monoamine-Oxidase and Cholinesterase Dual Inhibitors

Nabiha Abdullah,[a,b] Fahad Hussain,[c] Naseem Ullah,[a] Humaira Fatima,[a] Muhammad Afaq Tahir,[d] Umer Rashid,*[c] and Abbas Hassan*[b,e]

[a]Department of Pharmacy, Quaid-i-Azam University, Islamabad 45320 Pakistan.

[b]Department of Chemistry, Quaid-i-Azam University, Islamabad 45320 Pakistan.

[c]Department of Chemistry, Comsats University Islamabad, Abbottabad Campus Pakistan.

[d]Institute of Pharmaceutical Sciences, University of Veterinary and Animal Sciences, Lahore 54000 Pakistan.

[e]Department of Chemistry, College of Science, United Arab Emirates University, Al Ain, Abu Dhabi 15551, United Arab Emirates.

Contents

General Considerations	S3
General Procedure for Suzuki coupling of compounds 3a-3m	S4
Analytical Data of compounds 3a-3m	S5
Structural fingerprinting analysis of docked compounds (3a-3m)	S44
Molecular Docking scores of compounds 3a-3m	S45

General Considerations

Chemicals were purchased from Merck and Alfa Aesar chemical companies. 2-(4-(4,4,5,5-Tetramethyl-1,3,2-dioxaborolan-2-yl)benzyl)isoindoline-1,3-dione (CAS Number: 138500-87-5) was purchased from Merck. All the solvents were dried and distilled before setting up each reaction. The progress of the reactions was monitored by Thin-layer chromatography (TLC) using the pre-coated silica gel-60 F_{254} having 0.2 nm thickness. TLC plates were purchased from Merck (Germany). UV visible compounds were inspected under UV light at 254 nm. For visualization of UV inactive spots, different staining reagents such as potassium permanganate, anisaldehyde or ninhydrin solutions were used as required. Purification of compounds was achieved by flash column chromatography using silica gel of mesh size 200-300 as stationary phase. IR spectra were recorded on Schimadzu Fourier Transform Infra-Red Spectrophotometer model 270 using ATR (Attenuated total reflectance) instrument. Melting points were determined by using Gallenkamp melting point apparatus. GC-MS of the volatile compounds was performed using Agilent Technologies instrument, model 5975 MS with 6890 GC, with column specification of DB-5MS 30 m, 0.25 mm, 0.25 μm. The method utilized for GC-MS was at a temperature of 120-280 °C with a ramp of 10 °C/min, a flow rate of 1.5 ml/min, an injection volume of 5 μL and the inlet temperature was set at 250 °C. NMR spectroscopy was carried out using Bruker 300 NMR MHz spectrometer in deuterated solvents using TMS as an internal reference, at 300 MHz (^{1}H NMR) and 75 MHz (^{13}C NMR). Chemical shifts are mentioned in delta (δ) units while coupling constants (*J*) values are in Hertz unit (Hz).

General procedure for Suzuki coupling of 3a-3m

Aryl halide (2a-2m) (0.3 mmol, 100 mol%), boronic acid pinacol ester (1) (0.36 mmol, 120 mol%), Pd(dppf)Cl_2 (11.0 mg, 0.015 mmol, 5 mol%) and K_2CO_3 (82.9 mg, 0.6 mmol, 200 mol%) were weighed into an oven-dried 100 x 13 mm glass tube equipped with a magnetic stir bar. THF (0.6 mL, 0.5 M) was added, and the tube was purged with nitrogen gas and sealed with a screw cap. The reaction mixture was stirred vigorously at 80 °C for 24 h. The progress of the reaction was monitored by thin layer chromatography (TLC). After completion, the reaction mixture was cooled to room temperature. The product was purified by flash column chromatography using ethyl acetate and hexane as mobile phase.

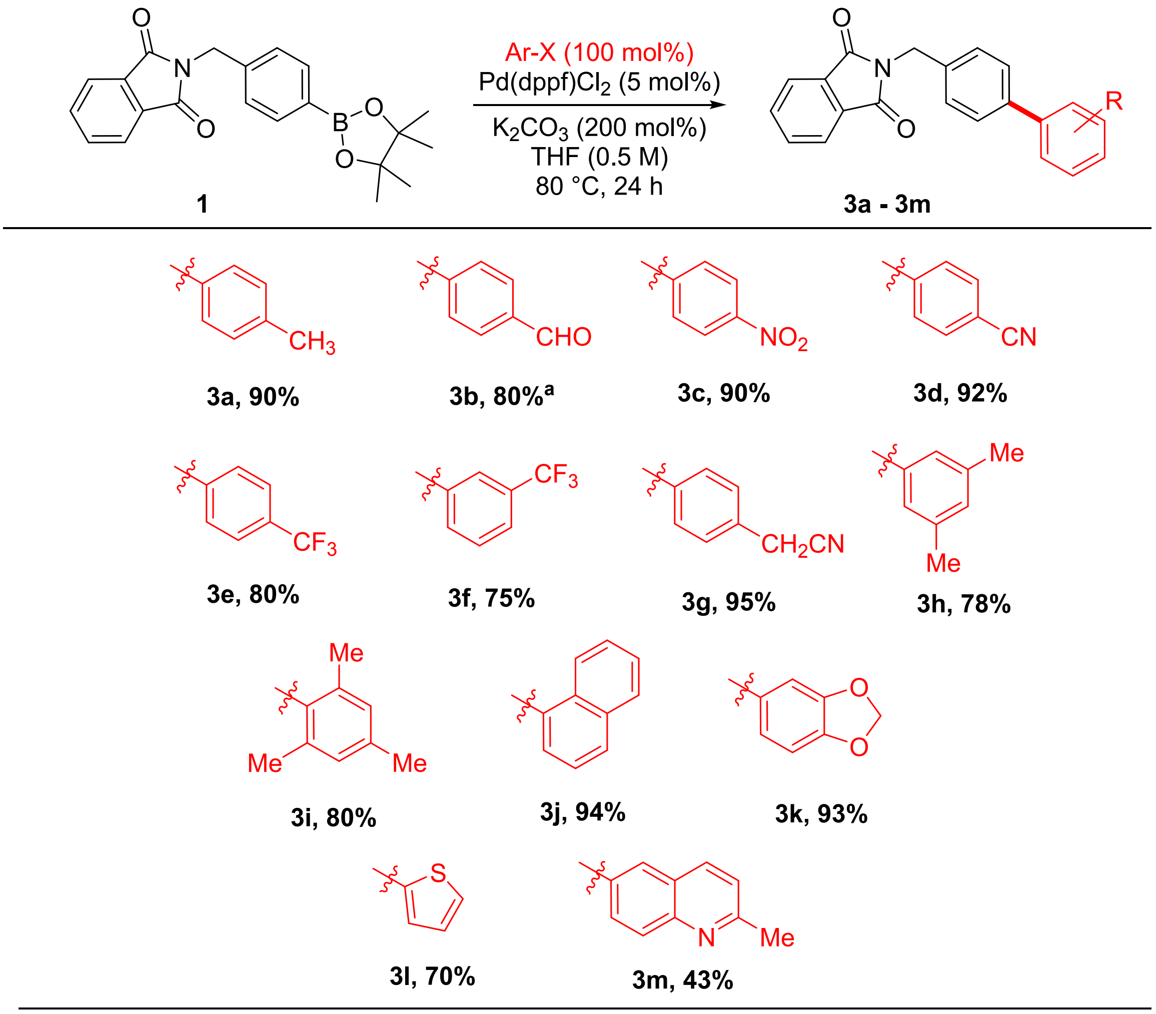

Scheme S1. Synthesis of *N*-benzyl substituted Phthalimide derivatives. [a]Reaction time was 4 h

Analytical data of compounds 3a-3m:

2-((4'-Methyl-[1,1'-biphenyl]-4-yl)methyl)isoindoline-1,3-dione (3a)

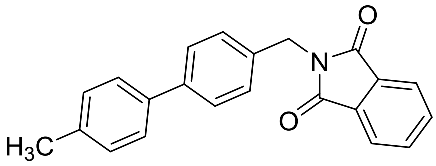

White solid, Yield = 90%, m.p. = 152-154 °C, R_f = 0.3 (1: 9 :: EtOAc: *n*-Hexane).

^{1}H NMR ($CDCl_3$, 300 MHz) $\delta_{(ppm)}$ = 7.83 (dd, *J* = 5.4, 3.3 Hz, 2H), 7.68 (dd, *J* = 5.4, 3.0 Hz, 2H), 7.53-7.41 (m, 6H), 7.24-7.19 (m, 2H), 4.86 (s, 2H), 2.36 (s, 3H).

^{13}C NMR ($CDCl_3$, 75 MHz) $\delta_{(ppm)}$ = 168.0, 140.7, 137.7, 137.1, 135.0, 134.0, 132.1, 129.4, 129.0, 127.2, 126.9, 123.3, 41.3, 21.1.

GC-EIMS (m/z): 327 (100%), 204, 179, 162, 130, 105, 76, 50.

HRMS-ESI (m/z): $[M+H]^+$ calc'd for $C_{22}H_{18}NO_2^+$, 328.1332; found, 328.1334.

FTIR (neat): $\bar{\upsilon}$ (cm^{-1}) = 3028, 2981, 1709, 1615, 1327, 1113, 1066, 939, 718, 530.

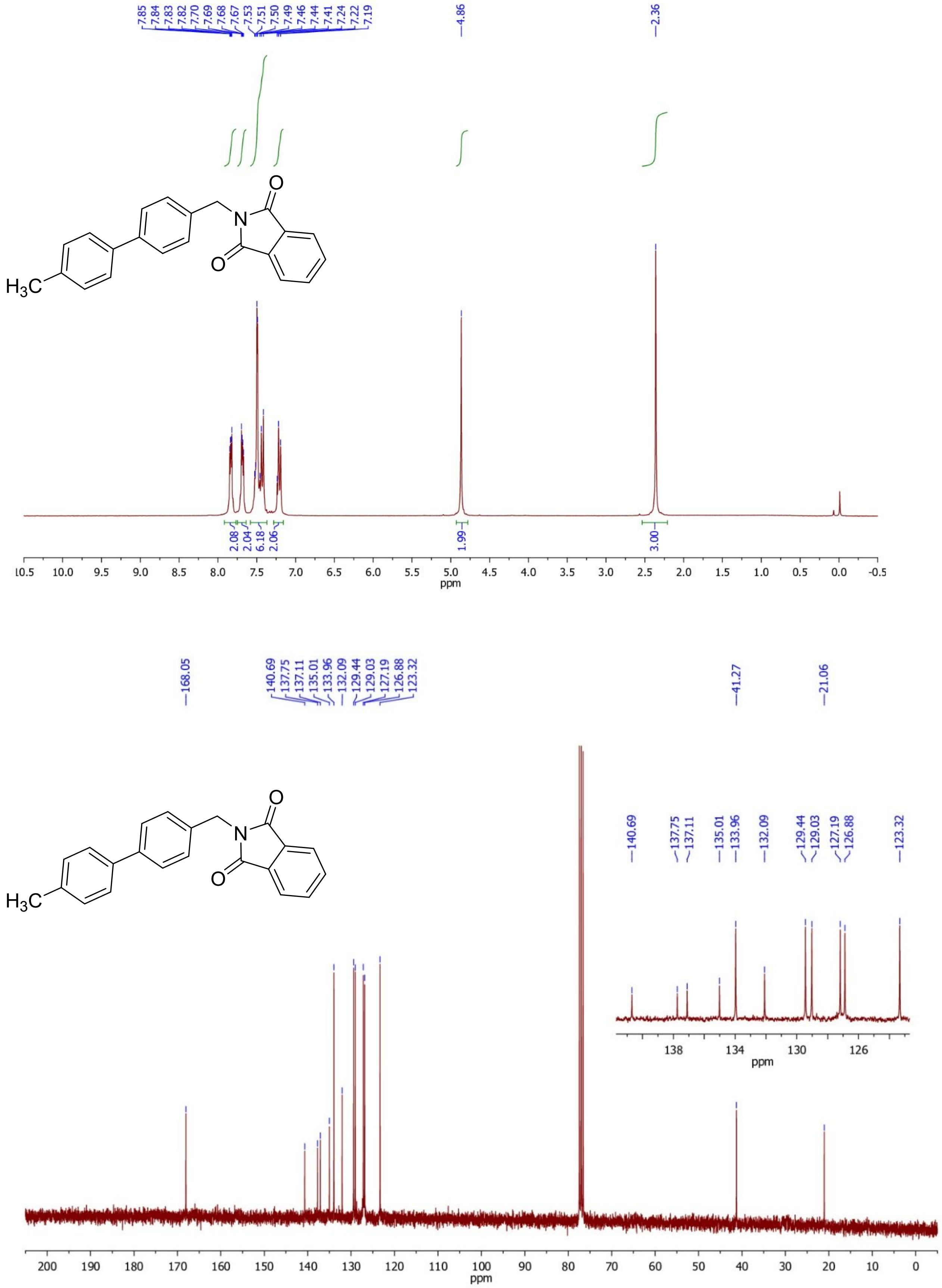

Figure S1. ^{1}H and ^{13}C NMR spectra of compound 3a.

4'-((1,3-Dioxoisoindolin-2-yl)methyl)-[1,1'-biphenyl]-4-carbaldehyde (3b)

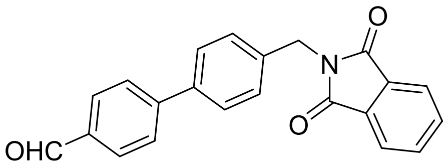

White solid, Yield = 80%, m.p. = 151-153 °C, R_f = 0.46 (1: 4 :: EtOAc: *n*-Hexane).

^{1}H NMR ($CDCl_3$, 300 MHz) $\delta_{(ppm)}$ = 10.04 (s, 1H), 7.92 (d, *J* = 9.0 Hz, 2H), 7.87-7.83 (m, 2H), 7.75-7.69 (m, 4H), 7.56 (q, *J* = 8.1 Hz, 4H), 4.90 (s, 2H).

^{13}C NMR ($CDCl_3$, 75 MHz) $\delta_{(ppm)}$ = 191.5, 167.6, 146.2, 138.8, 136.2, 134.7, 133.6, 131.6, 129.8, 128.8, 127.2, 127.2, 123.0, 40.8.

GC-EIMS (m/z): 341 (100%), 312, 281, 209, 181, 152, 130, 105, 77, 50.

HRMS-ESI (m/z): $[M+H]^+$ calc'd for $C_{22}H_{16}NO_3^+$, 342.1125; found, 342.1129.

FTIR (neat): $\bar{\upsilon}$ (cm^{-1}) = 3068, 2920, 2847, 1689, 1608, 1387, 1086, 932, 705, 530.

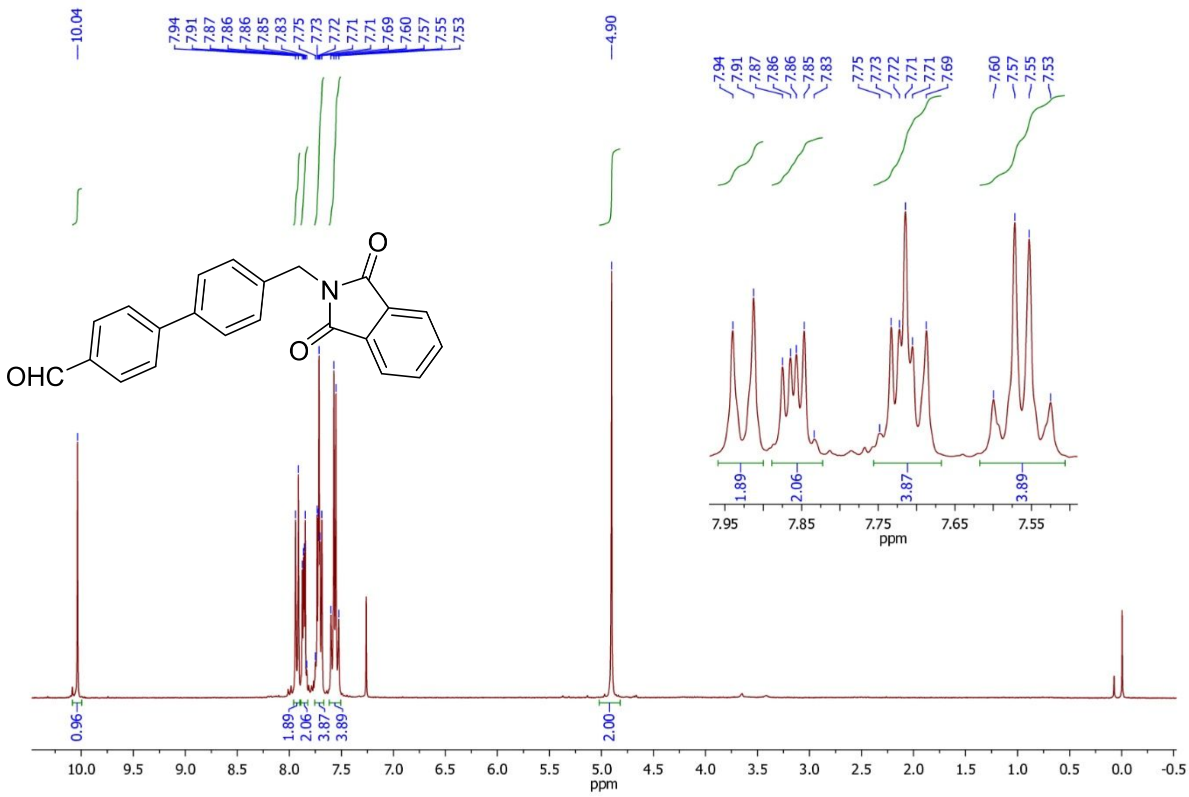

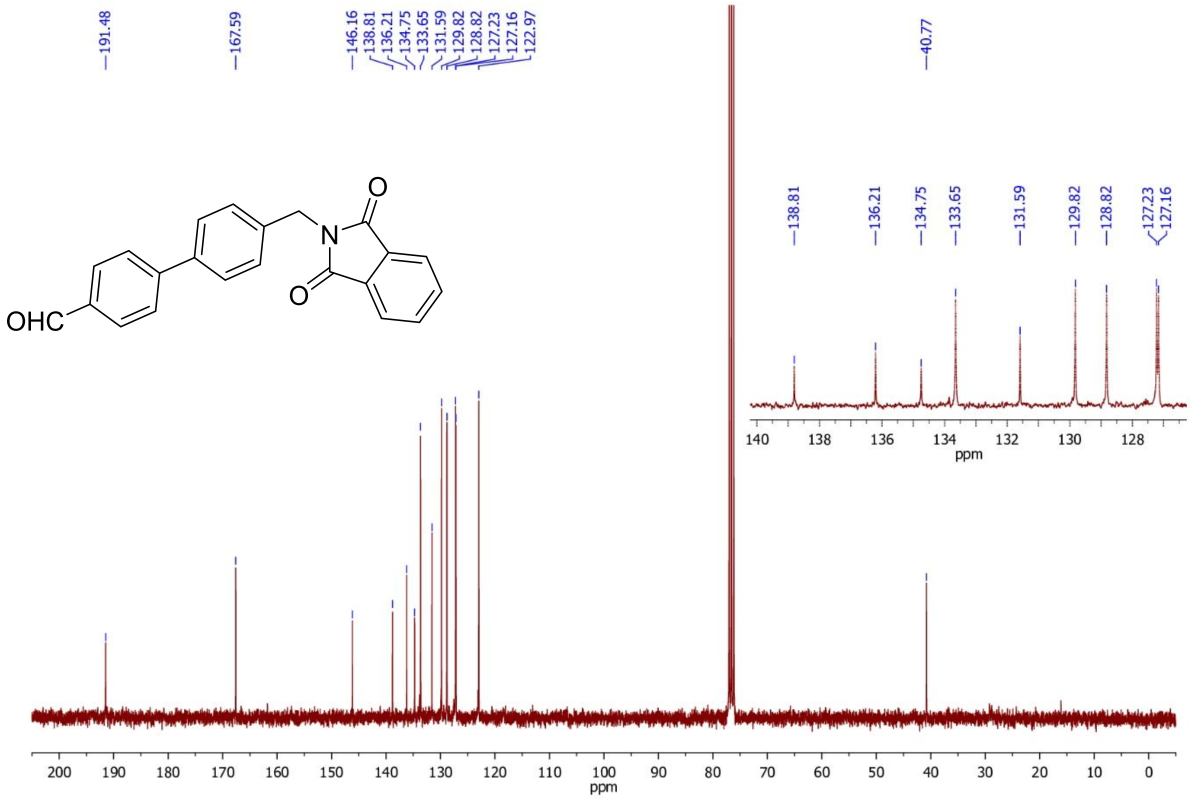

Figure S2. ^{1}H and ^{13}C NMR spectra of compound 3b

```
File          :C:\MSDChem\1\DATA\2022\Dr.Abbas H\Nabiha Abdullah\NAX-55  21-
...            12-22.D
Operator      : Saqib Yasin
Instrument    :    Instrument #2
Acquired      : 21 Dec 2022   13:08      using AcqMethod LIQUID.M
Sample Name: NAX-55
Misc Info     : Temp 120-280 C 10c/min Flow 1.5ml/min Inj 5ul
```

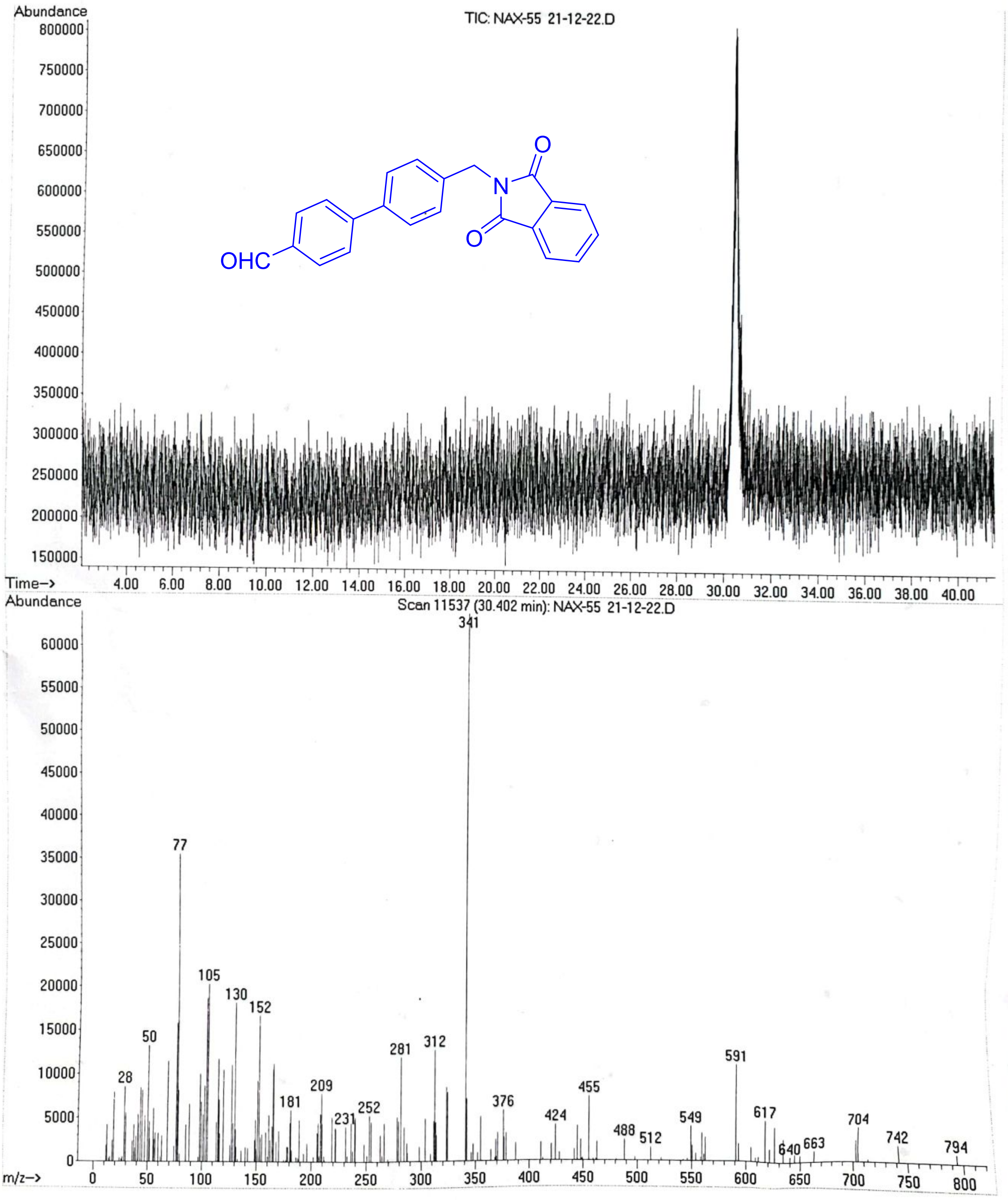

Figure S3. GC-MS of compound 3b

2-((4'-Nitro-[1,1'-biphenyl]-4-yl)methyl)isoindoline-1,3-dione (3c)

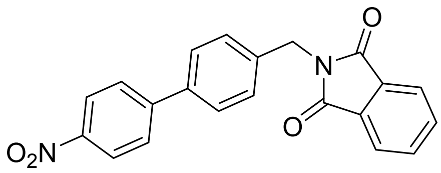

White solid, Yield = 90%, m.p. = 174-176 ^{o}C, R_f = 0.54 (1: 4 :: EtOAc: *n*-Hexane).

^{1}H NMR ($CDCl_3$, 300 MHz) $\delta_{(ppm)}$ = 8.27 (d, *J* = 9.0 Hz, 2H), 7.86 (dd, *J* = 5.4, 3.0 Hz, 2H), 7.87-7.59 (m, 4H), 7.56 (m, 4H), 4.91 (s, 2H).

^{13}C NMR ($CDCl_3$, 75 MHz) $\delta_{(ppm)}$ = 168.0, 147.1, 147.0, 138.3, 137.1, 134.1, 132.0, 129.4, 127.7, 127.7, 127.7, 124.1, 123.4, 41.2.

HRMS-ESI (m/z): $[M+H]^+$ calc'd for $C_{21}H_{15}N_2O_4^+$, 359.1026; found, 359.1028.

FTIR (neat): $\bar{\upsilon}$ (cm^{-1}) = 3041, 2927,1709, 1515, 1387, 1334, 1079, 939, 711.

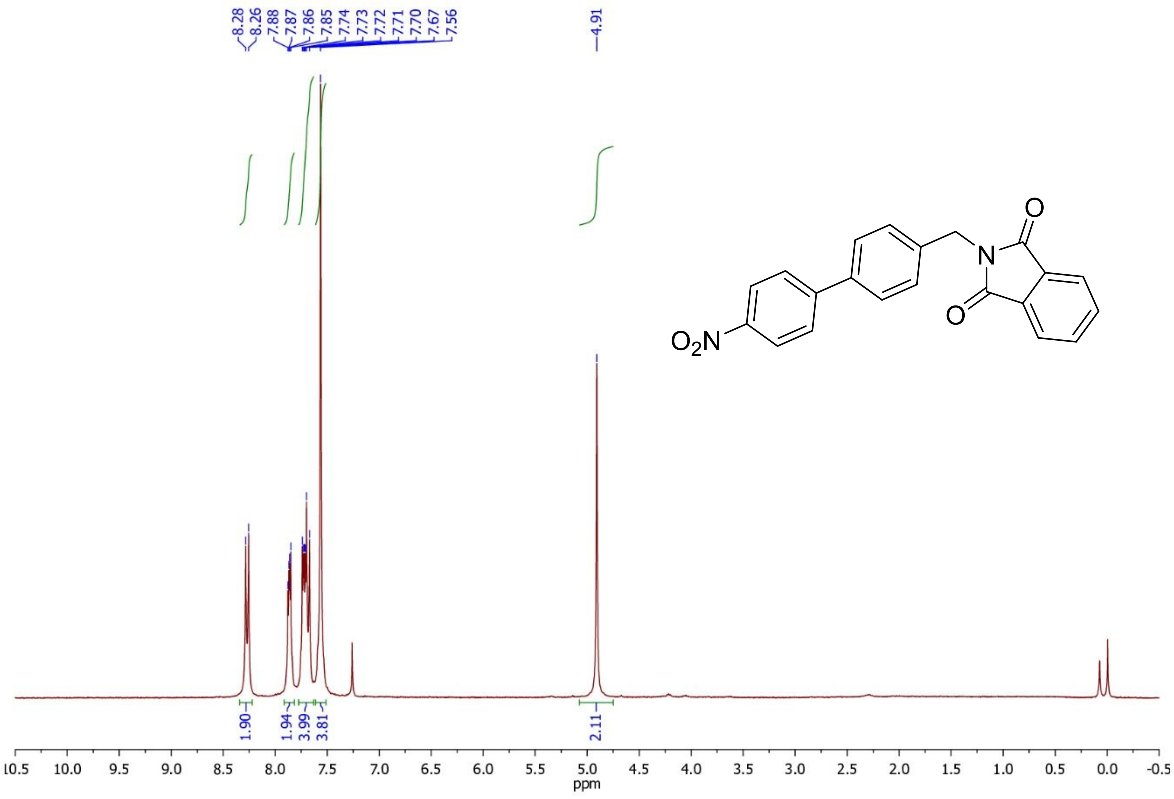

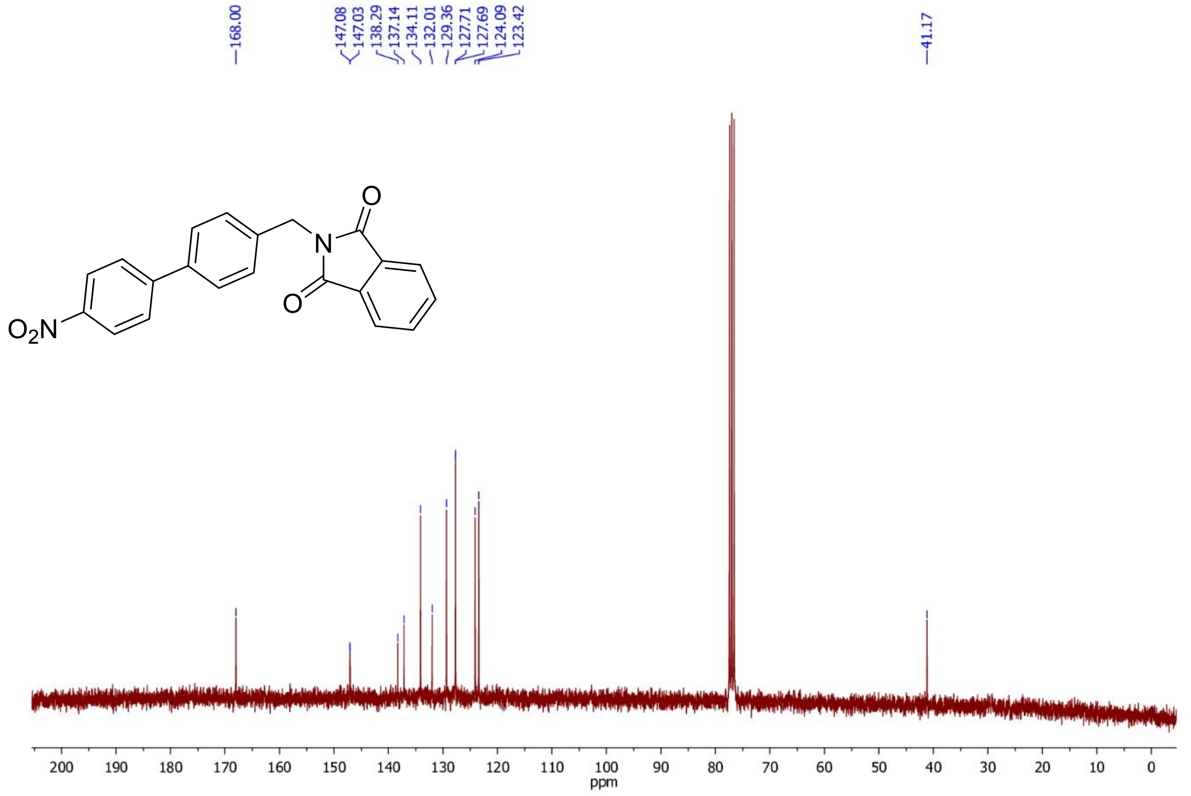

Figure S4. ^{1}H and ^{13}C NMR spectra of compound 3c

4'-((1,3-Dioxoisoindolin-2-yl)methyl)-[1,1'-biphenyl]-4-carbonitrile (3d)

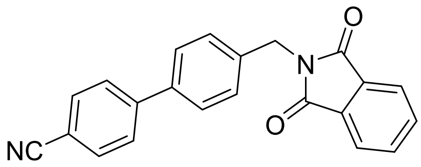

White solid, Yield = 92%, m.p. = 166-168 °C, R_f = 0.26 (1:9 :: EtOAc: *n*-Hexane).

^{1}H NMR ($CDCl_3$, 300 MHz) $\delta_{(ppm)}$ = 7.85-7.81 (m, 2H), 7.71-7.66 (m, 4H), 7.61 (d, *J* = 8.4 Hz, 2H), 7.51 (m, 4H), 4.87 (s, 2H).

^{13}C NMR ($CDCl_3$, 75 MHz) $\delta_{(ppm)}$ = 168.0, 145.0, 138.7, 136.8, 134.1, 132.6, 132.0, 129.3, 127.6, 127.5, 123.4, 118.8, 110.9, 41.1.

GC-EIMS (m/z): 338 (100%), 320, 205, 191, 177, 151, 130, 102, 77.

HRMS-ESI (m/z): $[M+H]^+$ calc'd for $C_{22}H_{15}N_2O_2^+$, 339.1128; found, 339.1133.

FTIR (neat): $\bar{\upsilon}$ (cm^{-1}) = 3041, 2920, 2847, 2218, 1709, 1601, 1394, 1086, 939, 711, 557.

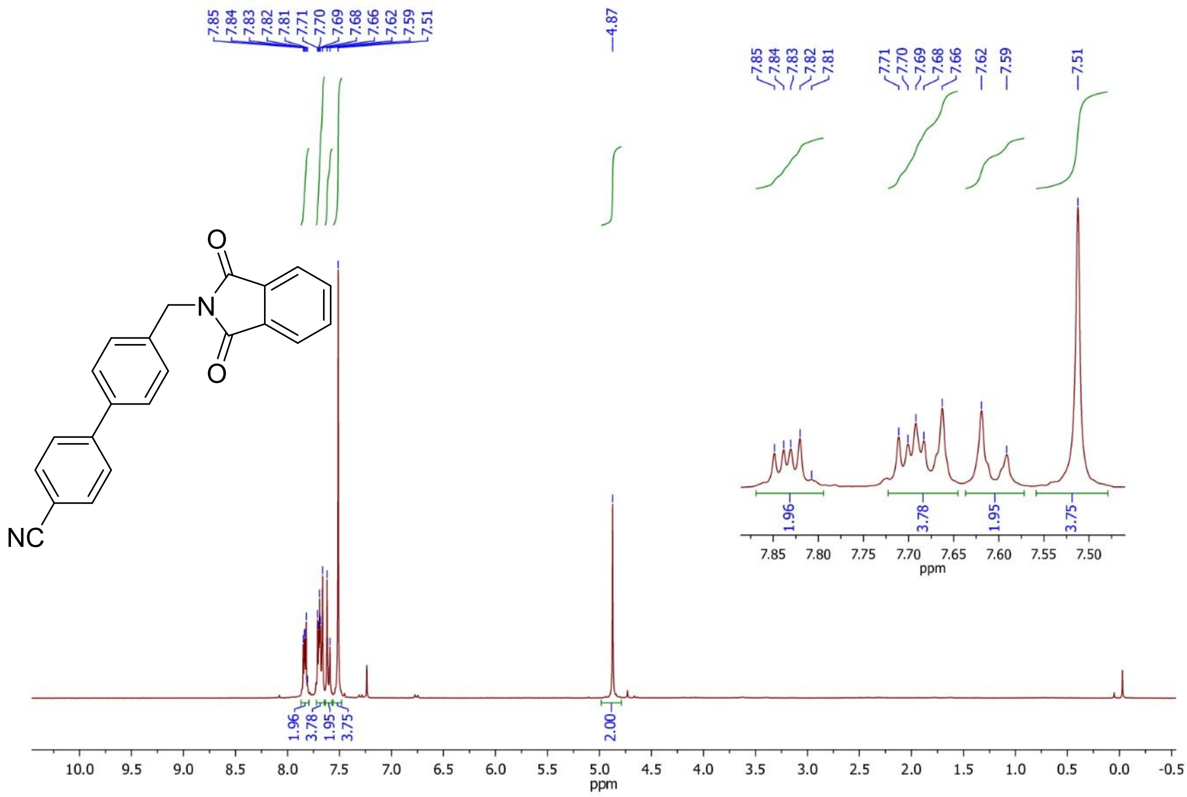

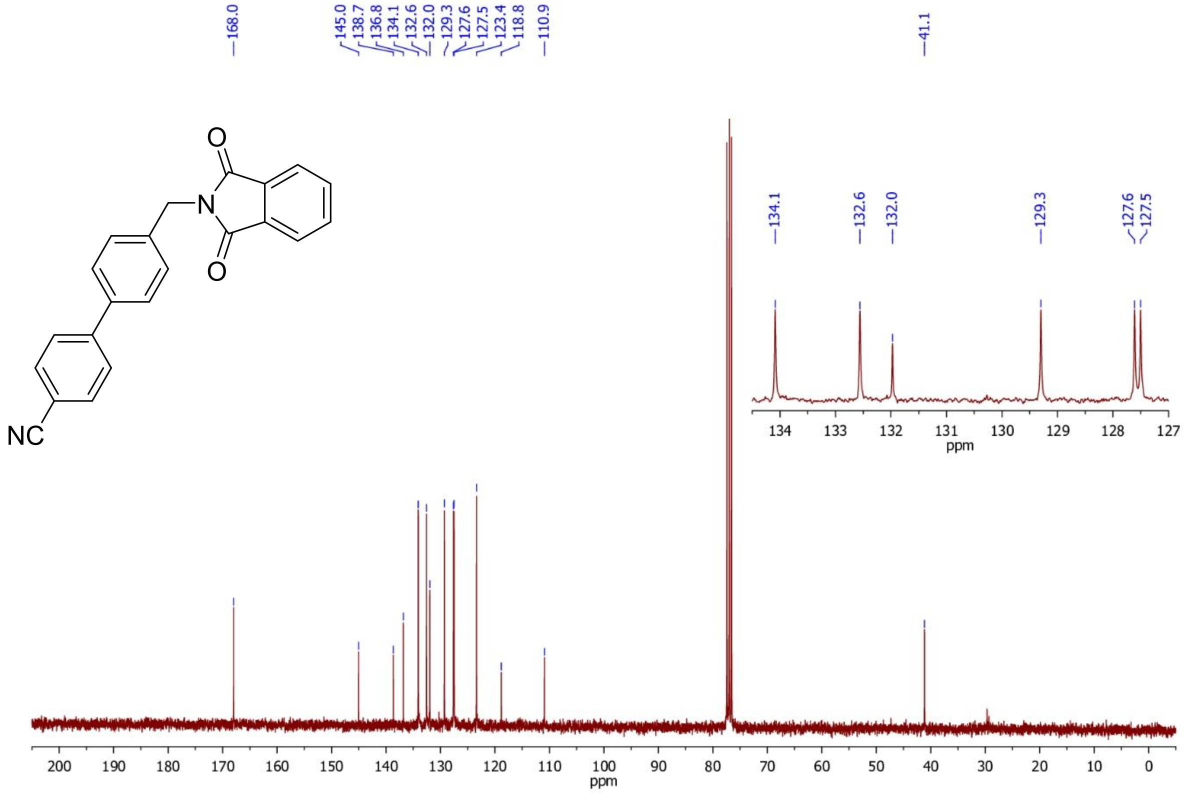

Figure S5. ^{1}H and ^{13}C NMR spectra of compound 3d

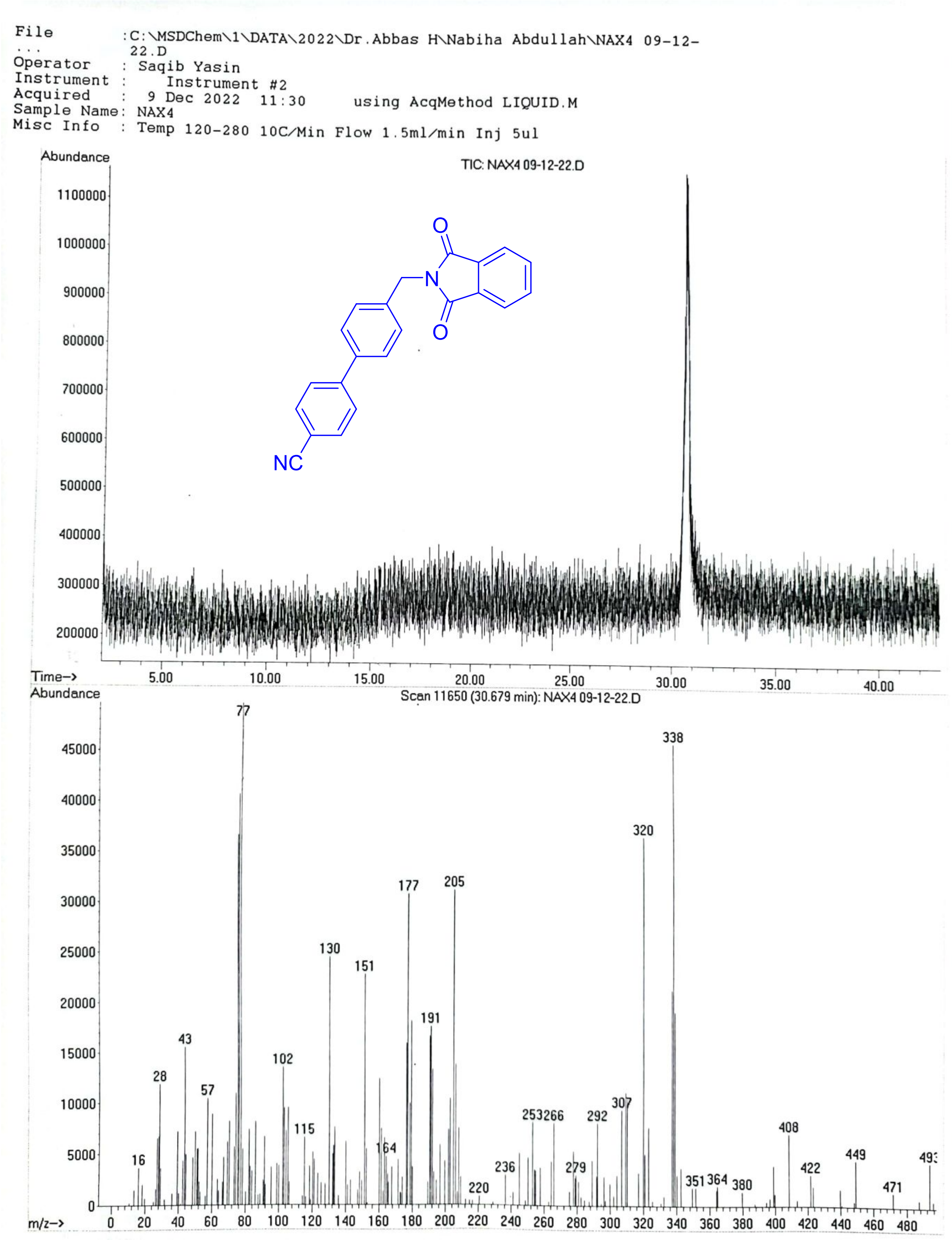

Figure S6. GC-MS of compound 3d

2-((4'-(Trifluoromethyl)-[1,1'-biphenyl]-4-yl)methyl)isoindoline-1,3-dione (3e)

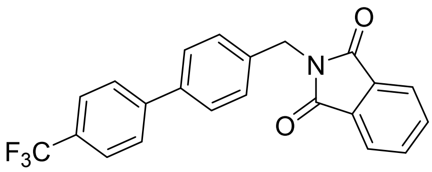

White solid, Yield = 80%, m.p. = 153-154 °C, R_f = 0.32 (1: 9 :: EtOAc: *n*-Hexane).

^{1}H NMR ($CDCl_3$, 300 MHz) $\delta_{(ppm)}$ = 7.89-7.83 (m, 2H), 7.75-7.70 (m, 2H), 7.69-7.62 (m, 4H), 7.54 (m, 4H), 4.90 (s, 2H).

^{13}C NMR ($CDCl_3$, 75 MHz) $\delta_{(ppm)}$ = 168.2, 144.1 (q, $^4J_{CF}$ = 1.5), 139.3, 136.3, 134.1, 132.0, 129.3 (q, $^2J_{CF}$ = 32.25 Hz), 129.2, 127.6, 127.3, 125.7 (q, $^3J_{CF}$ = 3.7 Hz), 124.2 (q, $^1J_{CF}$ = 270.0 Hz), 123.4, 41.2.

GC-EIMS (m/z): 381 (100%), 353, 248, 222, 165, 130, 104, 76, 50.

HRMS-ESI (m/z): $[M+H]^+$ calc'd for $C_{22}H_{15}F_3NO_2^+$, 382.1049; found, 382.1055.

FTIR (neat): $\bar{\upsilon}$ (cm^{-1}) = 3041, 2981, 2920, 1702, 1608, 1401, 1320, 1119, 1066, 932, 805, 718.

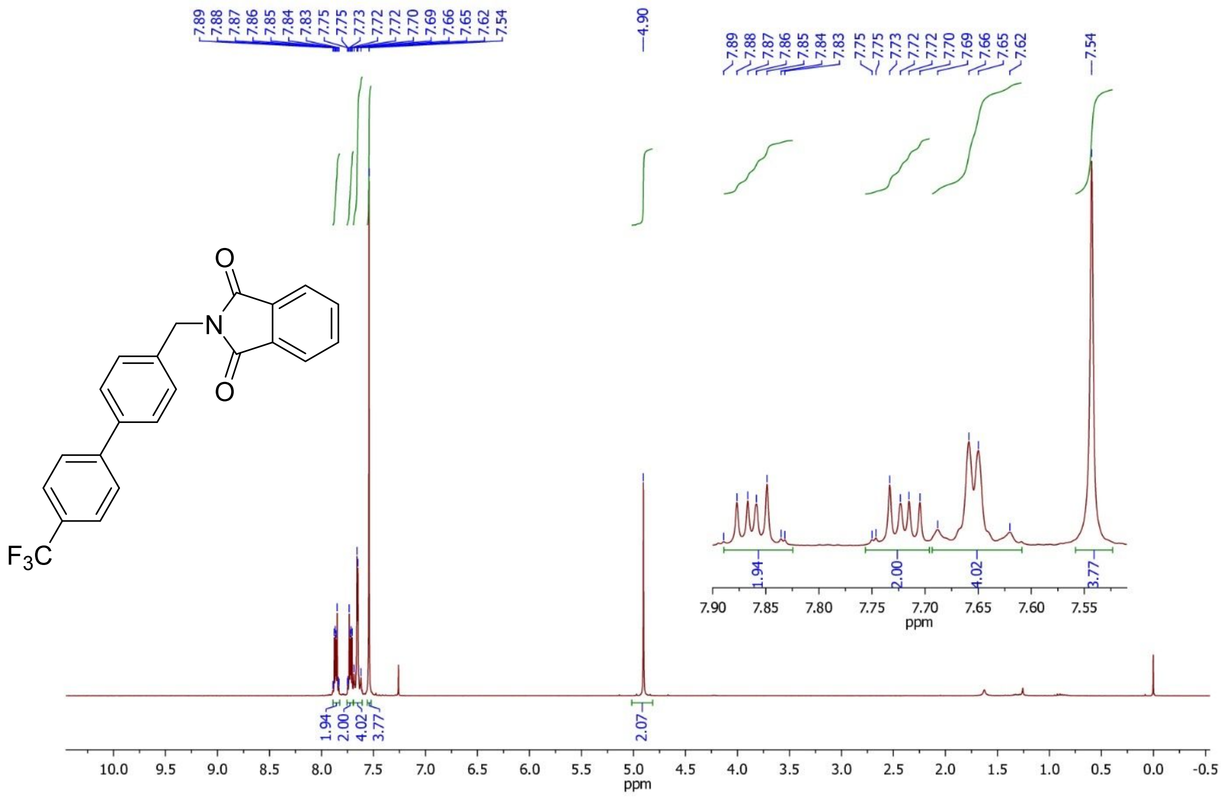

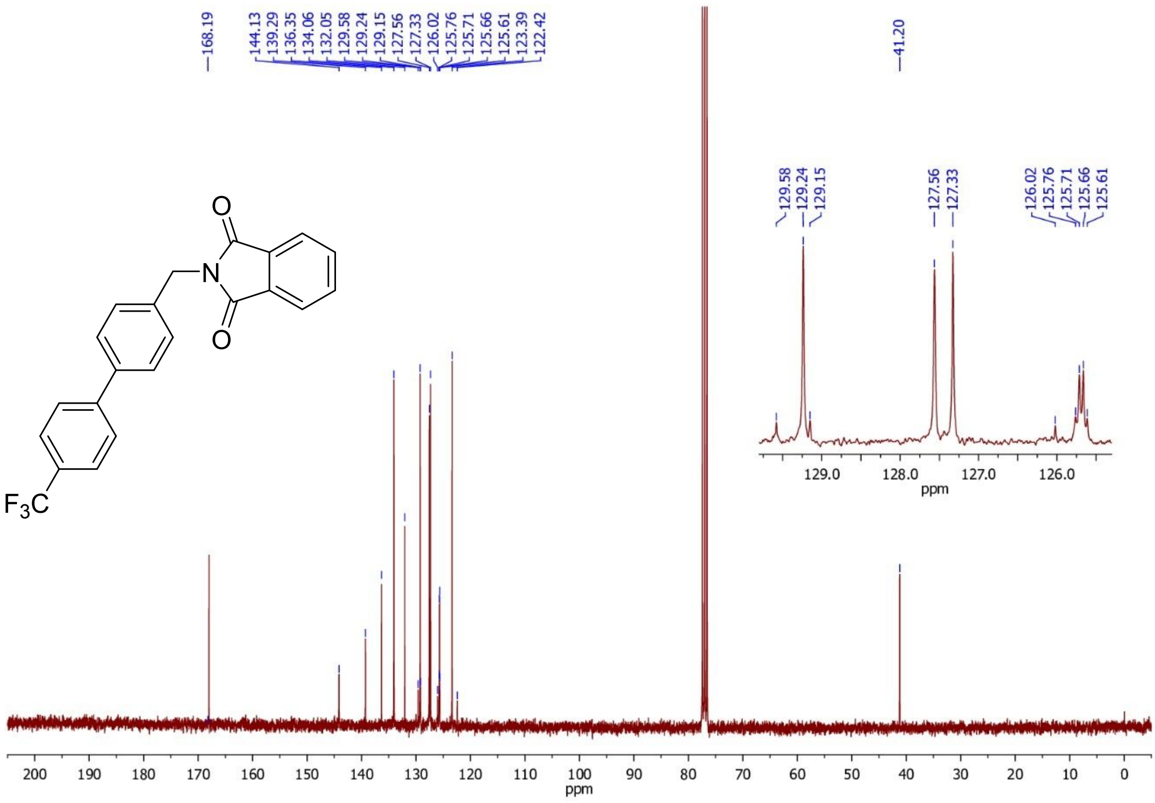

Figure S7. ^{1}H and ^{13}C NMR spectra of compound 3e

```
File        :C:\MSDChem\1\DATA\2022\Dr.Abbas H\Nabiha Abdullah\NAX10 09-12
...          -22.D
Operator    : Saqib Yasin
Instrument  :    Instrument #2
Acquired    :  9 Dec 2022  14:21     using AcqMethod LIQUID.M
Sample Name: NAX10
Misc Info   : Temp 120-280 10C/Min Flow 1.5ml/min Inj 5ul
```

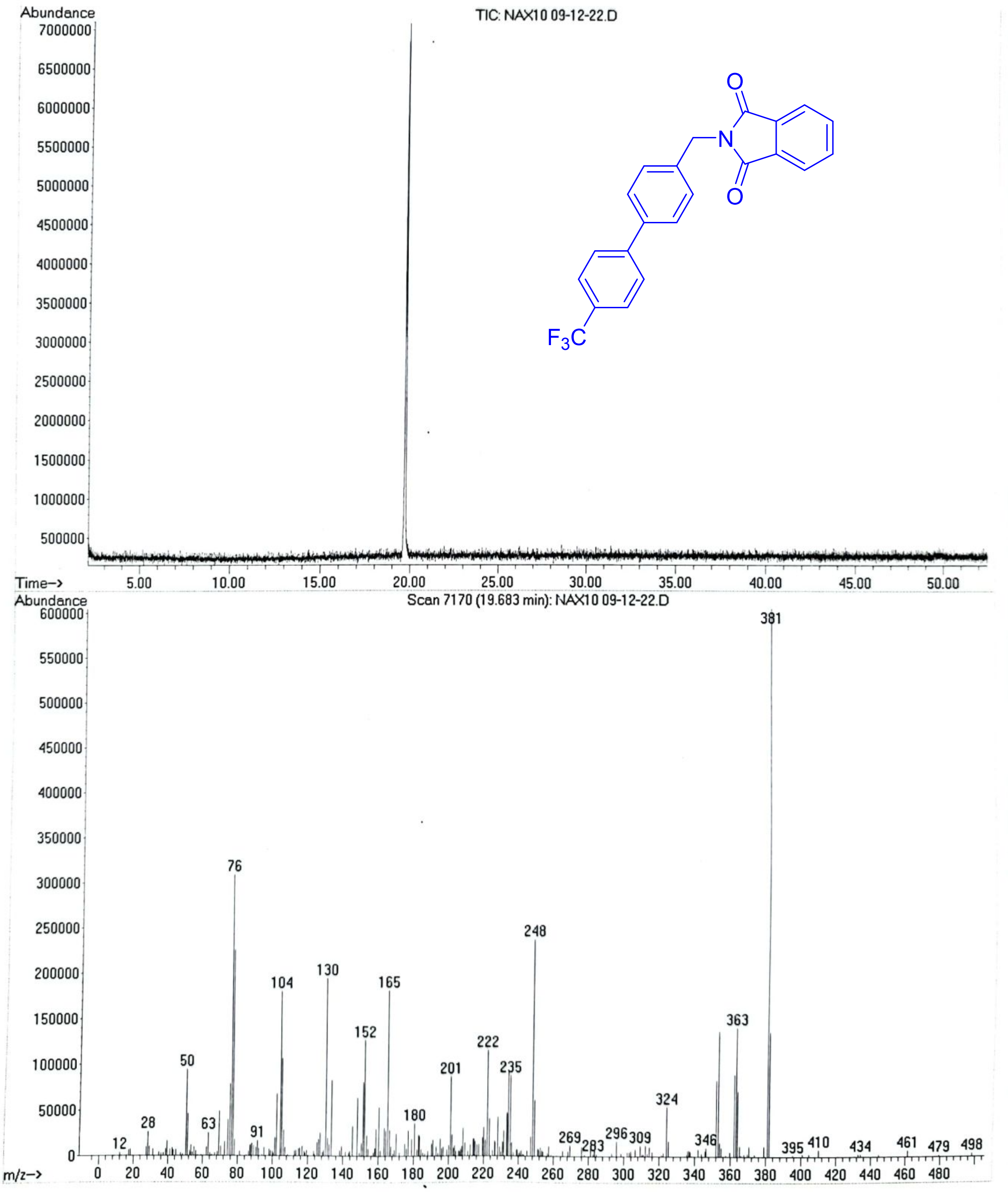

Figure S8. GC-MS of compound 3e

2-((3'-(trifluoromethyl)-[1,1'-biphenyl]-4-yl)methyl)isoindoline-1,3-dione (3f)

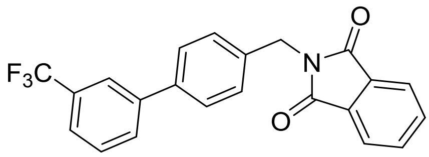

White solid, Yield = 75%, m.p. = 123-124 °C, R_f = 0.35 (1: 4 :: EtOAc: *n*-Hexane).

^{1}H NMR ($CDCl_3$, 300 MHz) $\delta_{(ppm)}$ = 7.88-7.83 (m, 2H), 7.78 (s, 1H), 7.75-7.70 (m, 3H), 7.60-7.50 (m, 6H), 4.90 (s, 2H).

^{13}C NMR ($CDCl_3$, 75 MHz) $\delta_{(ppm)}$ = 168.0, 141.4, 139.3, 136.2, 134.1, 132.0, 131.3 (q, $^{2}J_{CF}$ = 31.5 Hz), 130.3, 129.3, 129.2, 127.5, 124.1 (q, $^{1}J_{CF}$ = 270.75 Hz), 123.9 (q, $^{4}J_{CF}$ = 3.75 Hz), 123.4, 41.2.

GC-EIMS (m/z): 381 (100%), 363, 248, 222, 201, 152, 130, 104, 76, 50.

HRMS-ESI (m/z): $[M+H]^+$ calc'd for $C_{22}H_{15}F_3NO_2$+, 382.1049; found, 382.1052.

FTIR (neat): $\bar{\upsilon}$ (cm^{-1}) = 3055, 2920, 2853, 1709, 1608, 1494, 1320, 1106, 939, 798.

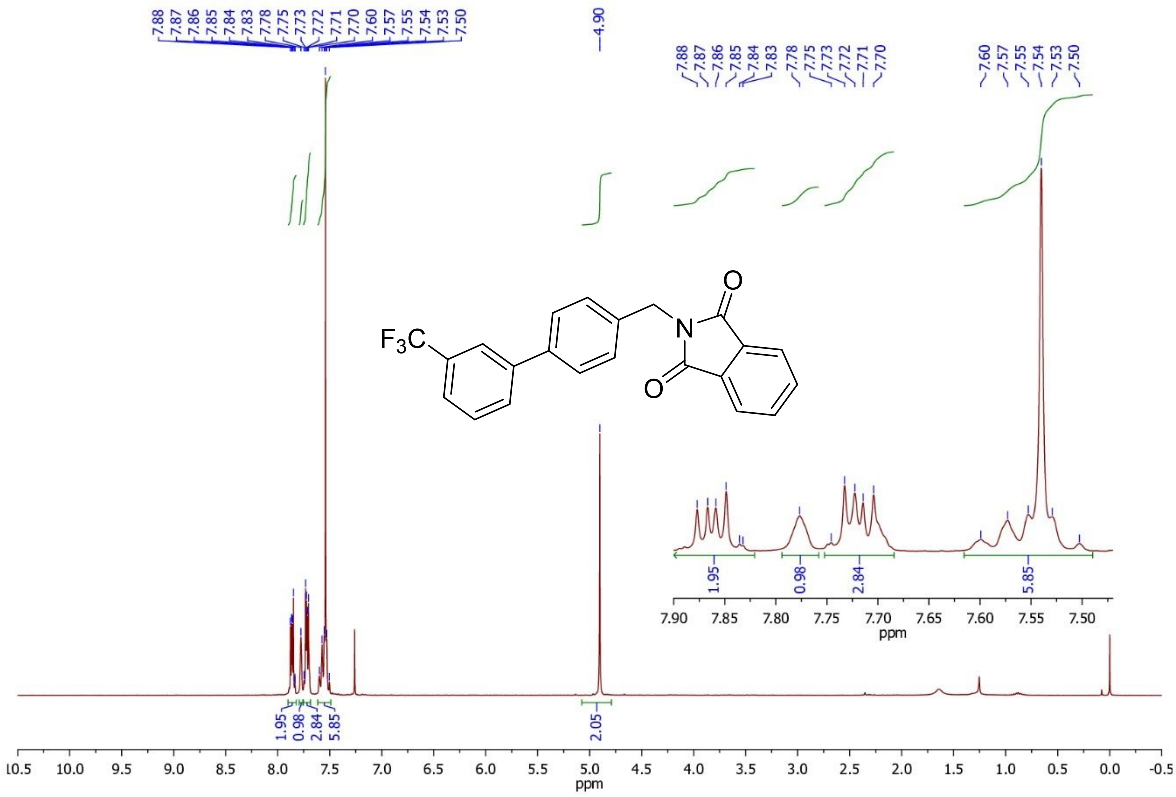

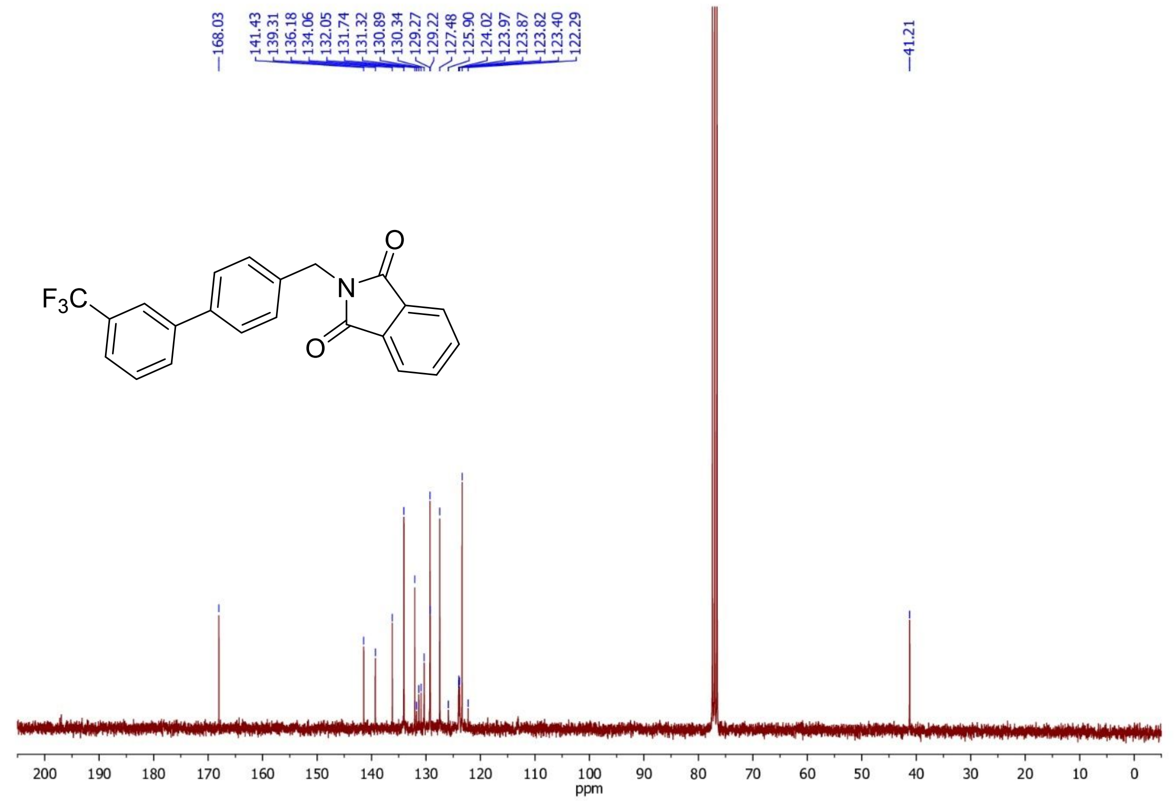

Figure S9. ^{1}H and ^{13}C NMR spectra of compound 3f

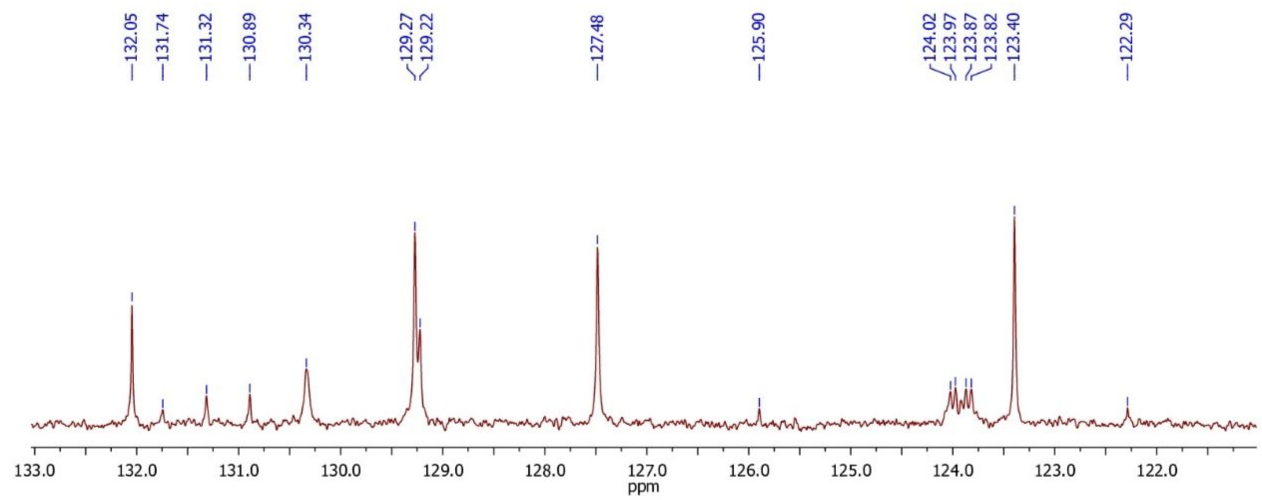

Figure S10. Expansion of ^{13}C NMR of compound 3f

File :C:\MSDChem\1\DATA\2022\Dr.Abbas H\Nabiha Abdullah\NAX 49 21-1
... 2-22.D
Operator : Saqib Yasin
Instrument : Instrument #2
Acquired : 21 Dec 2022 10:23 using AcqMethod LIQUID.M
Sample Name: NAX-49
Misc Info : Temp 120-280 C 10c/min Flow 1.5ml/min Inj 5ul

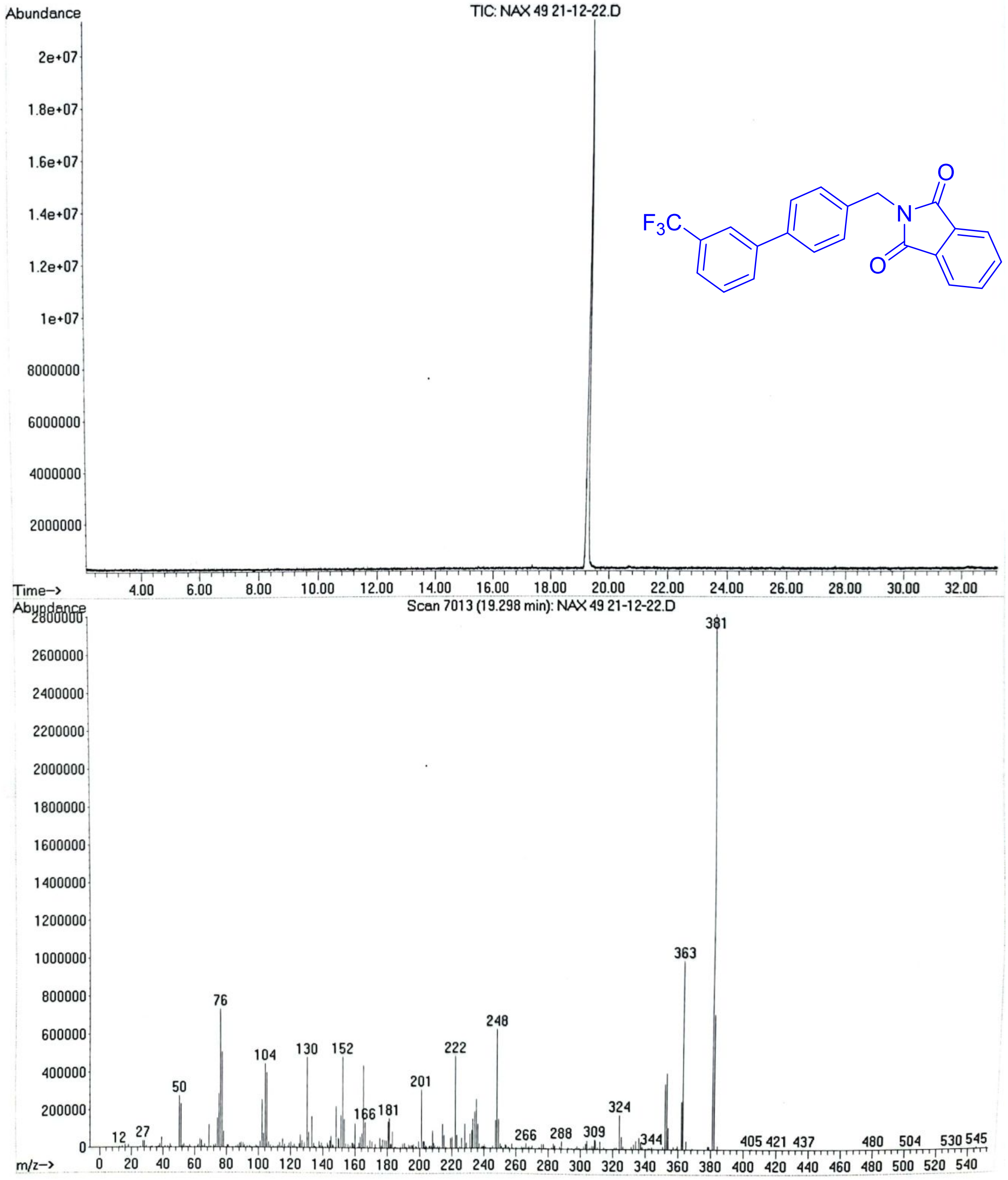

Figure S11. GC-MS of compound 3f

2-(4'-((1,3-Dioxoisoindolin-2-yl)methyl)-[1,1'-biphenyl]-4-yl)acetonitrile (3g)

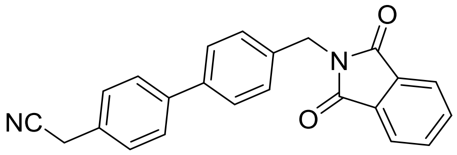

White solid, Yield = 95%, m.p. = 171-173 °C, R_f = 0.38 (3: 7 :: EtOAc: *n*-Hexane).

^{1}H NMR ($CDCl_3$, 300 MHz) $\delta_{(ppm)}$ = 7.87-7.82 (m, 2H), 7.74-7.70 (m, 2H), 7.57-7.48 (m, 6H), 7.38 (d, *J* = 8.4 Hz, 2H), 4.89 (s, 2H), 3.78 (s, 2H).

^{13}C NMR ($CDCl_3$, 75 MHz) $\delta_{(ppm)}$ = 168.0, 140.5, 139.7, 135.7, 134.0, 132.0, 129.1, 128.9, 128.3, 127.7, 127.3, 123.4, 117.7, 41.2, 23.3.

GC-EIMS (m/z): 352 (100%), 218, 163, 139, 105, 76, 55, 34.

HRMS-ESI (m/z): $[M+H]^+$ calc'd for $C_{23}H_{17}N_2O_2^+$, 353.1285; found, 353.1290.

FTIR (neat): $\bar{\upsilon}$ (cm^{-1}) = 3061, 2920, 2847, 2244, 1709, 1501, 1394, 1086, 939, 711, 523.

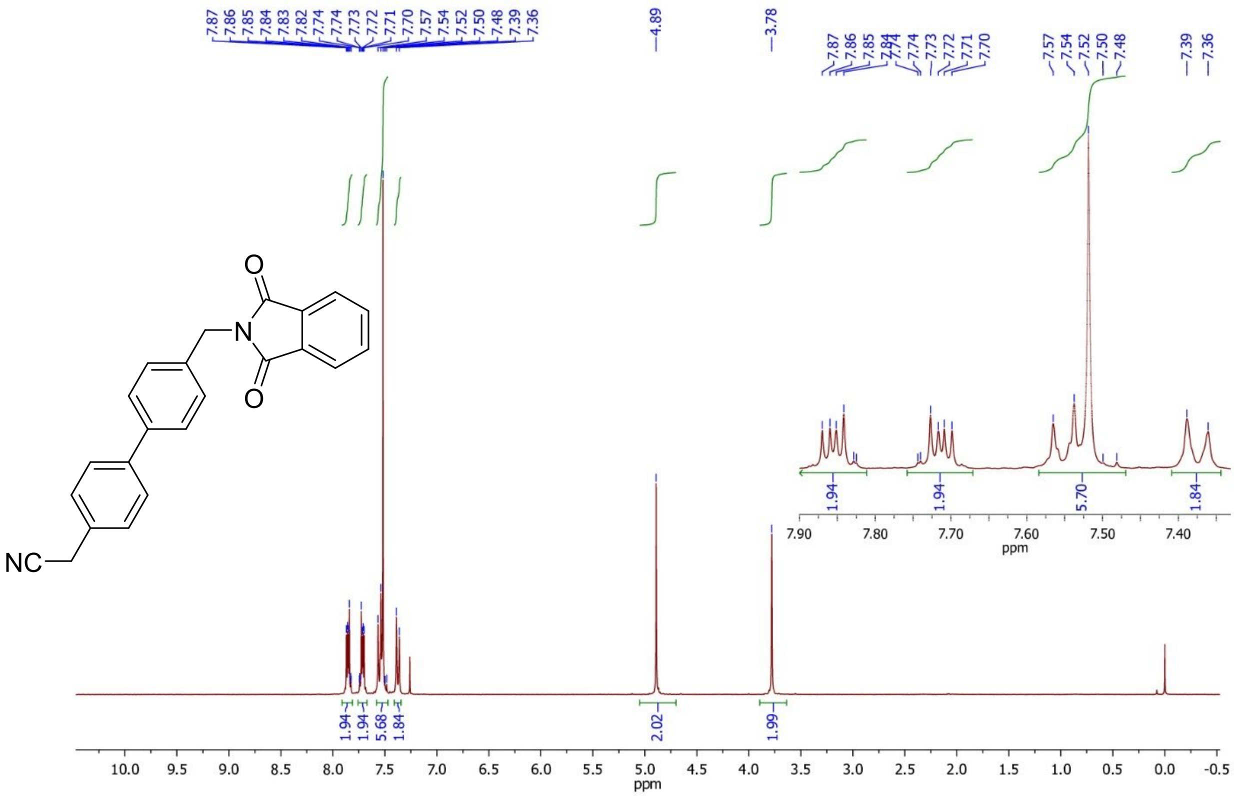

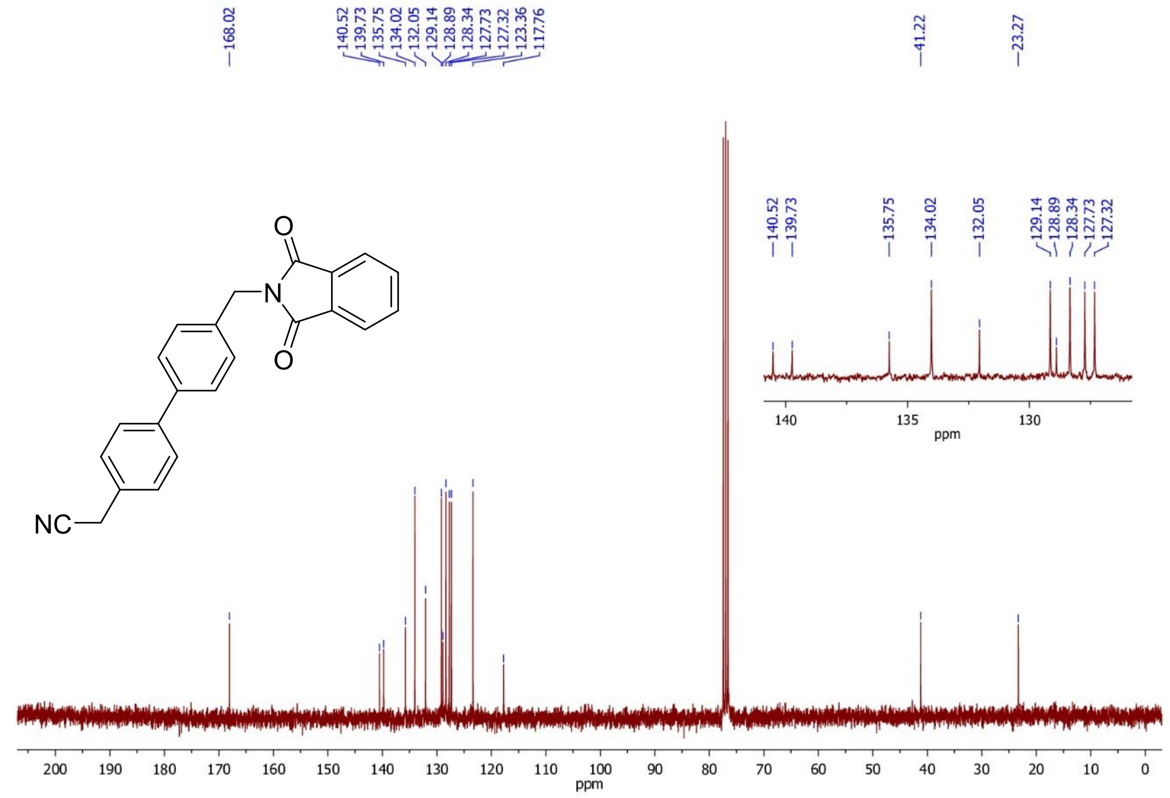

Figure S12. ^{1}H and ^{13}C NMR spectra of compound 3g

File :C:\MSDChem\1\DATA\2022\Dr.Abbas H\Nabiha Abdullah\NAX41 10-12
... -22.D
Operator : Saqib Yasin
Instrument : Instrument #2
Acquired : 10 Dec 2022 11:33 using AcqMethod LIQUID.M
Sample Name: NAX41
Misc Info : Temp 120-280 10C/Min Flow 1.5ml/min Inj 5ul

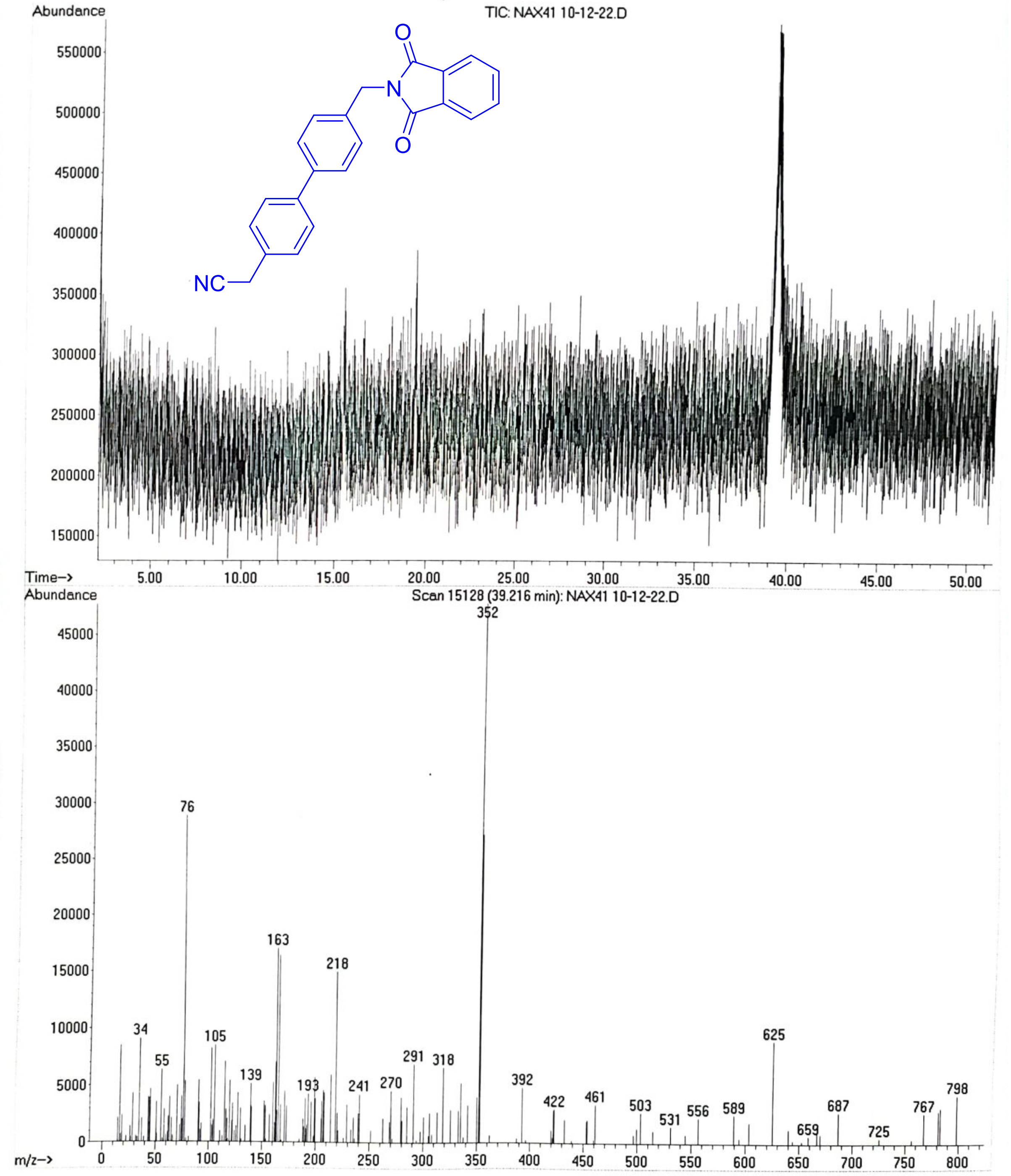

Figure S13. GC-MS of compound 3g

2-((3',5'-Dimethyl-[1,1'-biphenyl]-4-yl)methyl)isoindoline-1,3-dione (3h)

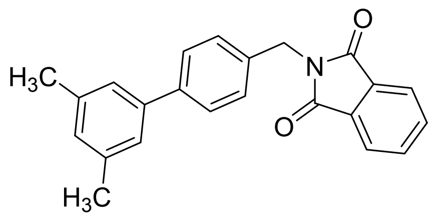

White solid, Yield = 78%, m.p. = 124-126 °C, R_f = 0.49 (1: 4 :: EtOAc: *n*-Hexane).

^{1}H NMR ($CDCl_3$, 300 MHz) $\delta_{(ppm)}$ = 7.86 (app dd, *J* = 5.4, 3.0 Hz, 2H), 7.72 (app dd, *J* = 5.4, 3.0 Hz, 2H), 7.54-7.48 (m, 4H), 7.16 (s, 2H), 6.98 (s, 1H), 4.89 (s, 2H), 2.36 (s, 6H).

^{13}C NMR ($CDCl_3$, 75 MHz) $\delta_{(ppm)}$ = 168.1, 141.0, 140.6, 138.2, 135.1, 134.0, 132.1, 129.0, 127.4, 125.0, 123.3, 41.3, 21.4.

GC-EIMS (m/z): 341 (100%), 326, 208, 179, 165, 130, 104, 76.

HRMS-ESI (m/z): $[M+H]^+$ calc'd for $C_{23}H_{20}NO_2^+$, 342.1489; found, 342.1494.

FTIR (neat): $\bar{\upsilon}$ (cm^{-1}) = 3068, 2920, 2847, 1702, 1595, 1434, 1387, 1106, 959, 718, 530.

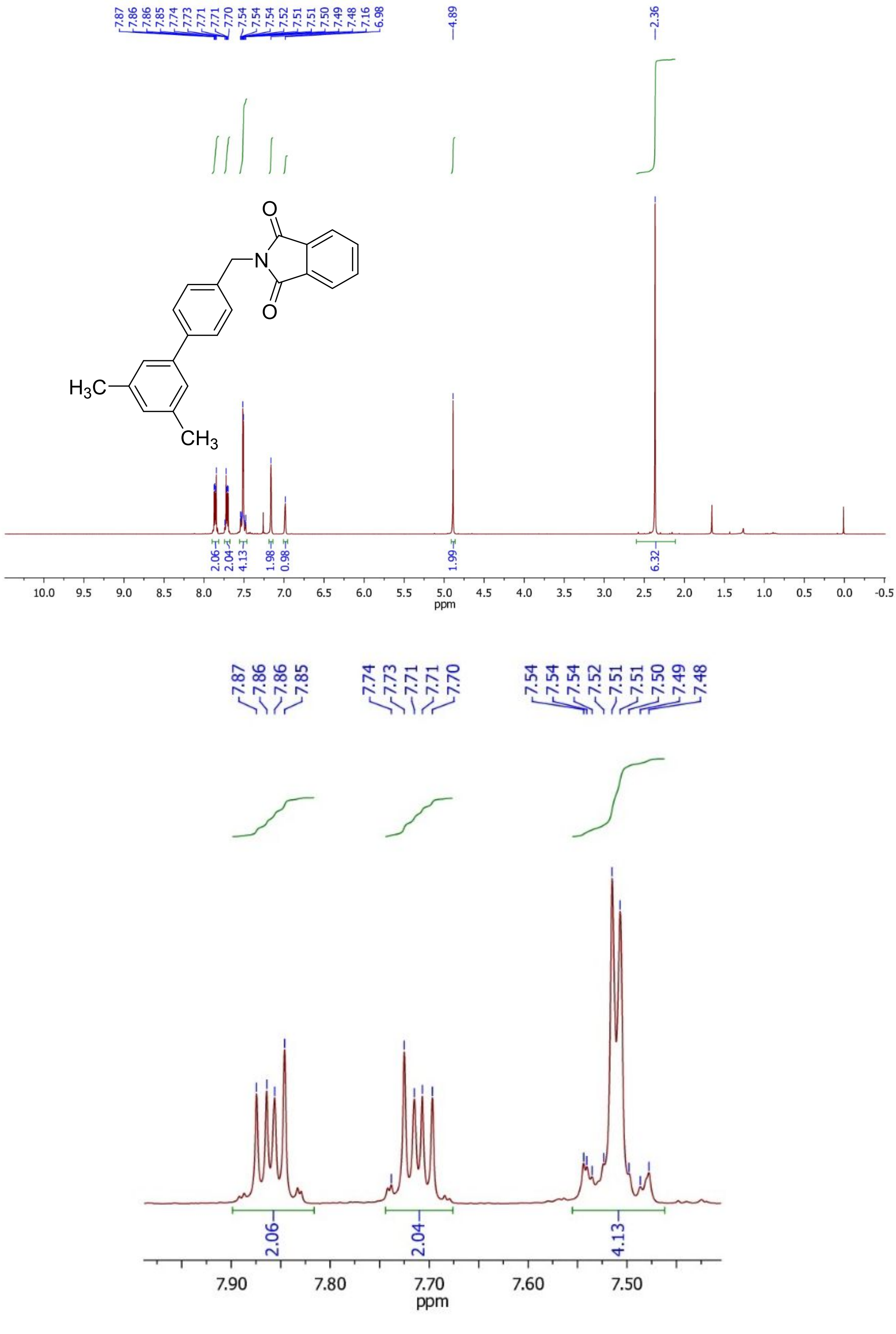

Figure S14. ^{1}H NMR spectra of compound 3h

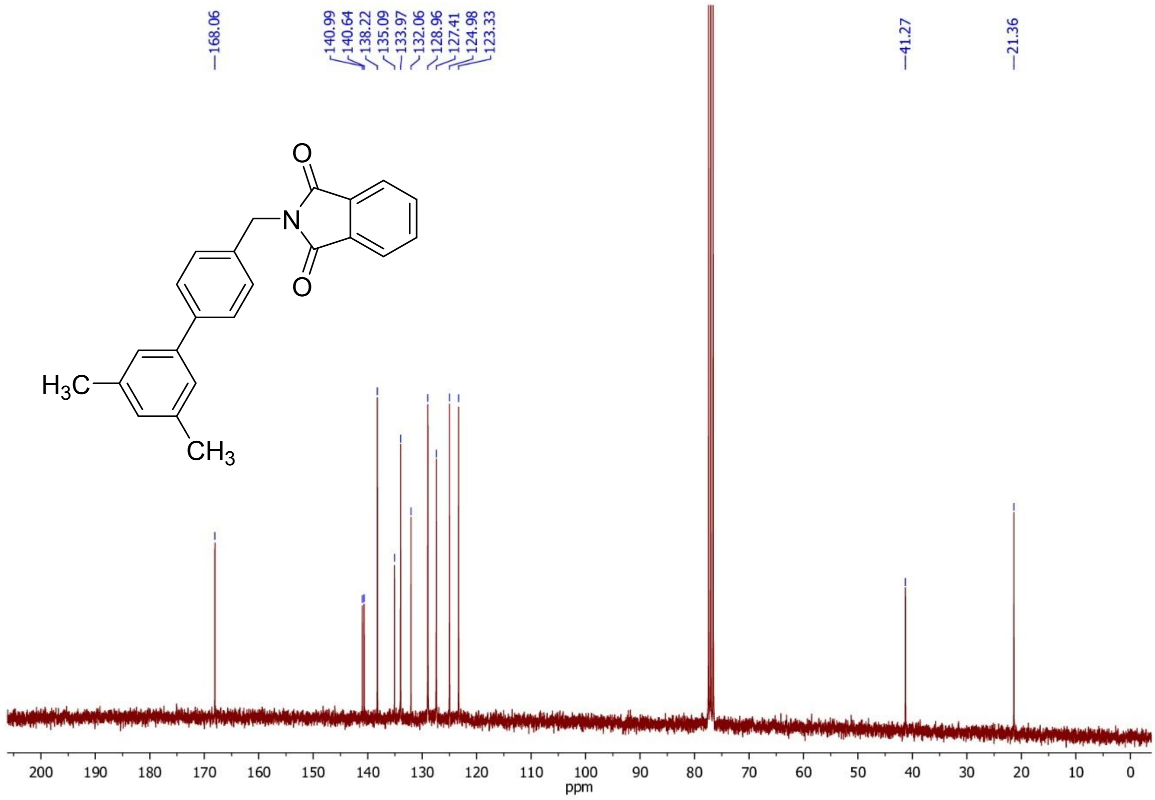

Figure S15. ^{13}C NMR spectrum of compound 3h

```
File         :C:\MSDChem\1\DATA\2023\Dr. Abbas Hassan\Nabiha Abdullah\NAX-4
...           7 01-06-23.D
Operator     : Saqib Yasin
Instrument   :    Instrument #2
Acquired     :  1 Jun 2023   12:47      using AcqMethod LIQUID.M
Sample Name: NAX-47
Misc Info    : Temp 120-280 C 10C/min Flow 1.5ml/min Inj 5ul
```

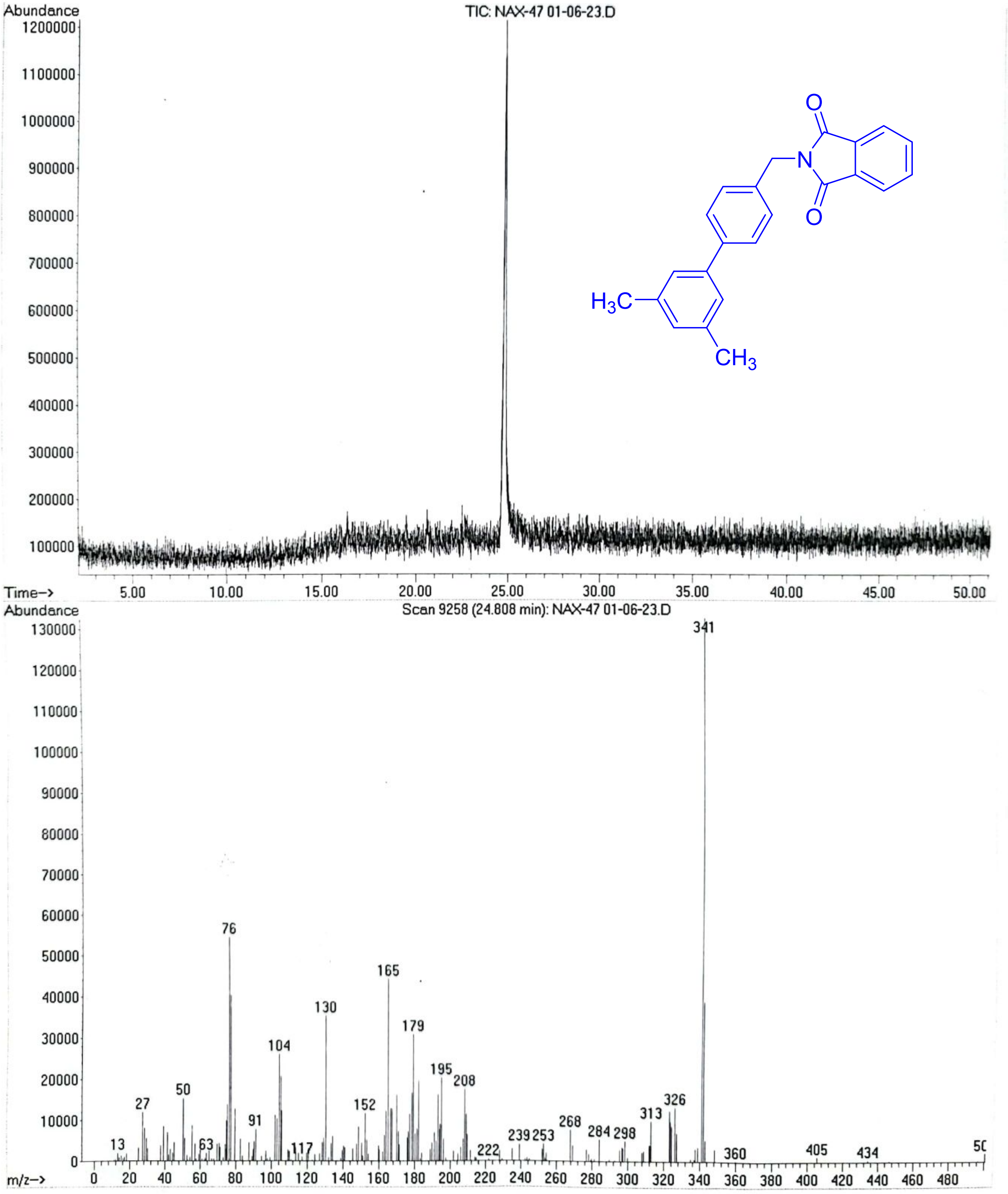

Figure S16. GC-MS of compound 3h

2-((2',4',6'-Trimethyl-[1,1'-biphenyl]-4-yl)methyl)isoindoline-1,3-dione (3i)

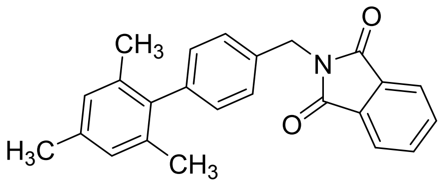

White solid, Yield = 80%, m.p. = 114-116 °C, R_f = 0.48 (1: 9 :: EtOAc: *n*-Hexane).

^{1}H NMR ($CDCl_3$, 300 MHz) $\delta_{(ppm)}$ = 7.91-7.85 (m, 2H), 7.76-7.71 (m, 2H), 7.47 (d, *J* = 8.1 Hz, 2H), 7.10 (m, 2H), 6.92 (s, 2H), 4.90 (s, 2H), 2.32 (s, 3H), 1.97 (s, 6H).

^{13}C NMR ($CDCl_3$, 75 MHz) $\delta_{(ppm)}$ = 168.1, 140.6, 138.5, 136.6, 136.0, 134.5, 134.0, 132.1, 129.6, 128.5, 128.0, 123.4, 41.4, 29.7, 20.8.

GC-EIMS (m/z): 355 (100%), 222, 193, 165, 130, 104, 76, 50.

HRMS-ESI (m/z): $[M+H]^+$ calc'd for $C_{24}H_{22}NO_2^+$, 356.1645; found, 355.1649.

FTIR (neat): $\bar{\upsilon}$ (cm^{-1}) = 3028, 2913, 2847, 1709, 1468, 1327, 1079, 932, 718, 530.

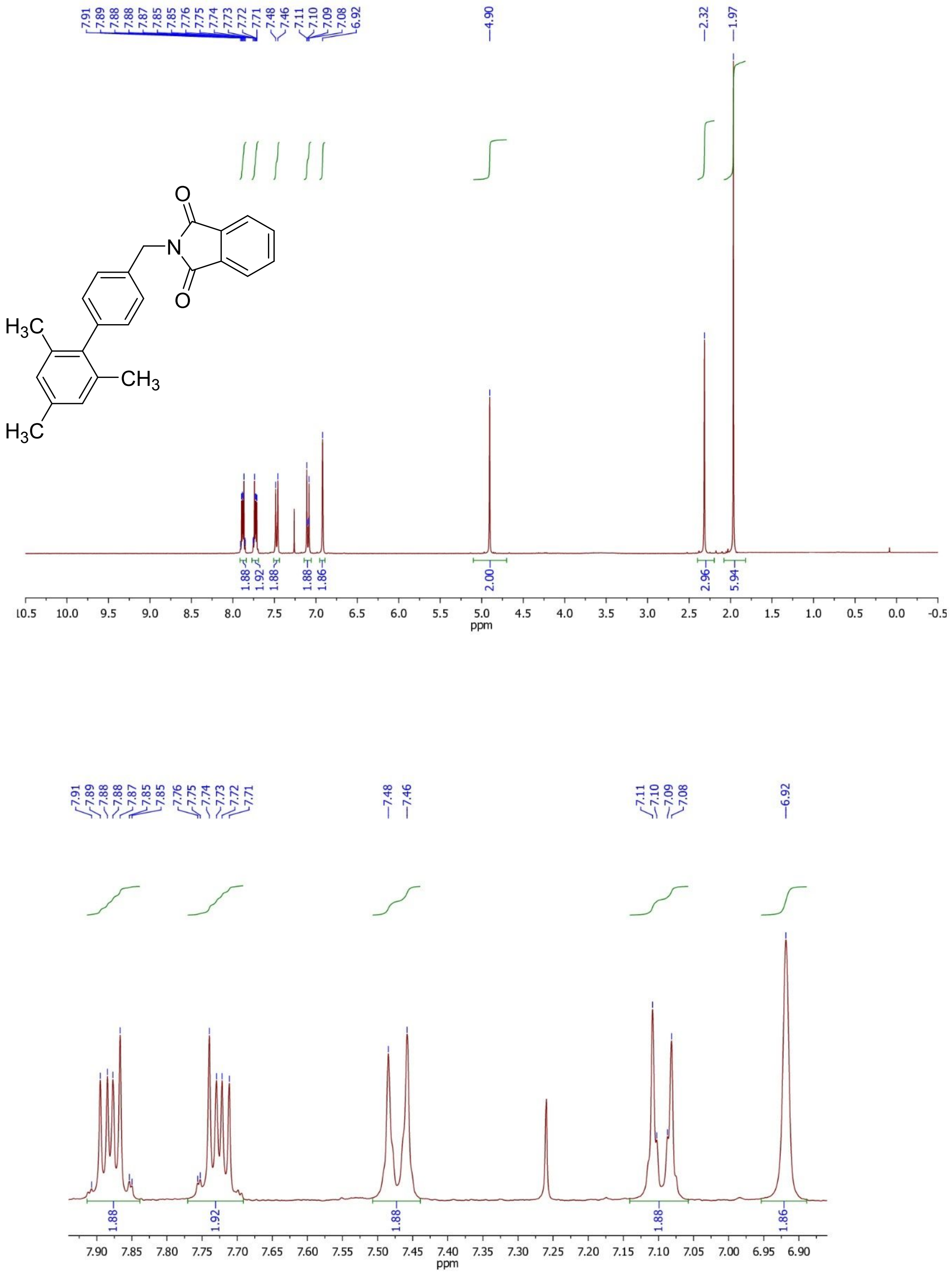

Figure S17. ^{1}H NMR spectra of compound 3i

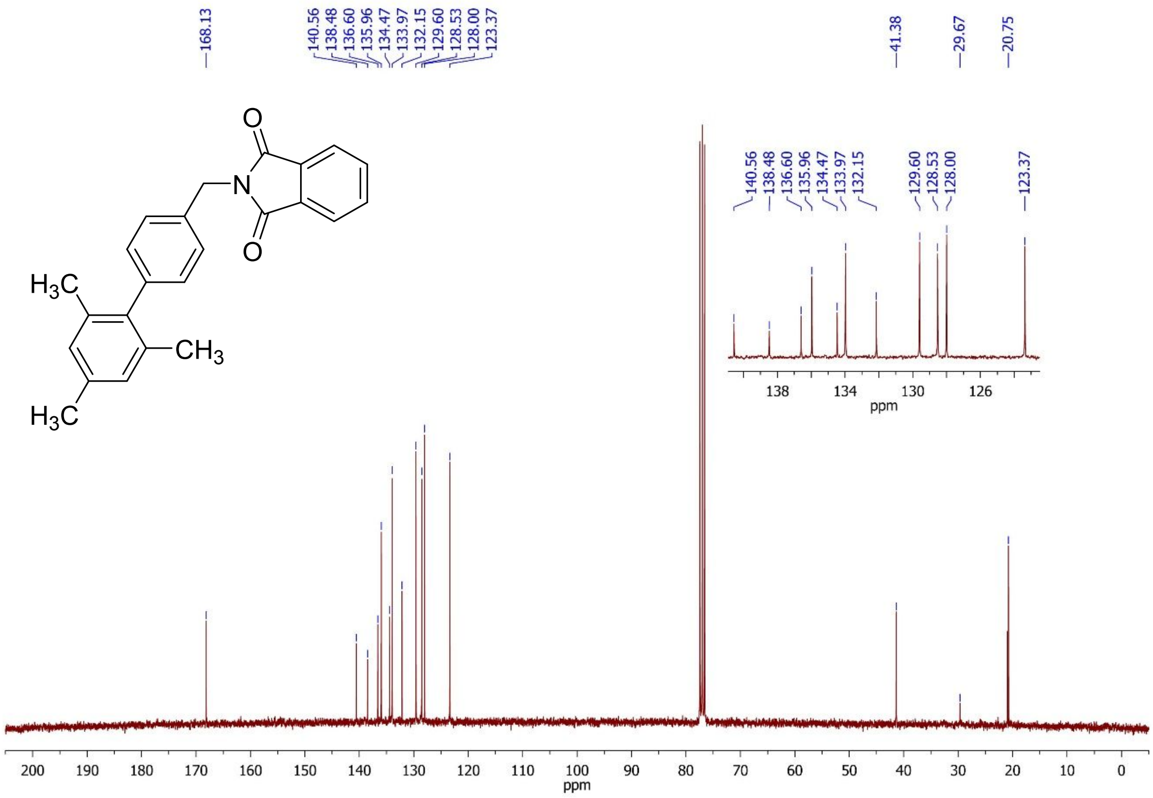

Figure S18.^{13}C NMR spectrum of compound 3i

File :C:\MSDChem\1\DATA\2022\Dr.Abbas H\Nabiha Abdullah\NAX-52 21-
... 12-22.D
Operator : Saqib Yasin
Instrument : Instrument #2
Acquired : 21 Dec 2022 11:00 using AcqMethod LIQUID.M
Sample Name: NAX-52
Misc Info : Temp 120-280 C 10c/min Flow 1.5ml/min Inj 5ul

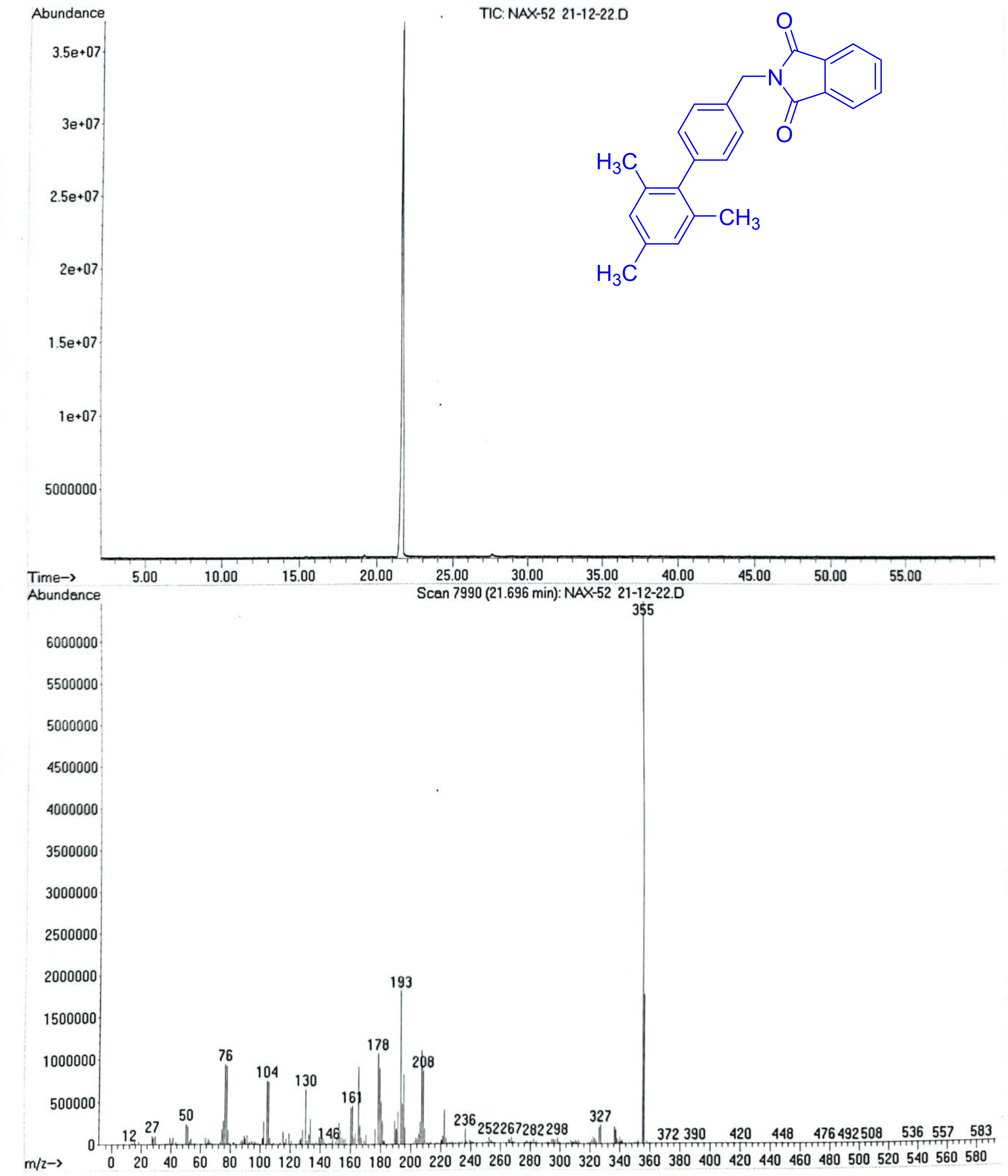

Figure S19. GC-MS of compound 3i

2-(4-(Naphthalen-1-yl)benzyl)isoindoline-1,3-dione (3j)

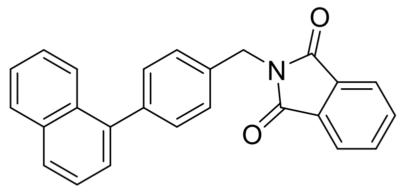

White solid, Yield = 95%, m.p. = 167-169 °C, R_f = 0.37 (1: 9 :: EtOAc: *n*-Hexane).

^{1}H NMR ($CDCl_3$, 300 MHz) $\delta_{(ppm)}$ = 7.92-7.83 (m, 5H), 7.76-7.70 (m, 2H), 7.57 (d, *J* = 8.1, 2H), 7.53-7.43 (m, 4H), 7.41-7.36 (m, 2H), 4.96 (s, 2H).

^{13}C NMR ($CDCl_3$, 75 MHz) $\delta_{(ppm)}$ = 168.1, 140.3, 139.7, 135.3, 134.0, 133.7, 132.1, 131.5, 130.3, 128.5, 128.2, 127.7, 126.9, 126.0, 125.9, 125.7, 125.3, 123.4, 41.4.

GC-EIMS (m/z): 363 (100%), 344, 289, 228, 202, 160, 130, 104, 76, 50.

HRMS-ESI (m/z): $[M+H]^+$ calc'd for $C_{25}H_{18}NO_2^+$, 364.1332; found, 364.1338.

FTIR (neat): $\bar{\upsilon}$ (cm^{-1}) = 3055, 2927, 1716, 1428, 1394, 1086, 932, 778, 718.

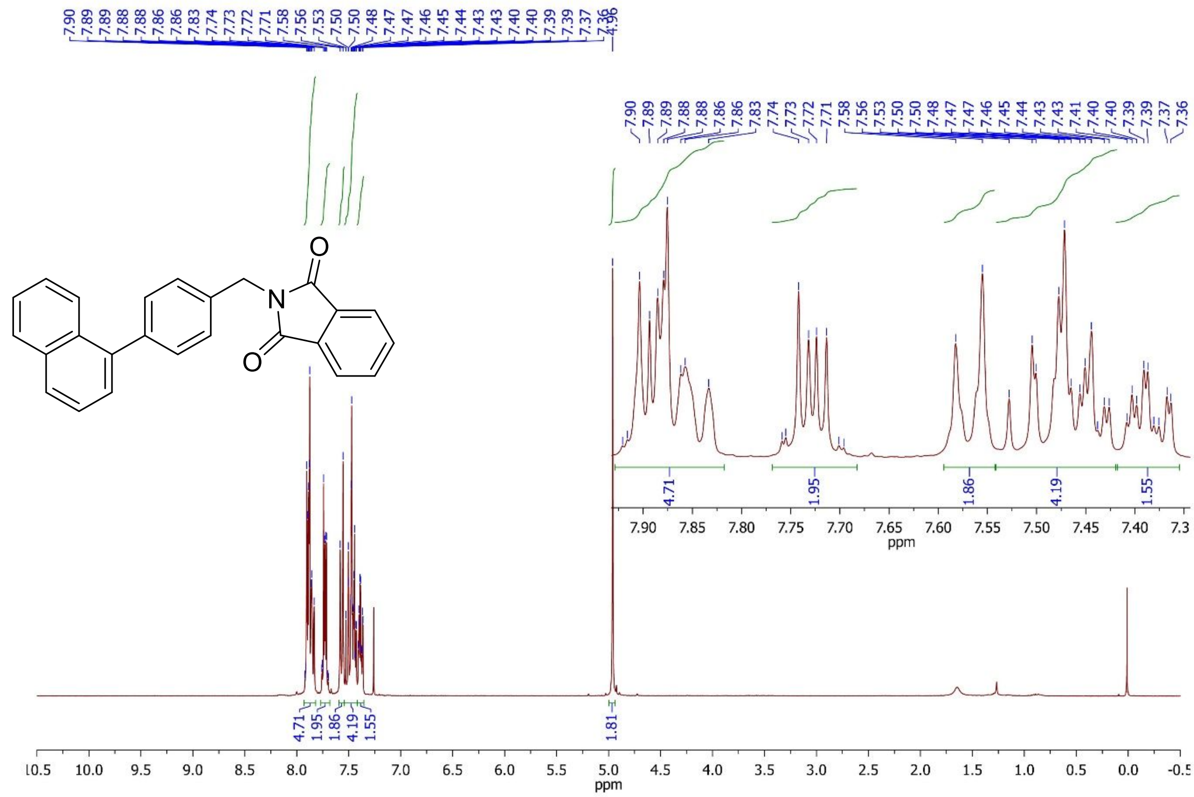

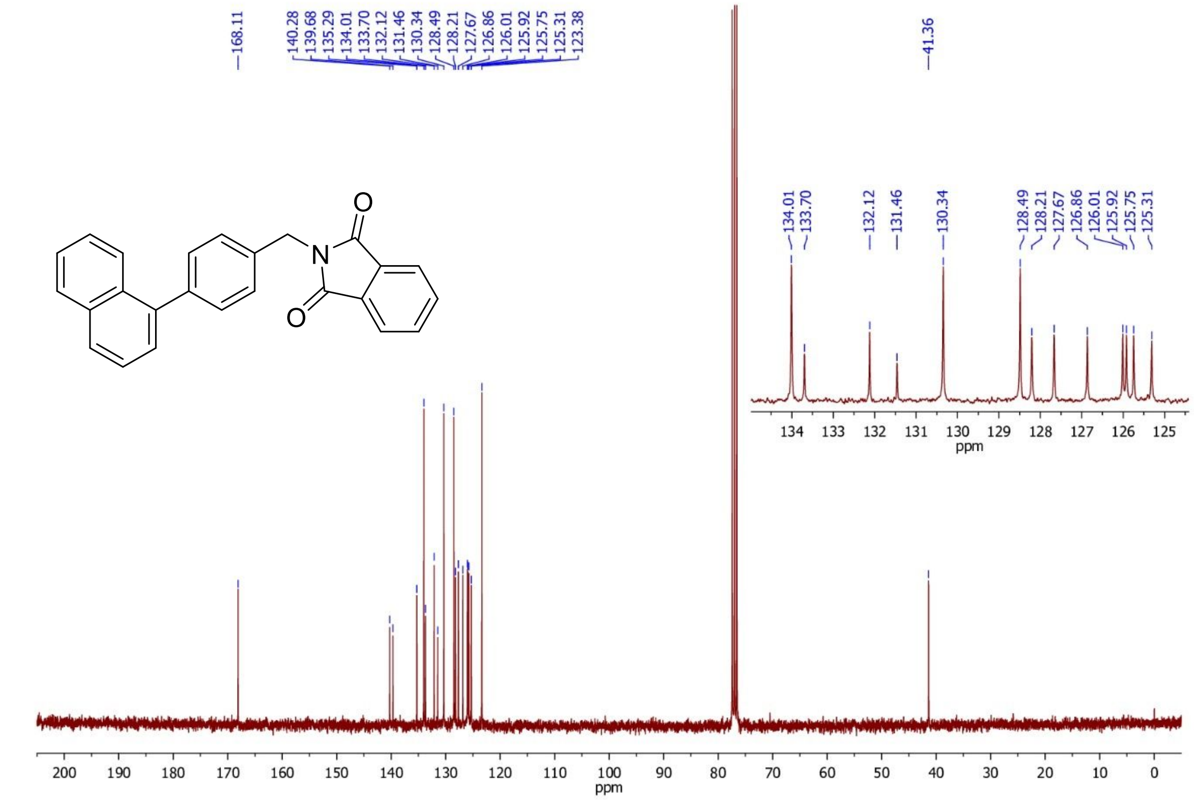

Figure S20. ^{1}H and ^{13}C NMR spectra of compound 3j

File . :C:\MSDChem\1\DATA\2023\Dr. Abbas Hassan\Nabiha Abdullah\ NAX-
... 9F 25-08-23.D
Operator : Saqib Yasin
Instrument : Instrument #2
Acquired : 25 Aug 2023 10:09 using AcqMethod LIQUID.M
Sample Name: NAX-9F
Misc Info : Temp 120-280 10C/min Flow 1.5ml/min Inj 5ul

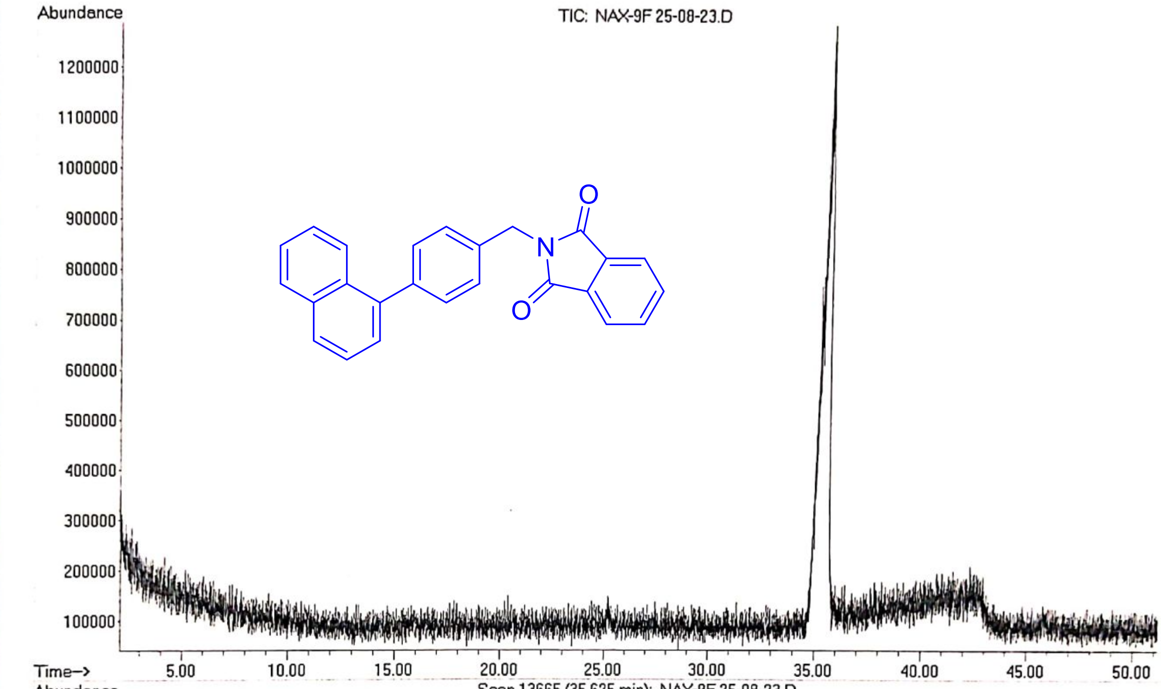

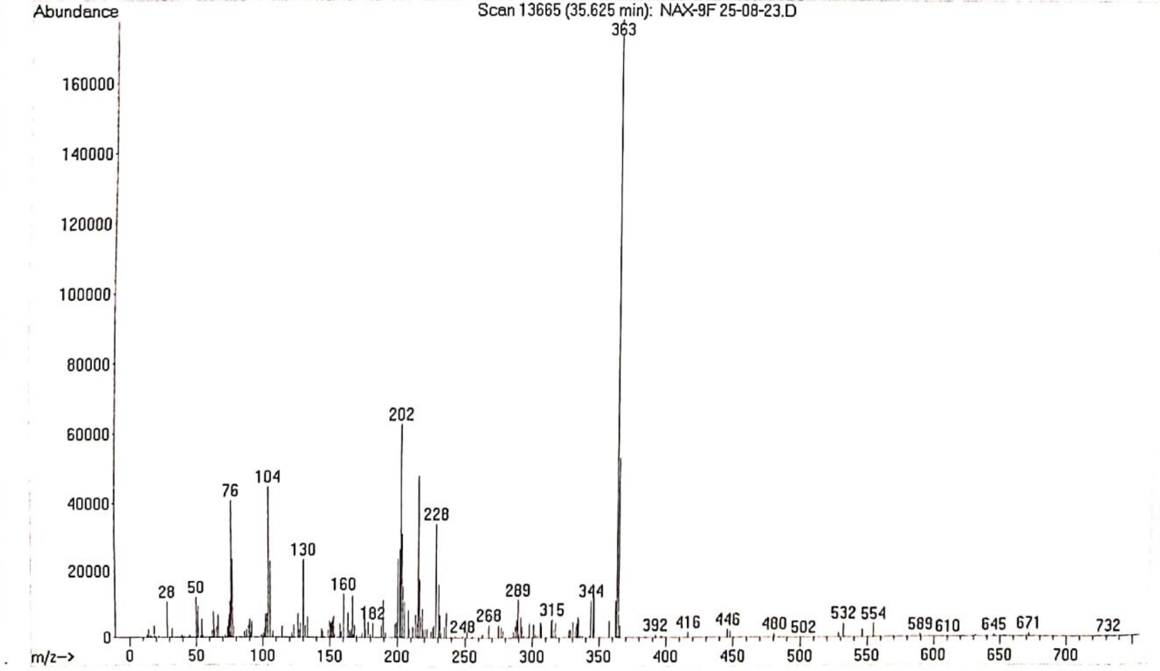

Figure S21. GC-MS of compound 3j

2-(4-(Benzo[*d*][1,3]dioxol-5-yl)benzyl)isoindoline-1,3-dione (3k)

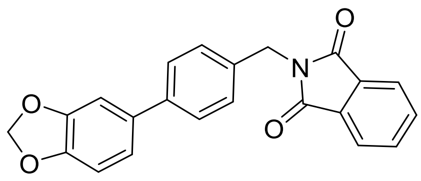

White solid, Yield = 93%, m.p. = 190-192 °C, R_f = 0.29 (1: 9 :: EtOAc: *n*-Hexane).

^{1}H NMR ($CDCl_3$, 300 MHz) $\delta_{(ppm)}$ = 7.85 (dd, *J* = 5.4, 5.4 Hz, 2H), 7.70 (dd, *J* = 5.7, 5.4 Hz, 2H), 7.50-7.43 (m, 4H), 7.00 (dd, *J* = 6.3, 6 Hz, 2H), 6.86-6.83 (m, 1H), 5.98 (s, 2H), 4.87 (s, 2H).

^{13}C NMR ($CDCl_3$, 75 MHz) $\delta_{(ppm)}$ = 168.0, 148.0, 147.1, 140.5, 140.4, 135.0, 134.0, 132.1, 129.0, 127.1, 123.3, 120.6, 108.5, 107.5, 101.1, 41.2.

GC-EIMS (m/z): 357 (100%), 339, 299, 252, 224, 190, 139, 104, 76, 50.

HRMS-ESI (m/z): $[M+H]^+$ calc'd for $C_{22}H_{16}NO_4^+$, 358.1074; found, 358.1077.

FTIR (neat): $\bar{\upsilon}$ (cm^{-1}) = 3075, 2913, 1709, 1608, 1495, 1394, 1234, 1032, 932, 785, 705, 530.

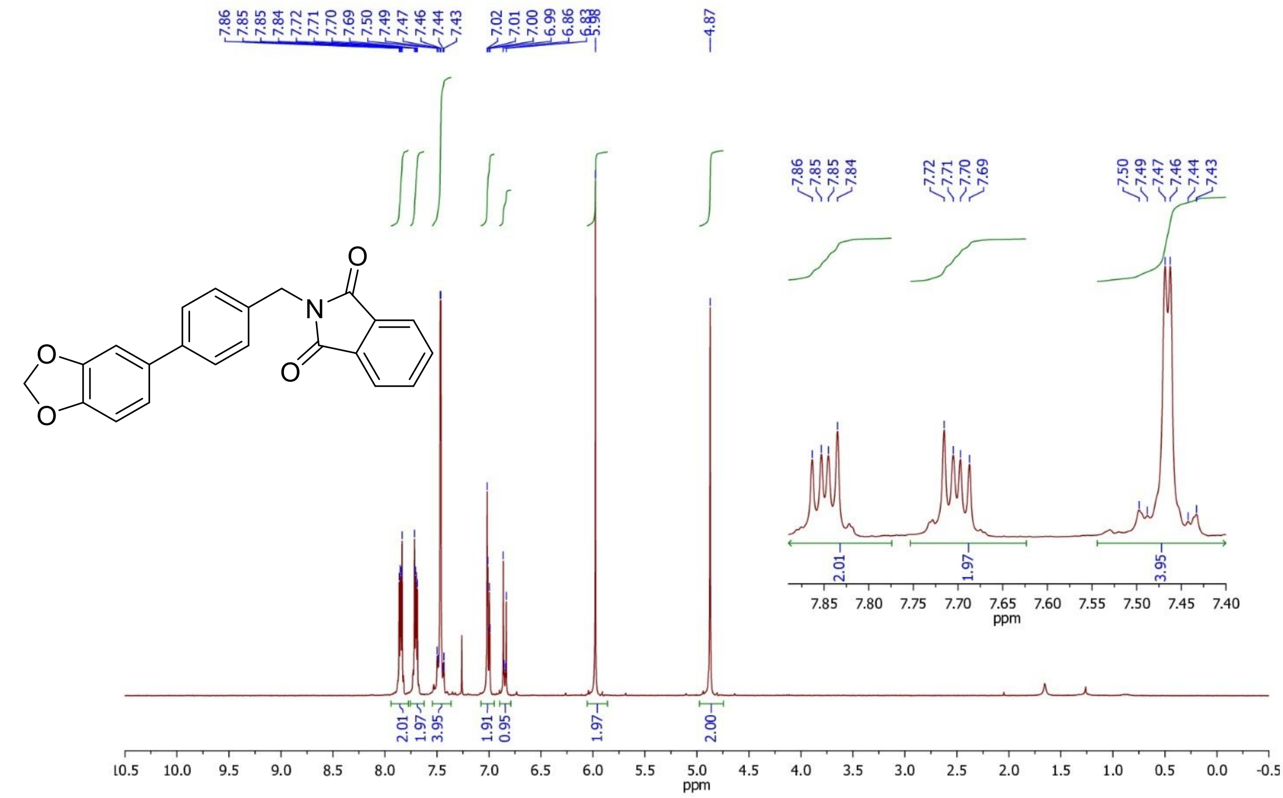

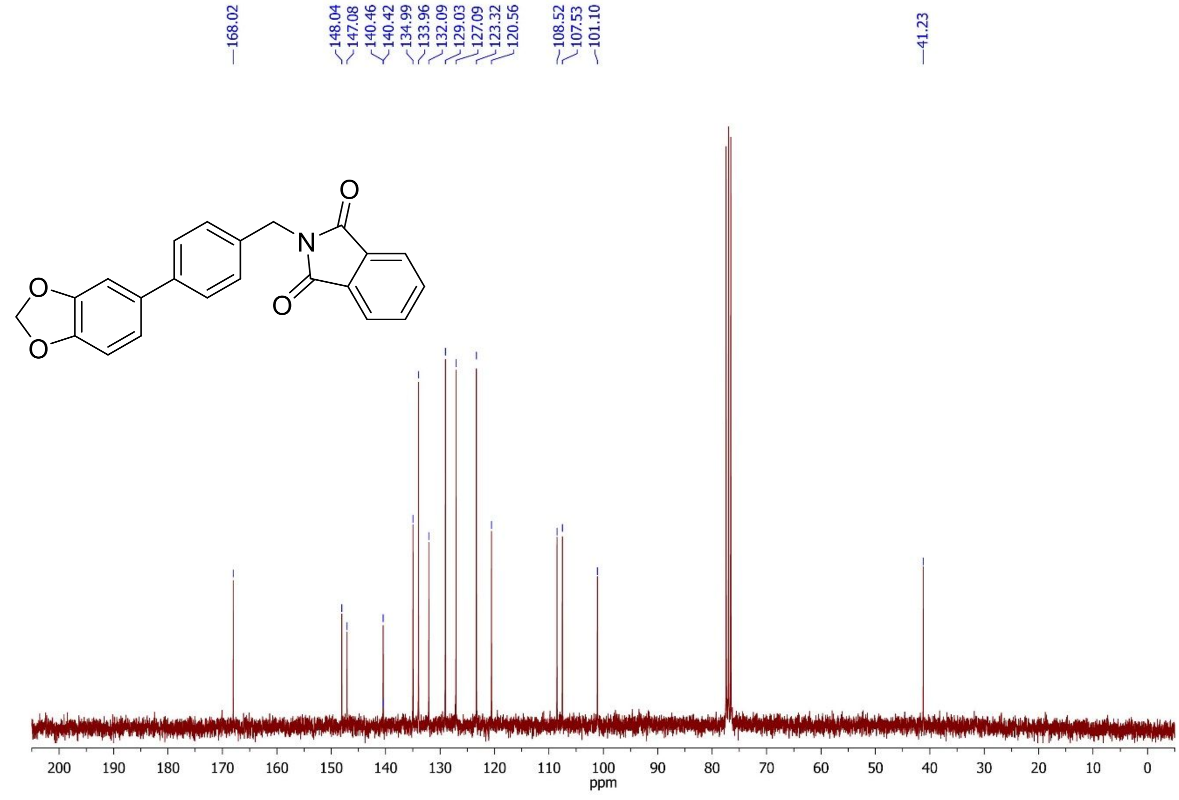

Figure S22. ^{1}H and ^{13}C NMR spectra of compound 3k

2-(4-(Thiophen-2-yl)benzyl)isoindoline-1,3-dione (3l)

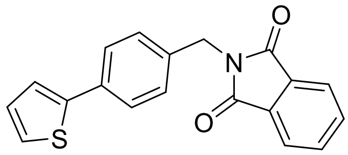

White solid, Yield = 70%, m.p. = 147-149 °C, R_f = 0.52 (1: 4 :: EtOAc: *n*-Hexane).

^{1}H NMR ($CDCl_3$, 300 MHz) $\delta_{(ppm)}$ = 7.92-7.88 (m, 2H), 7.79-7.75 (m, 2H), 7.63-7.60 (m, 2H), 7.51 (d, *J* = 8.1 Hz, 2H), 7.32 (dt, *J* = 4.2 ,0.9 Hz, 2H), 7.11 (dd, *J* = 5.1, 3.6 Hz, 1H), 4.91 (s, 2H).

^{13}C NMR ($CDCl_3$, 75 MHz) $\delta_{(ppm)}$ = 168.0, 143.8, 135.4, 134.0, 132.1, 129.2, 128.6, 128.0, 126.2, 124.9, 123.4, 123.2, 41.2.

GC-EIMS (m/z): 319 (100%), 291, 262, 186, 160, 115, 76, 50.

HRMS-ESI (m/z): $[M+H]^+$ calc'd for $C_{19}H_{14}NO_2S^+$, 320.0740; found, 320.0746.

FTIR (neat): $\bar{\upsilon}$ (cm^{-1}) = 3068, 2974, 2847, 1702, 1608, 1387, 1086, 939, 711, 530.

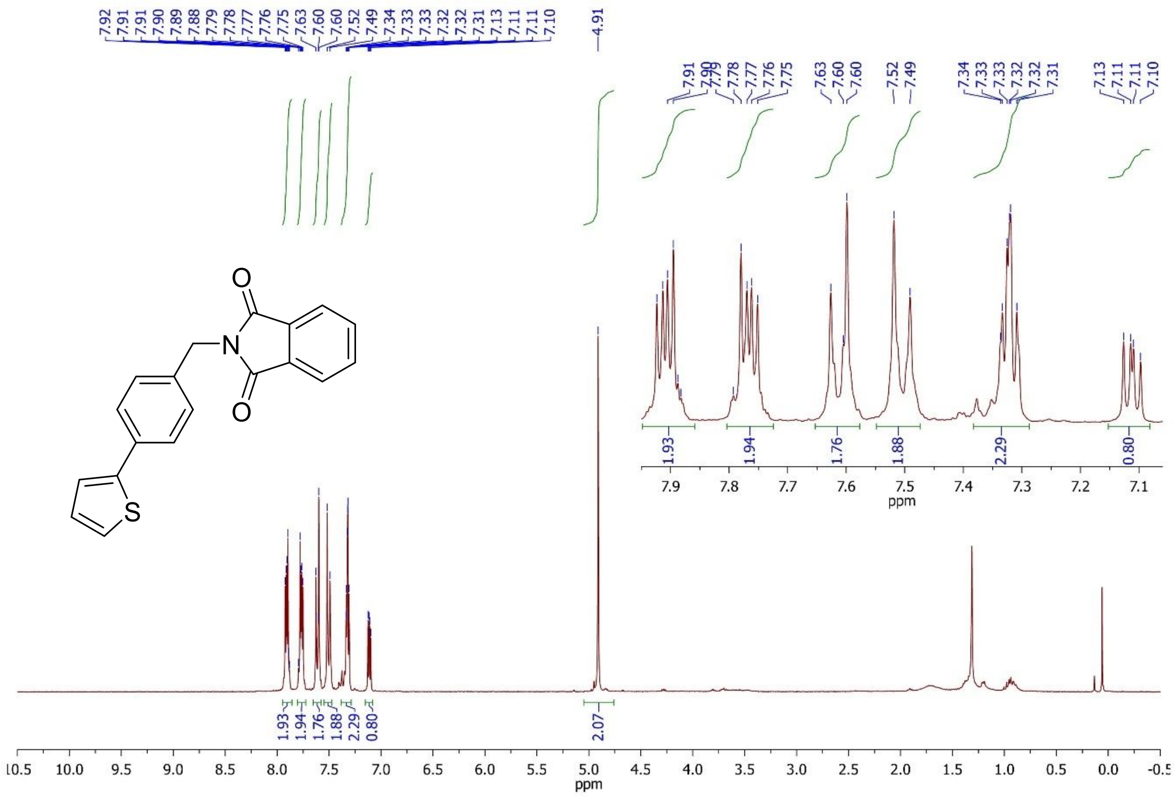

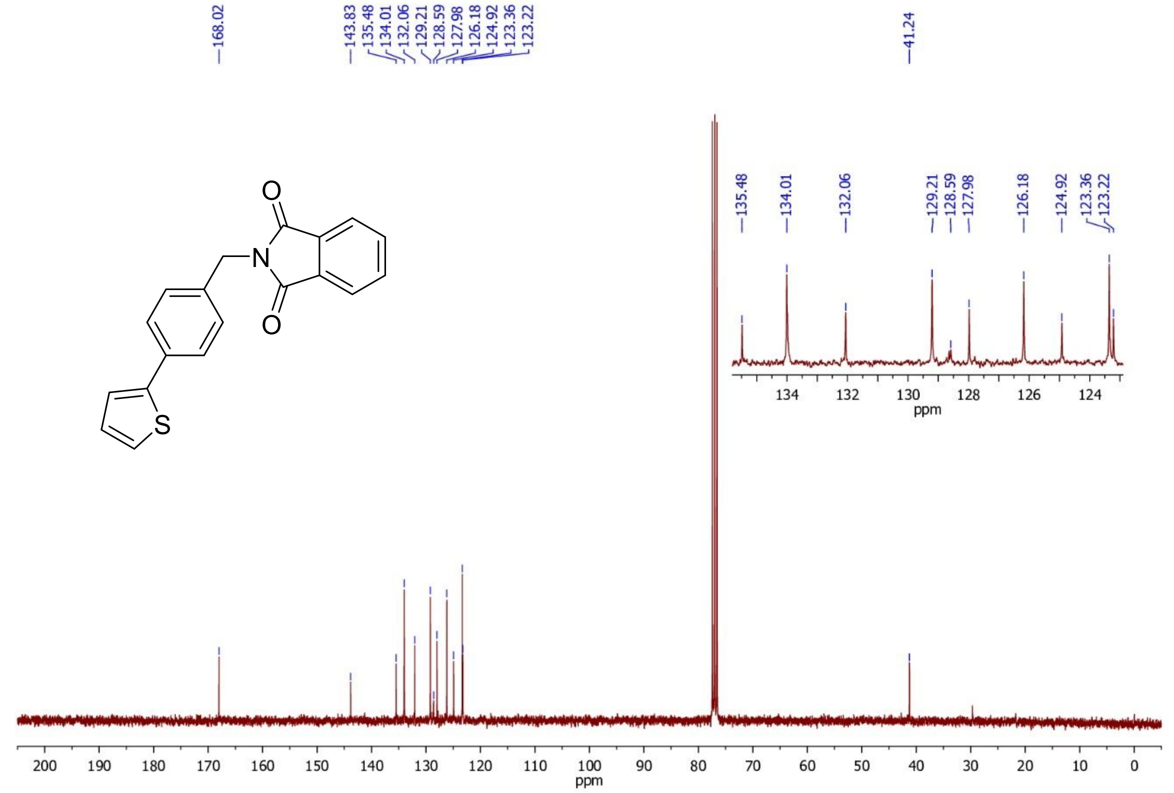

Figure S23. ^{1}H and ^{13}C NMR spectra of compound 3l

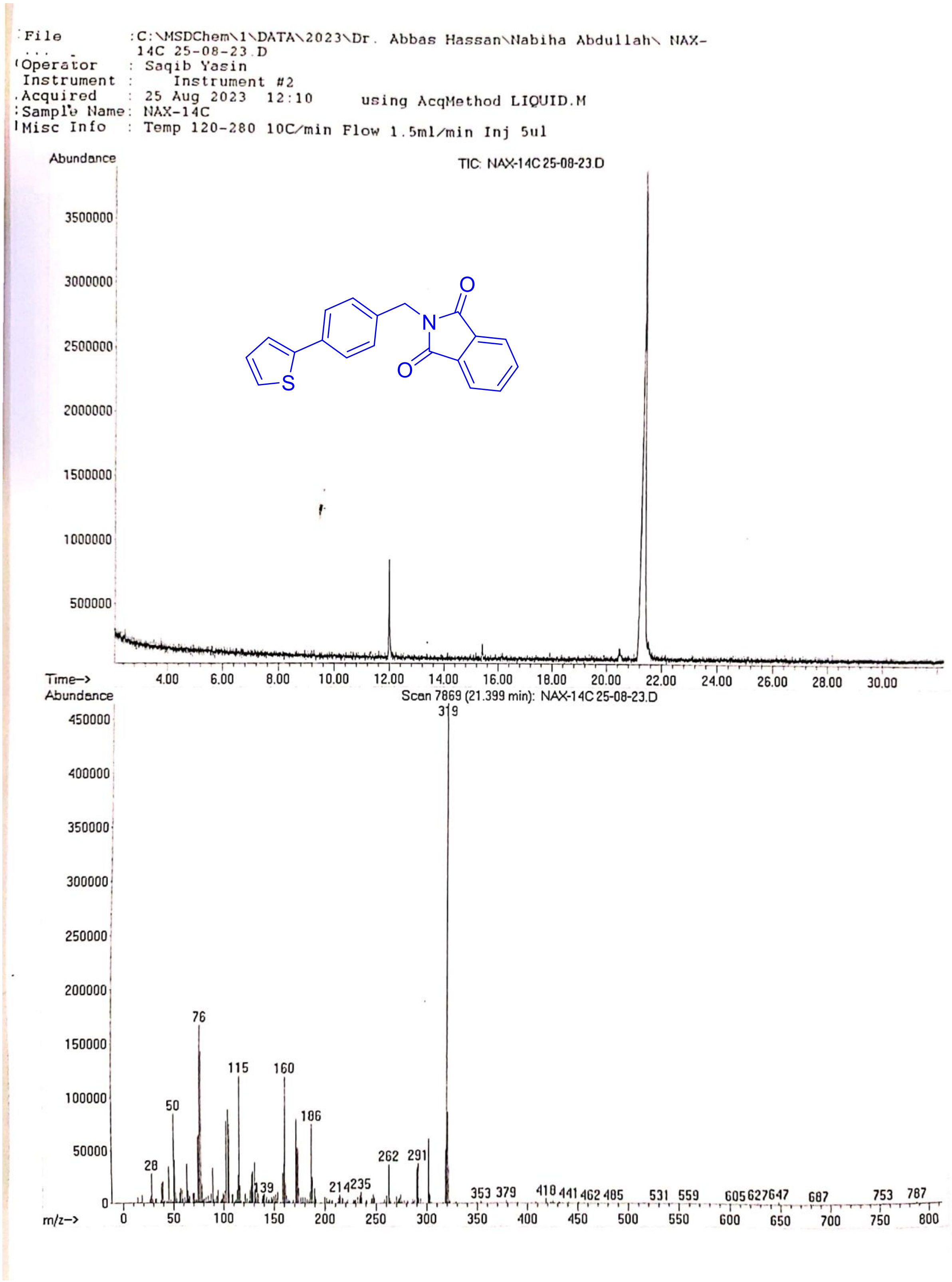

Figure S24. GC-MS of compound 3l

2-(4-(2-Methylquinolin-6-yl)benzyl)isoindoline-1,3-dione (3m)

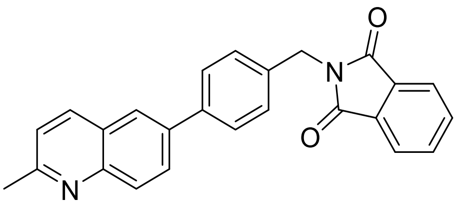

White solid, Yield = 43%, m.p. = 196-198 °C, R_f = 0.28 (1: 4 :: EtOAc: *n*-Hexane).

^{1}H NMR ($CDCl_3$, 300 MHz) $\delta_{(ppm)}$ = 7.94 (d, *J* = 8.7 Hz, 2H), 7.78-7.70 (m, 4H), 7.60-7.52 (m, 4H), 7.43 (d, *J* = 8.4 Hz, 2H), 7.17 (d, *J* = 8.4 Hz, 1H), 4.78 (s, 2H), 2.62 (s, 3H).

^{13}C NMR ($CDCl_3$, 75 MHz) $\delta_{(ppm)}$ = 168.0, 159.0, 147.2, 139.9, 137.8, 136.3, 135.6, 134.0, 132.0, 129.2, 129.0, 128.9, 127.6, 126.5, 125.2, 123.3, 122.4, 41.2, 25.3.

GC-EIMS (m/z): 378 (100%), 349, 288, 245, 219, 173, 141, 104, 78.

FTIR (neat): $\bar{\upsilon}$ (cm^{-1}) = 3028, 2927, 2847, 1702, 1595, 1394, 1086, 939, 718, 530.

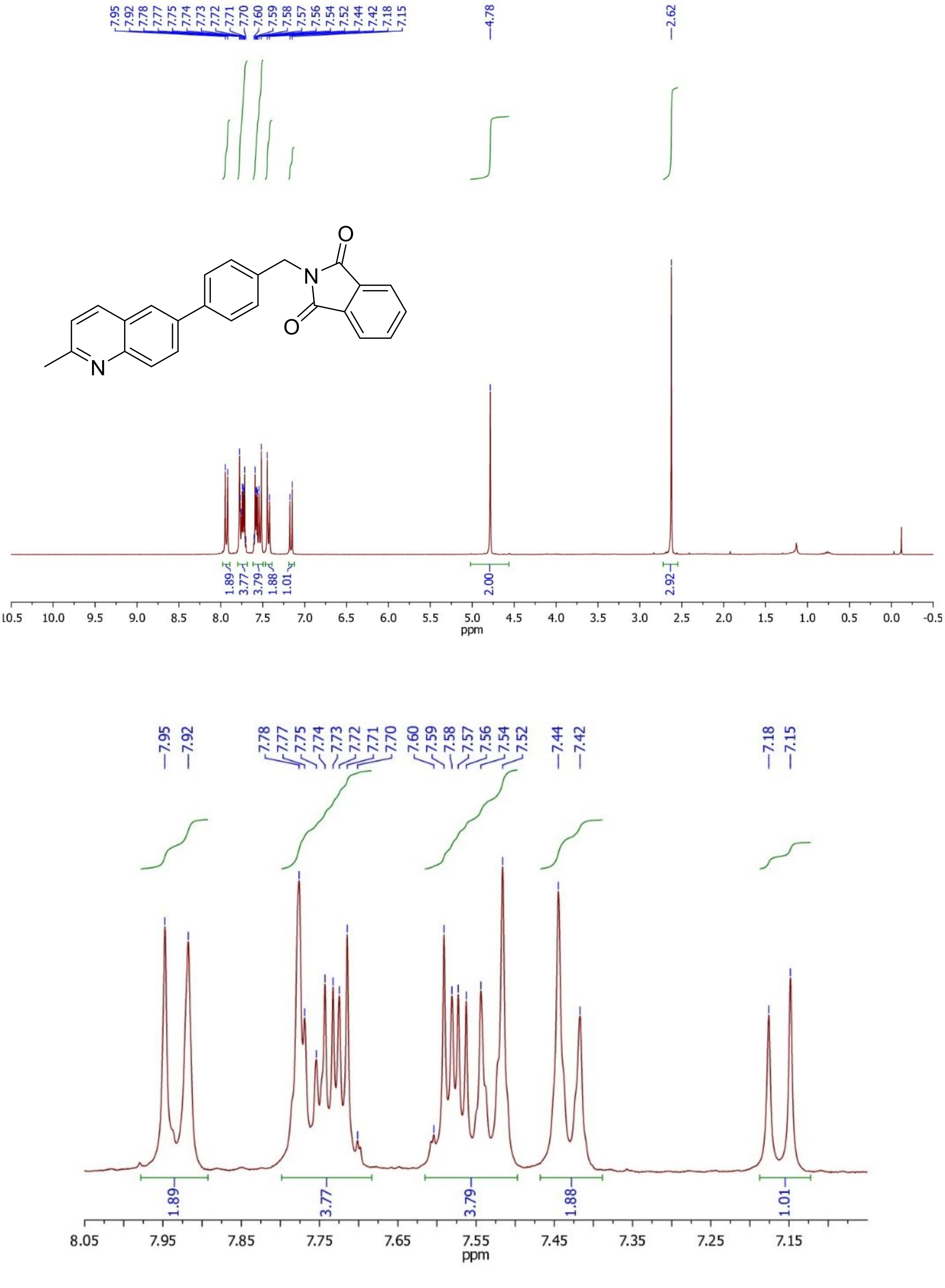

Figure S25. ^{1}H NMR spectra of compound 3m

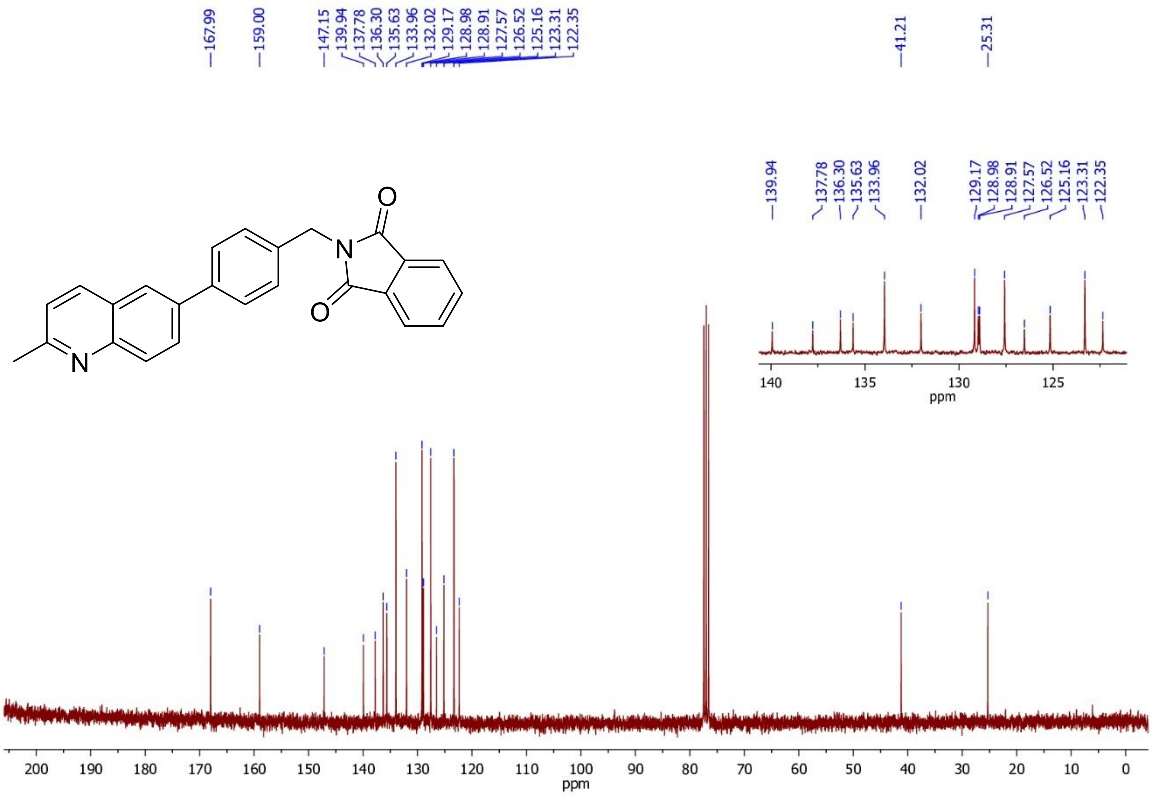

Figure S26. ^{13}C NMR spectrum of compound 3m

A

Ligand	D72	W84	G117	G118	Y121	S122	G123	Y130	Q199	W279	L282	S286	I287	F288	R289	F290	F330	F331	Y334	G335
3e	1	1	0	0	1	0	0	0	0	1	1	1	1	1	1	1	1	1	1	0
3j	1	1	0	1	1	0	0	0	0	1	1	1	1	1	1	1	1	1	1	1
3f	0	1	1	1	1	0	0	1	1	1	0	1	1	1	0	0	1	1	1	1
3g	0	1	1	1	1	0	0	0	1	1	0	1	0	1	1	1	1	1	1	0
3k	0	1	1	1	1	0	0	0	1	1	0	1	0	1	1	1	1	1	1	0
3g	0	1	1	1	1	1	1	1	0	1	0	0	1	0	0	0	1	1	1	0
3m	0	1	1	1	1	0	0	1	1	1	0	1	1	1	1	1	1	1	1	0
3d	0	1	1	1	1	0	0	0	1	1	1	1	0	1	1	1	1	1	1	0
3a	1	1	1	1	1	0	0	1	1	1	0	0	0	0	0	0	1	1	1	0
3b	1	1	0	0	1	0	0	0	0	1	1	1	0	1	1	1	1	1	1	0
3l	0	1	1	1	1	0	0	0	1	1	0	0	1	0	0	0	1	1	1	0
3m	0	1	1	1	1	1	0	1	0	1	0	1	0	1	1	1	1	1	1	0

No inteaction | Side chain | Backbone | Hydrophobic | H-Donor | H-Acceptor

B

Ligand	N68	I69	D70	S72	G78	W82	G116	G117	Q119	T120	Q197	S198	W231	T284	P285	L286	S287	V288	N289	A328	F329	Y332	W430	M437	H438	Y440
3l	0	0	0	0	0	1	1	1	0	0	0	1	1	0	0	1	1	1	0	0	1	1	1	1	1	1
3b	0	0	1	0	0	1	1	0	1	1	0	0	0	0	0	0	1	0	1	1	0	1	1	1	1	1
3g	0	0	1	0	0	1	1	0	1	1	0	0	0	0	0	0	1	0	1	1	0	1	1	1	1	1
3j	0	0	1	0	0	1	1	0	0	1	1	0	0	0	1	0	0	0	0	1	1	1	0	0	1	0
3m	1	0	1	0	0	1	1	0	1	1	0	0	0	0	0	0	1	0	1	1	0	1	1	1	1	1
3c	0	0	1	0	0	1	0	0	0	0	1	0	0	0	1	0	0	0	0	1	1	1	0	0	1	0
3e	0	0	1	0	0	1	1	0	1	1	0	0	0	0	1	0	1	0	1	1	0	1	1	1	1	1
3a	0	0	1	0	0	1	1	0	1	1	0	0	0	0	1	0	1	0	1	1	0	1	1	1	1	1
3f	0	0	1	0	0	1	1	0	1	1	0	0	0	0	1	0	1	0	1	1	0	1	1	1	1	1
3g	1	1	1	0	1	1	0	0	0	1	0	0	0	0	1	0	0	0	0	0	1	1	1	0	1	1
3k	0	1	1	0	0	1	1	0	0	0	1	0	0	0	1	0	0	0	0	1	1	1	0	0	1	0
3d	0	0	0	0	1	1	1	0	1	0	0	0	0	1	1	0	1	0	0	1	1	1	1	0	0	1

No inteaction | Side chain interaction | Backbone interaction | Hydrophobic | H-Donor | H-Acceptor

C

Ligand	R42	Y43	G57	G58	Y60	F168	L171	C172	I198	I199	G205	G206	C296	I316	Y326	F343	C397	Y398	G434	Y435	M436
3f	1	0	1	1	1	0	1	1	1	1	0	1	0	0	1	1	1	1	1	1	0
3b	1	0	1	1	1	1	1	1	1	1	0	1	0	0	0	1	1	1	1	1	0
3g	1	0	1	1	1	0	1	1	1	1	0	1	1	0	1	1	1	1	1	1	0
3a	1	0	1	1	1	1	1	1	1	1	0	1	0	0	0	1	1	1	1	1	0
3d	1	0	1	1	1	0	1	0	1	1	0	1	1	0	1	1	1	1	1	1	0
3j	1	0	1	1	1	0	1	1	1	1	0	1	0	0	1	1	1	1	1	1	0
3k	1	0	1	1	1	0	1	1	1	1	0	1	0	0	0	1	1	1	1	1	0
3c	1	0	1	1	1	0	1	0	1	1	0	1	0	0	1	1	1	1	1	1	0
3l	0	0	0	1	1	0	1	1	1	0	0	1	0	0	1	1	0	1	1	1	1
3e	1	1	0	1	1	0	1	1	1	1	0	1	0	0	1	1	0	1	1	1	1
3g	1	0	0	1	1	0	1	1	1	0	0	1	0	0	1	1	1	1	1	1	1
3i	1	0	1	1	1	0	1	0	1	1	0	1	0	0	1	1	1	1	1	1	0

No inteaction | Side chain interaction | Backbone interaction | Hydrophobic | H-Donor | H-Acceptor

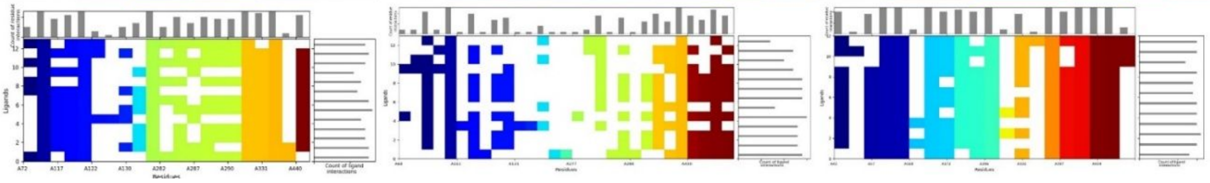

Figure S27. Structural fingerprinting analysis of thirteen docked compounds with (A) AChE (B) BChE (C) MAO-B

Table S1. Docking scores of standards and newly synthesized compounds docked against AChE, BChE, and MAO-B.

**Ligands**	**AChE**	**BChE**	**MAO-B**
**Standard**	**-13.443**	**-9.199**	**-11.616**
3e	-9.885	-8.333	-10.219
3f	-9.453	-5.807	-11.876
3g	-9.347	-6.596	-10.556
3k	-9.334	-5.158	-10.165
3h	-9.266	-5.201	-7.19
3m	-9.262	-6.068	-11.677
3c	-8.873	-4.255	-10.324
3a	-8.779	-5.832	-10.363
3b	-8.769	-6.832	-10.83
3l	-8.752	-6.199	-9.236
3j	-8.394	-5.924	-7.671
3d	-6.551	-6.003	-9.89
3i	-7.32	-6.234	-8.213
